# Comparative Analysis of the Two *Acinetobacter baumannii* Multilocus Sequence Typing (MLST) Schemes

**DOI:** 10.3389/fmicb.2019.00930

**Published:** 2019-05-03

**Authors:** Stefano Gaiarsa, Gherard Batisti Biffignandi, Eliana Pia Esposito, Michele Castelli, Keith A. Jolley, Sylvain Brisse, Davide Sassera, Raffaele Zarrilli

**Affiliations:** ^1^Microbiology and Virology Unit, Fondazione IRCCS Policlinico San Matteo, Pavia, Italy; ^2^Department of Biology and Biotechnology, University of Pavia, Pavia, Italy; ^3^Department of Public Health, University of Naples “Federico II”, Naples, Italy; ^4^Romeo and Enrica Invernizzi Pediatric Research Center, Department of Biosciences, University of Milan, Milan, Italy; ^5^Department of Zoology, University of Oxford, Oxford, United Kingdom; ^6^Biodiversity and Epidemiology of Bacterial Pathogens, Institut Pasteur, Paris, France

**Keywords:** multilocus sequence typing, *Acinetobacter baumannii*, comparative genomics, phylogeny, sequence types, clonal complexes

## Abstract

*Acinetobacter* species assigned to the *Acinetobacter calcoaceticus-baumannii* (Acb) complex, are Gram-negative bacteria responsible for a large number of human infections. The population structure of Acb has been studied using two 7-gene MLST schemes, introduced by Bartual and coworkers (Oxford scheme) and by Diancourt and coworkers (Pasteur scheme). The schemes have three genes in common but underlie two coexisting nomenclatures of sequence types and clonal complexes, which complicates communication on *A. baumannii* genotypes. The aim of this study was to compare the characteristics of the two schemes to make a recommendation about their usage. Using genome sequences of 730 strains of the Acb complex, we evaluated the phylogenetic congruence of MLST schemes, the correspondence between sequence types, their discriminative power and genotyping reliability from genomic sequences. *In silico* ST re-assignments highlighted the presence of a second copy of the Oxford *gdhB* locus, present in 553/730 genomes that has led to the creation of artefactual profiles and STs. The reliability of the two MLST schemes was tested statistically comparing MLST-based phylogenies to two reference phylogenies (core-genome genes and genome-wide SNPs) using topology-based and likelihood-based tests. Additionally, each MLST gene fragment was evaluated by correlating the pairwise nucleotide distances between each pair of genomes calculated on the core-genome and on each single gene fragment. The Pasteur scheme appears to be less discriminant among closely related isolates, but less affected by homologous recombination and more appropriate for precise strain classification in clonal groups, which within this scheme are more often correctly monophyletic. Statistical tests evaluate the tree deriving from the Oxford scheme as more similar to the reference genome trees. Our results, together with previous work, indicate that the Oxford scheme has important issues: *gdhB* paralogy, recombination, primers sequences, position of the genes on the genome. While there is no complete agreement in all analyses, when considered as a whole the above results indicate that the Pasteur scheme is more appropriate for population biology and epidemiological studies of *A. baumannii* and related species and we propose that it should be the scheme of choice during the transition toward, and in parallel with, core genome MLST.

## Introduction

Bacteria belonging to the genus *Acinetobacter* are glucose non-fermentative Gram-negative coccobacilli that are a frequent cause of health-care associated infections and hospital outbreaks, especially in intensive-care unit patients (Dijkshoorn et al., 2007; Zarrilli et al., 2013). *A. baumannii*, *A. nosocomialis*, *A. pittii*, *A. seifertii*, and *A. dijkshoorniae*, five of the most clinically relevant species, are genetically and phenotypically similar to the environmental species *A. calcoaceticus* and are therefore grouped into a species complex called the *A. calcoaceticus-A. baumannii* (Acb) complex (Dijkshoorn et al., 2007; Zarrilli et al., 2013; Marí-Almirall et al., 2017). *Acinetobacter spp*. isolates responsible for epidemics, in particular *A. baumannii* isolates, are frequently multidrug resistant (MDR) or extensively drug resistant (XDR). The majority of these strains are resistant to carbapenems and a fraction of them are resistant to last resource antimicrobial agent colistin (Zarrilli et al., 2013; Pournaras et al., 2017).

The rise of resistant Acb strains prompted the design and execution of epidemiological investigations of *A. baumannii* epidemics using a variety of molecular typing methods, among which multilocus sequence typing (MLST) has become the reference approach (Dijkshoorn et al., 1996; Dijkshoorn et al., 2007; Zarrilli et al., 2013). Among the advantages of MLST are its excellent reproducibility, its portability that allows global comparisons, and the ease of interpretation of data in evolutionary terms (Maiden et al., 1998; Maiden et al., 2013; Bialek-Davenet et al., 2014). Besides, a prominent benefit of MLST is a derived nomenclature of sequence types (STs), which have been rapidly and largely adopted by the community, allowing expansion of the global collective knowledge on the distribution, spread and biological features of the major clonal groups.

For *A. baumannii*, the advantages of an MLST nomenclature have been somewhat reduced by the co-existence of two MLST schemes, which are both widely used. Both schemes encompass *A. baumannii* and non*-baumannii Acinetobacter* species. The first scheme was introduced by Bartual and coworkers and is referred as the Oxford scheme, after the platform hosting it (Bartual et al., 2005; Wisplinghoff et al., 2008), whereas a second scheme was later published by Diancourt and coworkers (Pasteur scheme) (Diancourt et al., 2010). Although both schemes appeared to provide largely concordant classifications (Zarrilli et al., 2013, 2015), the co-existence of two nomenclatures (Zarrilli et al., 2015) calls for an assessment of their relative merits in terms of reliability, discrimination (which should be optimized for epidemiological purposes) and phylogenetic concordance of their derived classifications with “true” phylogenic relationships. Although the schemes were initially hosted at two different locations (both using first the mlstdbnet; Jolley et al., 2004) then the BIGSdb software (Jolley and Maiden, 2010), in 2013, the two schemes were united into a single database. This move facilitated curation requests (sometimes using both schemes for the same set of isolates) and harmonized the data analysis functionalities. The hosting of both schemes within a single BIGSdb database, which can incorporate genomic sequences, facilitated the joint MLST analysis of genomic sequences using both schemes.

Molecular epidemiology investigations revealed the occurrence of genetically distinct clonal lineages among populations of *A. baumannii* (Diancourt et al., 2010; Zarrilli et al., 2013). Three of these lineages, which were initially defined as European clones I to III and subsequently regarded as International Clones (IC) I to III, are distributed worldwide (IC I and IC II are also known as Global Clones, GC). The Pasteur scheme genotypes were numbered according to previous denominations, i.e., IC I, II, and III were named, respectively, as CC1, CC2, and CC3, with their dominant ST named ST1, ST2, and ST3, respectively (Dijkshoorn et al., 1996; van Dessel et al., 2004; Diancourt et al., 2010; Zarrilli et al., 2013). Other successful epidemic clonal lineages have been subsequently identified in the population structure of *A. baumannii* using the Pasteur MLST scheme, including sequence types ST10, ST15, ST25, ST32, ST78, ST79 (Diancourt et al., 2010; Zarrilli et al., 2013; Da Silva et al., 2014; Pournaras et al., 2014; Ou et al., 2015; Sahl et al., 2015). The Oxford MLST scheme was able to identify international clones I, II, and III and has been shown to possess higher discriminatory power than the Pasteur scheme (Feng et al., 2016; Tomaschek et al., 2016), but to suffer from problems due to recombination and technical artifacts (Hamouda et al., 2010; Hamidian et al., 2017). Recombination plays a crucial role in the evolution of the Acb genomes. Several specific loci are interested by this phenomenon. Among them is the *gpi* gene, which is part of the capsular operon (thus influencing the bacterium virulence) and one of the seven Oxford MLST scheme genes. Several works suggested to exploit this behavior for classification and adopt the Oxford scheme, as it allows to monitor the capsular type (Kenyon and Hall, 2013; Holt et al., 2016; Schultz et al., 2016; Hamidian et al., 2019).

The aims of the present study were to recapitulate the current status of both schemes, determine the characteristics of the Oxford and the Pasteur MLST schemes in terms of reliability of genotyping, denomination correspondence, phylogenetic congruence with genome-based phylogenies and discriminatory power.

## Materials and Methods

### Python and Perl Scripts

All the scripts specifically developed and used for this work are available at https://github.com/MIDIfactory.

### MLST Data

On 14 September 2018, we retrieved all sequence and profile definitions of both schemes from the PubMLST database^1^ to evaluate them comparatively using different approaches.

### Genome Datasets

Bacterial genomes included in the analysis were manually selected from the PubMLST database. In detail, we selected all complete genomes and all high-quality genomes, i.e., in which all loci of the MLST schemes and the ribosomal MLST scheme (Jolley et al., 2012) could be detected. The resulting dataset contains the genomes of 730 strains, belonging to the Acb complex, i.e., *A. baumannii* (*n* = 703), *A. nosocomialis* (*n* = 13), *A. seifertii* (*n* = 1), *A. dijkshoorniae* (*n* = 1), *A. pittii* (*n* = 7), *A. calcoaceticus* (*n* = 3) (see Supplementary Figures S1A,B for geographical and temporal distribution of the isolates, respectively). A complete list of the genomes is available here: https://pubmlst.org/bigsdb?db=pubmlst_abaumannii_isolates&page=projects.

The allelic variants of all gene fragments of both schemes were extracted from all the genomes, using an in-house Python script based on Blast (Altschul et al., 1990), keeping all results above 95% of identity with known alleles and subsequently selecting only perfect matches, procedure that allowed to assign the corresponding STs. The allelic sequences obtained were then aligned with Muscle (Edgar, 2004). The resulting alignments were concatenated using an in-house Perl script, to obtain two multigene alignments (one per MLST scheme) to be used as input for downstream analyses.

A core genome alignment was obtained to be used as a reference for determining the reliable phylogenetic trees. Gene calling was performed using Prodigal software v2.6.1 (Hyatt et al., 2010) on all 730 genomes in the dataset. A Perl script, which uses the double best Blast hit algorithm, was then used to identify genes orthologs to the previously published core genome by Higgins et al. (2017). Groups of ortholog genes were built and aligned using Muscle (v3.8.31, Edgar, 2004). The resulting core alignments were polished for poorly aligned positions and divergent regions using Gblocks software version 0.91b (Castresana, 2000), and merged in a concatenate of all ortholog genes, via another Perl script.

A Single-nucleotide polymorphism (SNP) alignment was built as a second reference. SNPs were detected using the procedure developed by Gaiarsa and coworkers (Gaiarsa et al., 2015) based on the software Mauve (Darling et al., 2004). Each genome was individually aligned to a reference (the complete genome AB307-0294), and alignments were then concatenated. Core SNPs were defined as single-nucleotide mutations flanked by conserved bases present in all the genomes in analysis.

### Phylogenetic Analyses

Phylogenies of all four datasets (Oxford, Pasteur, core genes, core SNPs) were inferred using the same approach. The best model of evolution was determined using ModelTest-ng version 0.1.3 (Darriba et al., 2011). The selected model was GTRGAMMAIX for the three gene datasets, while the analysis for the SNP alignment was performed considering the ascertainment bias and using the Lewis correction (Lewis, 2001), thus with model ASC_GTRGAMMAX. Maximum Likelihood phylogeny was performed using RAxML (Stamatakis, 2014) with 100 bootstrap replicates.

### Statistical Analysis

Three statistical tests were performed using the Core-genome and SNP phylogenetic trees as references and comparing them to the phylogenetic trees resulting from MLST gene concatenates. Two topology-based tests (Matching clusters, Robinson-Fould), were performed with TreeCmp (Bogdanowicz et al., 2012). The matching clusters test calculates the number of topology changes that should be performed in order to transform a tree into the reference one. The Robinson-Fould (R-F) test instead counts the different bipartitions between the two trees. In both cases, a value of zero indicates that the two analyzed trees are identical.

The other analysis, likelihood-based Shimodaira-Hasegawa test, was performed with RaxML (Stamatakis, 2014). In this test, a null hypothesis is stated, which assumes that two compared trees are both a correct interpretation of an alignment. The tested hypothesis is that one or more trees are a better representation of the data. *P*-values smaller than 0.05 indicate that two trees are significantly different.

In addition, the Gini-Simpson index was used to determine the discrimination power of both schemes. The index was calculated using the website service comparingpartitions.info on the entire dataset of 730 genomes and on the genomes of the three main International Clones.

### Monophyly of Clonal Complexes

Clonal Complexes were defined using eBURST as described previously (Feil et al., 2004); CCs were defined as groups of Sequence Types that differ from one or more members of the group by just one allele. Monophyly of the CCs was checked on the core genome tree using the R environment and the library spider. Minimum spanning trees of sequence types (STs) were built using Phyloviz Online using the goeBURST algorithm (Ribeiro-Gonçalves et al., 2016). Minimum spanning trees were generated from the 7 alleles of each MLST scheme and species were assigned based on clustering with reference STs. The species field was updated in the PubMLST database for all STs with no ambiguous assignment.

### Nucleotide Distance Analysis

To evaluate how well each gene fragment variation correlates with the genome variation, we compared the pairwise nucleotide distances between each pair of genomes in our dataset with the corresponding distances between each gene fragment pairs. The MLST gene fragment sequences, as well as all the concatenated core genome gene sequences, were aligned with Muscle (Edgar, 2004) and then used to calculate pairwise genetic distances via the function “dist.dna” of the R package APE (Paradis et al., 2004).

In order to test the discrimination resolution of each MLST scheme, we plotted the pairwise sequence distance between each MLST locus for each pair of genomes, against the corresponding core genome-wide sequence distance. The correlation was determined adopting the regression linear model in the R environment. Due to an uneven large distribution of genomic distances, we decided to split each dataset in three blocks (as in Bleidorn and Gerth, 2018), based on the genomic distance (first block: 0.0–0.05, second block: 0.05–0.1, third: >0.1). Finally, a heatmap was generated to evaluate *R*^2^ and slopes of all regression lines.

### Recombination Rate

The recombination rate was calculated for each locus of both MLST schemes, using the RDP4 Software on the alignments of all alleles present the 730 genomes of the dataset (Martin et al., 2015), employing all available algorithms. This same analysis was also performed on a reduced dataset containing only the *A*. *baumannii* genome subset, to refine the recombination rate detection within this species.

### *GdhB* Analysis

To investigate the Oxford scheme gene *gdhB* putative duplication, we extracted all variants of both gene copies from the 730 genomes in our dataset, including sequences not registered in the PubMLST database, through a custom approach based on Blast (identity > 95% with the *gdhB*-1 or the *gdhB*-182 allele).

To check primer alignment, the flanking region was extracted for all alleles found, and aligned with the PCR primers used to sequence the *gdhB* locus. Mismatches between the aligned sequences and the primers were calculated for the entire primer length and for the ten bases at the three-prime end.

Finally, all *gdhB* and *gdhB2* variants were analyzed with a phylogenetic approach. Variants were aligned with the software muscle (Edgar, 2004) and used as input for a Maximum Likelihood phylogeny, executed with fasttree (Price et al., 2010). The genes surrounding the *gdhB* and *gdhB2* sites were extracted using a python script and the software Prodigal (Hyatt et al., 2010). Functions were predicted using COGnitor (Galperin et al., 2015). Codon adaptation index was calculated for all coding sequences in all 730 genomes in the dataset using the CAIcalc script (Puigbo et al., 2008).

## Results

### Status of the Database Contents of the Pasteur and Oxford MLST Schemes

**FIGURE 1:**
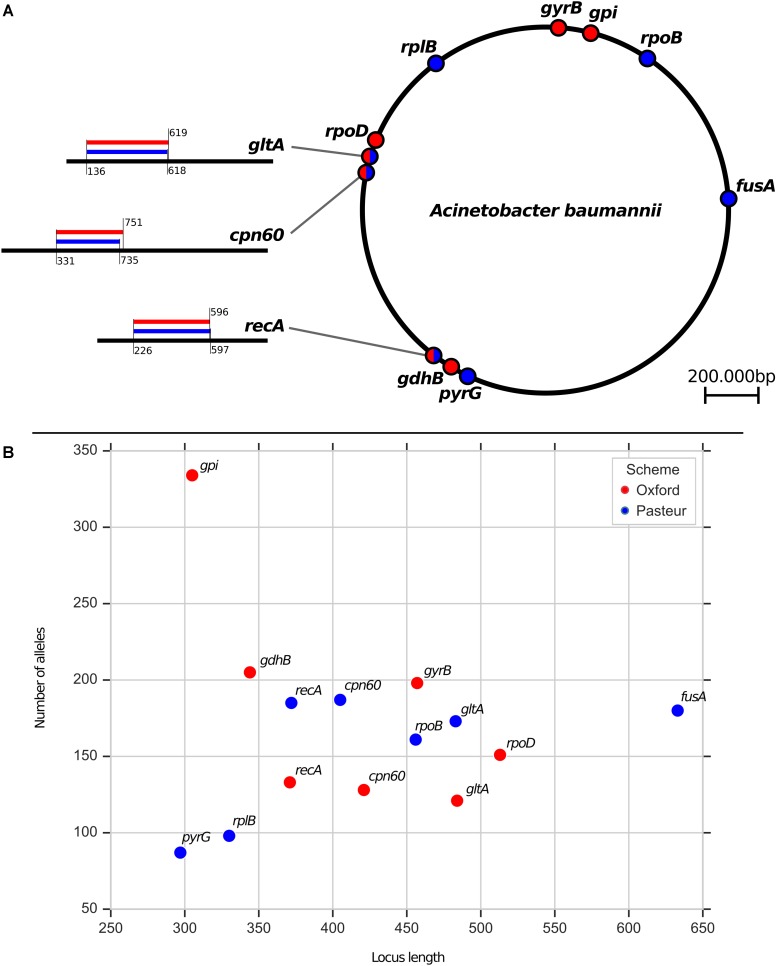
**(A)** Distribution of the MLST loci on the genome. A panel is used to highlight the relative position of two loci whenever they come from the same gene. **(B)** Scatterplot comparing loci length and variability in terms of number of alleles registered on the Pubmlst database (https://pubmlst.org/abaumannii).

In order to evaluate the two MLST schemes available for *A. baumannii*, we start by describing them in detail. Both schemes are built on 7 genes. The concatenation of allele 1 of the 7 Oxford and Pasteur genes yields 2895 and 2976 nucleotides, respectively. Three genes are shared between the two schemes: *cpn60*, *gltA*, and *recA*. The subsequences used for typing, though, differ between the two schemes, as depicted in Figure 1A.

We downloaded all sequence and profiles definitions on date 14 September 2018. Oxford contained 1866 profiles (STs), while the Pasteur scheme had 1234 STs defined. The number of alleles ranged from 121 to 334 for Oxford, 87–187 for Pasteur. Regarding the three common genes, the allele numbers were 128(Oxford)/187(Pasteur) for *cpn60*, 121(Oxford)/173(Pasteur) for *gltA*, 133(Oxford)/185(Pasteur) for *recA*. Thus, the Pasteur scheme seemed to encompass more diversity in the common genes, although more STs were defined by Oxford overall (Figure 1B).

### A gdhB Paralog Complicates *in silico* Determination of the Oxford ST

**FIGURE 2:**
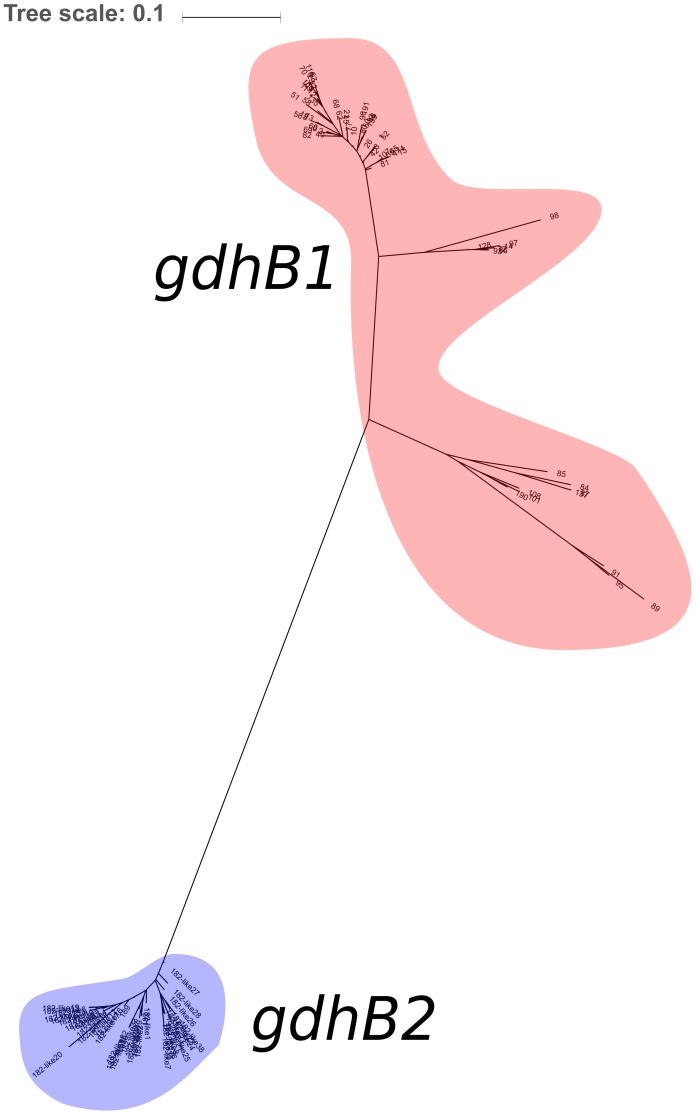
Maximum Likelihood phylogeny of the 104 *gdhB* variants detected in the dataset. Alleles of the traditional *gdhB* locus are highlighted in red; alleles of the alternative locus (including 38 non-registered alleles) are highlighted in blue.

730 genomes of the Acb complex were selected and downloaded to be used as dataset. The database included mostly genomes of *A. baumannii* isolates (703, 96.3%), but also genomes of *A. nosocomialis, A. seifertii, A. dijkshoorniae, A. pittii*, and *A. calcoaceticus* isolates. MLST alleles of both schemes were first defined from genomic sequences. While extracting alleles, a consistent proportion of the genomes appeared to have two variants of the *gdhB* gene. This issue was investigated further, revealing that an alternative *gdhB* locus (corresponding to alleles 182, 189 and variants of them) was present in 553 (76%) of 730 strains, all belonging to the *A. baumannii* species. This locus is often annotated in these genomes as *gdhB2* and has a sequence similarity with allele 1 of *gdhB* ranging from 73.98 to 77.94%. Primers used for molecular MLST were aligned to both genomic regions, showing a low affinity for the alternative *gdhB2* locus (Table 1). For this reason, we can hypothesize that the *gdhB2* locus cannot be amplified with these PCR primers, and indeed all alleles of the paralogous locus were defined only using *in-silico* methods for ST determination from genomic sequences. In order to evaluate the relationships between *gdhB* and *gdhB2*, a phylogeny of all 64 *gdhB* and the 40 *gdhB2* alleles was determined. The resulting tree clearly showed two main clusters, one containing only the putative *gdhB2* sequences, i.e., allele 182 and related variants, only found in *A. baumannii* (Figure 2). The other main cluster, containing the original *gdhB* variants, on the other hand presents genomes from all the analyzed Acb species, and each species appears grouped in a monophyletic cluster. The genomic surroundings of the two variants (i.e., the three genes upstream and downstream the *gdhB* and *gdhB2* sites) are clearly different. Nucleotide composition analysis was performed on *gdhB* and *gdhB2*, showing that both genes have a codon composition that is significantly different from the average of the respective genomes. A subset of isolates that are characterized in the Oxford scheme with STs that include alleles of *gdhB2* instead of the original locus where manually analyzed. When replacing the artefactual *gdhB2* allele with the correct *gdhB* locus, the obtained profiles correspond to existing STs, including ST231, a strain of epidemiological importance due to the report of the presence of the carbapenemase gene *bla*_OXA-23_. Therefore, the wrong calling of alleles at *gdhB2* locus has artifactually inflated the diversity recorded using the Oxford scheme.

**Table 1:** Minimum and maximum number of mutations obtained when sequencing primers are aligned to all *gdhB* loci in the dataset.

	Entire sequence		Last 10 nt	
	Forward primer	Reverse primer	Forward primer	Reverse primer
*gdhB* site	0–1	3–4	0–1	1–3
alternative *gdhB* site	4–18	6–6	3–10	4–4

### Oxford and Pasteur Comparison

**FIGURE 3:**
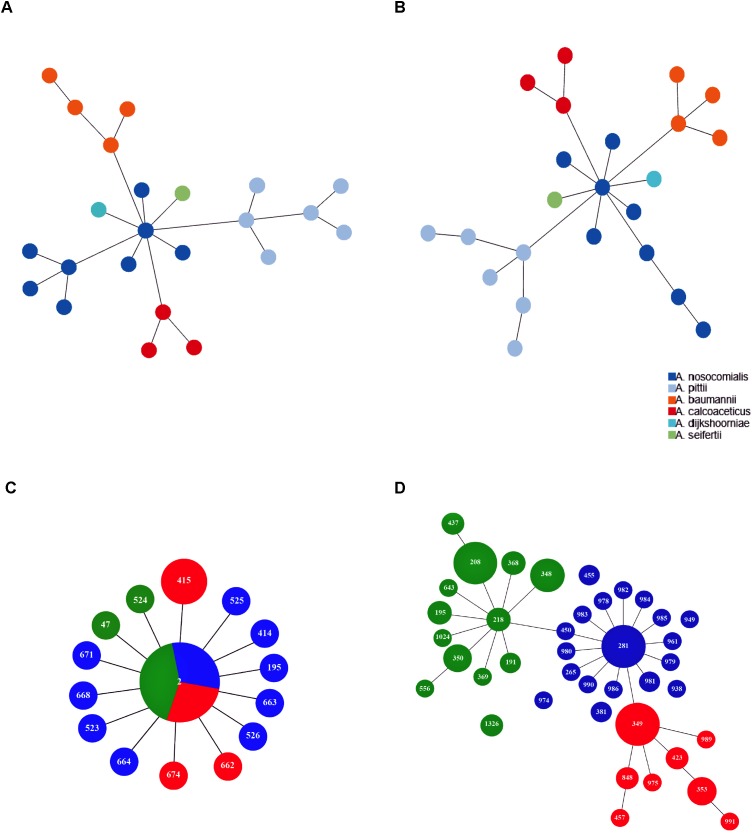
Minimum spanning trees of *A. baumannii*, *A. nosocomialis*, *A. pittii*, *A. seifertii*, and *A. dijkshoorniae* (23 isolates) using **(A)** Pasteur and **(B)** Oxford MLST scheme. The colors corresponding to *Acinetobacter* species are shown in the legend. Minimum spanning trees representing the structure of the *A. baumannii* international clone II (422 isolates) as reconstructed using Pasteur **(C)** and Oxford **(D)** MLST schemes. Numbers inside each circle indicate the ST types. Circle size is proportional to the number of isolates belonging to the same ST type. Colors in **(C,D)** represent sub-branches identified by eBURST using the Oxford MLST scheme.

**FIGURE 4:**
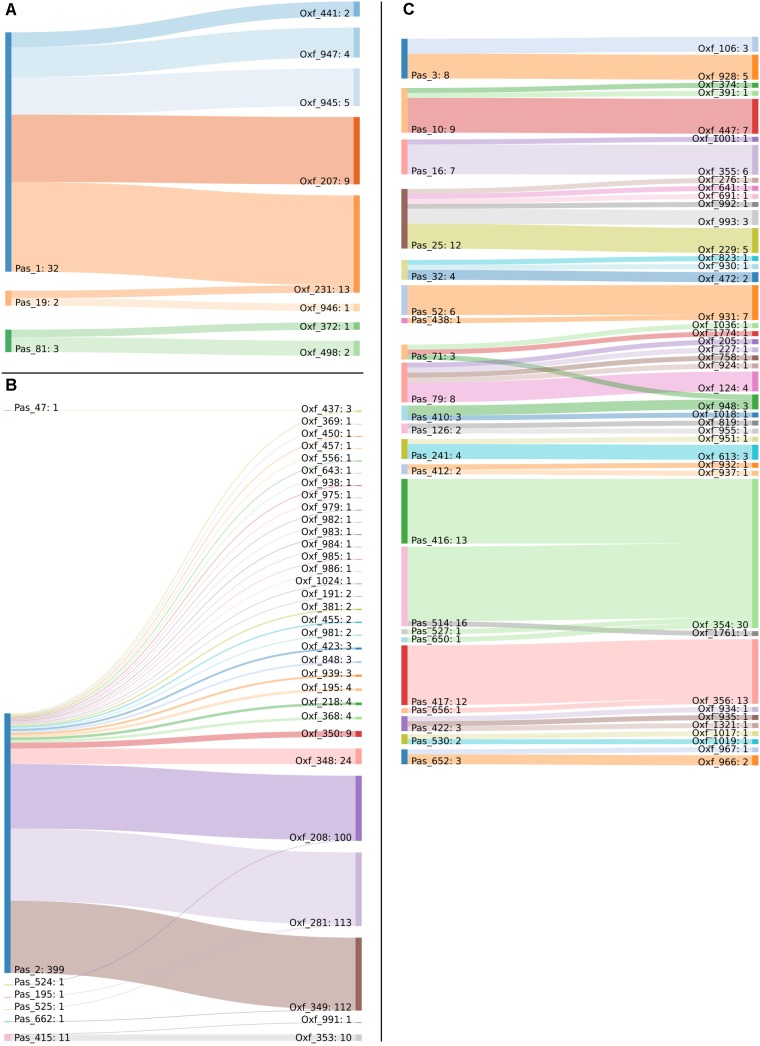
Sankey diagram of the MLST classification of the 730 genomes in use, as performed with the Pasteur and Oxford schemes. Two-way corresponding STs were removed to improve image clarity. Captions show the corresponding STs belonging to **(A)** International Clone 1, **(B)** International Clone 2, and **(C)** all the other genomes.

Minimum spanning tree analysis demonstrated that both MLST schemes gene sets discriminate the existing species within the Acb complex (Figure 3A,B, MLST-based species identification). ST assignments and eBURST analyses using our 730 genomes dataset generated a convenient and expandable table of correspondence between the two MLST schemes, represented in Table 2 and in Figure 4 and Supplementary Figure S2 as Sankey diagrams.

**Table 2:** Correspondence of *A. baumannii* clonal lineages as assessed by Oxford and Pasteur MLST schemes.

International clonal lineages^a^	Pasteur’s MLST^b^	Oxford’s MLST^b^	References^c^
I	CC1	CC231	Diancourt et al., 2010; Hamidian et al., 2017
II	CC2	CC208 CC281 CC349	Diancourt et al., 2010; Feng et al., 2016; Hamidian et al., 2017
III	ST3	CC928	van Dessel et al., 2004; Diancourt et al., 2010
	CC10	CC447	Zarrilli et al., 2013
	ST15	ST950	Di Popolo et al., 2011; Zarrilli et al., 2013
	ST25	CC229	Zarrilli et al., 2013; Sahl et al., 2015;
	ST32	ST172	Zarrilli et al., 2013; Da Silva et al., 2014
	CC52	ST931	Diancourt et al., 2010; Zarrilli et al., 2013
	ST78	ST944	Giannouli et al., 2010; Carretto et al., 2011; Zarrilli et al., 2013
	CC422	CC124	Grosso et al., 2011; Villalon et al., 2011; Zarrilli et al., 2013

*^a^ The International clonal lineages are identified by amplified fragment length polymorphism (AFLP) analysis. ^b^ Clonal complexes (CCs) are numbered according to the most prevalent clone. STs are indicated when singletons. ^c^ Key publications on each clone are reported.*

Then, we extracted CCs from both schemes and checked their monophyly on the core genome phylogeny. The Pasteur scheme had 12 CCs comprising a total of 47 STs, i.e., 35.34% of all STs. Additionally, there were 86 singleton Pasteur STs, while nine CCs are monophyletic. The Oxford scheme had 16 CCs comprising in total 82 STs, i.e., 44.56% of all STs. Additionally, there were 102 singleton Oxford STs and 11 monophyletic CCs. The Pasteur scheme appeared to be less discriminant, but more appropriate for precise strain classification into clonal groups. On the other hand, the Oxford scheme was able to identify additional genotypes and to differentiate isolates belonging to international clone II into three distinct clonal groups (Table 2 and Figure 3C,D).

The Gini-Simpson index of the 730 genomes was almost one order of magnitude higher when classifying the dataset using the Pasteur STs (0.70–0.93 for Oxford). When repeating the calculation on the genomes of the three International Clones, the score difference was lower but still important. The Pasteur scheme obtained values close to 0, being of low discrimination within the three ICs (Table 3).

**Table 3:** Gini-Simpson index values obtained using the STs of both schemes on the entire dataset of 730 genomes and separately on the three International Clones.

Scheme	Total	IC_I	IC_II	IC_III
Oxford	0.927543629	0.768518519	0.7799125	0.46875
Pasteur	0.695552637	0.203703704	0.0049875	0

### Topologies Comparison and Statistical Analysis

**FIGURE 5:**
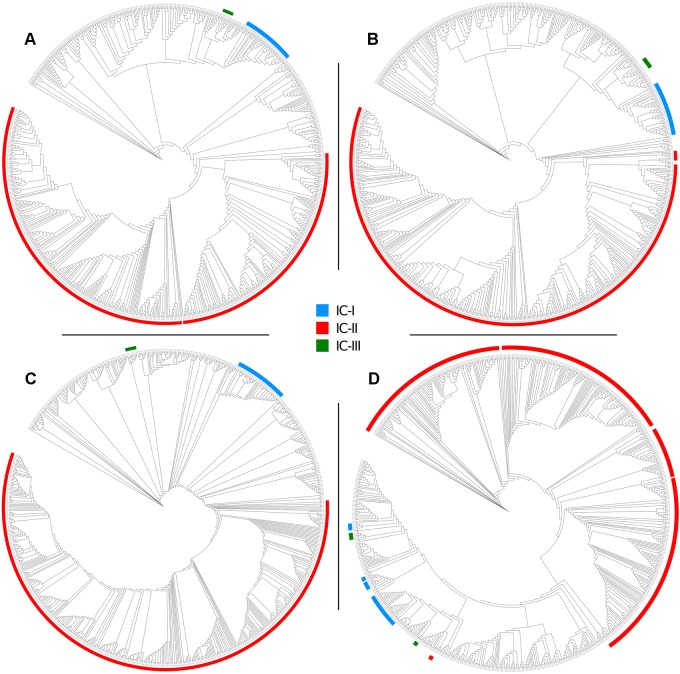
Maximum Likelihood phylogenies of 730 genomes of the *Acb* complex, inferred from **(A)** a concatenate of 1409 orthologous core genes, **(B)** a concatenate of 68,340 SNP positions, **(C)** a concatenate of the seven alleles used in the Pasteur MLST scheme, and **(D)** a concatenate of the seven alleles used in the Oxford MLST scheme. Major clonal complexes are highlighted: International clone I in blue, International clone II in red, International clone III in green.

To evaluate the two MLST schemes with respect to phylogenetic inference, we constructed four phylogenies (Figure 5): two using the concatenated alleles of the Pasteur and Oxford scheme as input, and two references using genome-wide data (a core-genome of 1409 high quality genes, and an alignment of 68,340 SNPs). We then statistically compared the trees to evaluate the reliability of MLST concatenates in relationship with the two references. All tests applied suggested a better congruence between the Oxford scheme and the references. Full results are reported in Table 4, which includes the results of cross check tests between the two reference phylogenies. In Figure 5, International Clones I, II, and III (calculated according to the Pasteur CC 1, 2, and 3) are highlighted in all four trees. In the tree obtained using the Oxford genes, the genomes of IC I are split in two separate, non-monophyletic, clades.

**Table 4:** Statistical comparison of the phylogenies obtained using the MLST loci of both schemes to the two reference trees obtained with genome-wide SNPS and core genes.

Reference tree	Tree	Matching clusters	R-F clusters	SH test		
				D (LH)	SD	*P*-value
Core genome	Oxford	17149	653	−560419,8575	4728,447118	<1%
Core genome	Pasteur	22348	669	−1114308,906	14114,36962	<1%
SNP	Oxford	15958	654	−226406,4429	2748,796098	<1%
SNP	Pasteur	22679	669	−576957,8653	10208,8312	<1%
SNP	Core genome	6197	497	−12777,39132	988,204341	<1%
Core genome	SNP	6197	497	−34788,30168	1285,842838	<1%

### Recombination Analysis

A recombination analysis was run on the alignments of all MLST gene fragments. All gene sequences presented signs of recombination in the *non-baumannii* genomes. This suggested that the analysis was biased by an uneven evolutionary distance and was repeated only on the 703 *A. baumannii* genomes. This step allowed detecting a recombination in the *gpi* locus of the Oxford scheme, while all other loci appeared to be recombination free. These data are in partial agreement with a previous study, which detected recombination in the topologies of the phylogenetic trees generated for the *gyrB* and *gpi* genes using the Oxford MLST scheme (Hamouda et al., 2010). High recombinogenicity of the *gpi* locus was also detected in other studies, which focused on the genomic plasticity of the capsular loci (Kenyon and Hall, 2013; Holt et al., 2016; Schultz et al., 2016; Hamidian et al., 2019). Accordingly, the recombining locus *gpi* happens to have the highest variability in alleles (Figure 1B). These results suggest that the Pasteur scheme allele diversification is less affected by homologous recombination. Accordingly, the recombining locus *gpi* happens to have the highest variability in alleles (Figure 1B).

### MLST Correlation With Genomic Distance

**FIGURE 6:**
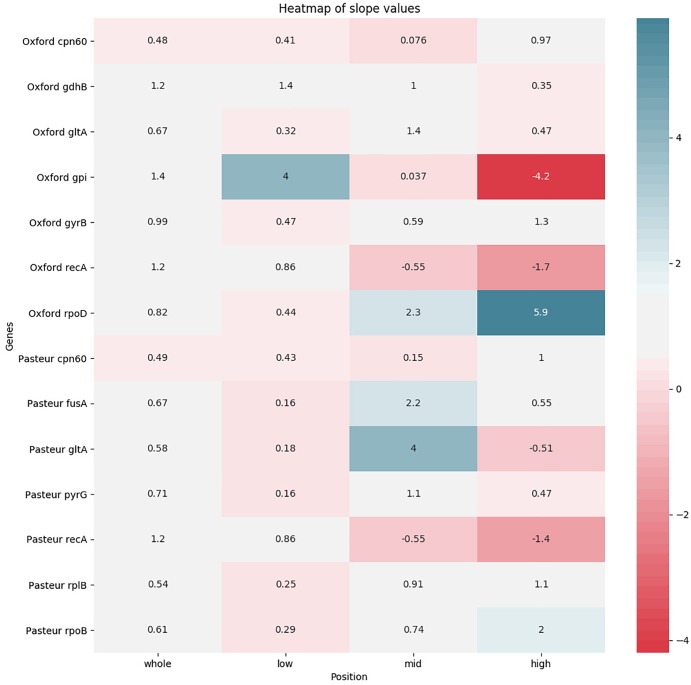
Heatmap showing the levels of agreement of each MLST locus to a reference alignment based on the core genome. Agreement levels are shown in terms of regression line slopes obtained from distance values between genome pairs. For each locus four regression were obtained, based, respectively, on the entirety of genome-wide distances and three subranges. White color indicates that a MLST locus represents the genome-wide distances well, showing a similar evolutionary pace. Red shades (indicating a negative correlation) and blue shades (indicating that the MLST locus shows an evolutionary pace much higher than the genome wide distances) indicate that the locus variation does not well represent genome variation.

The pairwise sequence distance between each MLST locus of each pair of genomes was plotted against the corresponding genome-wide distance in order to test its correlation. The test was repeated focusing on three different ranges of genome-wide distances (as in Bleidorn and Gerth, 2018). A total of 56 plots was obtained and reported in Supplementary Figure S3. We expected high quality genes to have a regression line with a positive slope (direct correlation between genomic and locus distance), as close as possible to a value of 1 (this means that the evolution of the MLST sequence follows the same pace as the genome). We thus summarized the slope data in two heatmaps: one with the slope values (Figure 6) and one with the *R*^2^-values (Supplementary Figure S4) to assess reliability of the regression. In general, both schemes perform better at lower genomic distances. This trend is highlighted especially in the *recA* gene, shared between the two schemes. Generally speaking, Pasteur alleles perform well when analyzing high genomic distances, while Oxford alleles have better scores at lower genomic distances. Finally, the *gpi* gene deserves a particular mention: at low genomic distances, the locus shows a quicker evolution than the genomic reference, while at high genomic distances, it shows an inverted evolution (i.e., at higher genomic distances correspond lower *gpi* distances). This could be probably due to the recombination detected in the gene sequence: at lower evolutionary distances, recombinations can increase variation, while they can act as equalizers at larger scales, having a cohesive effect (Doroghazi and Buckley, 2011).

## Discussion

The aim of this investigation was to compare the two MLST schemes that are widely used to genotype isolates of the Acb species complex. We decided to tackle the problem evaluating the two schemes, their reproducibility, discrimination, strain classification into CCs and compatibility among MLST-based phylogenies and genome-based phylogenies.

Starting from a curated dataset of 730 genomes, two phylogenomic trees were obtained from information collected throughout the whole genomes (core genes and core SNPs). The two resulting trees showed highly similar topologies and can be considered close approximations of the real evolutionary history of the Acb species complex. For this reason, they were used as reliable and unbiased references for the analyses. A phylogeny was obtained from the concatenate alignment of the alleles of each of the two MLST schemes and compared with the two references using three different statistical methods. In all three cases, the tree obtained from the Oxford scheme resulted in a closer approximation of the references.

On the other hand, previous publications described a series of limitations and issues of the Oxford scheme (Hamouda et al., 2010; Hamidian et al., 2017), such as the inclusion of the primers in the registered allele sequences of two of the seven MLST genes. This unusual procedure leads to replacing the true sequence with the primer sequence at these locations, creating mosaic sequences (primerF + internal sequence from isolate + primerR), and removing variation at priming sites when sequenced using primer-based methods. This issue was recognized and corrected previously, so it should not affect future identification, but it remains for all the previously investigated strains that were analyzed by PCR and not *in silico* (Hamidian et al., 2017).

Here, we detected an entirely novel problem with *in silico* determination of Oxford profiles, namely the presence of a paralog of the Oxford gene *gdhB* in a high proportion (553 out of the 730) of Acb genomes, a locus that we found to be often annotated as *gdhB2*, and that is located in a different genomic region. On multiple occasions, allele sequences resulting from this duplication were incorrectly used to establish new Oxford STs that do not actually exist, as they are based on alleles of the paralog *gdhB2*. This issue can be explained with an event of gene duplication, or of horizontal gene transfer, the second being more probable considering the low identity between the two paralogs (around 73%). Such event would have occurred early in the evolution of the Acb complex, possibly at the root of the *A. baumannii* species, followed by a quick sequence divergence of *gdhB2* and by the loss of this gene in a number of representatives of the Acb complex (177 out of 730 in our dataset). Nucleotide composition analysis shows that both *gdhB* and *gdhB2* have higher than average codon adaptation index, thus not allowing to understand which of the two events could be more likely.

The incorrect alleles are, to date (14 September 2018) 182 and 189 and have led to the determination of 30 STs: 1567, 1604, 1677, 1678, 1793, 1794, 1796, 1800, 1804, 1805, 1806, 1807, 1808, 1809, 1813, 1815, 1816, 1833, 1834, 1835, 1836, 1837, 1838, 1839, 1840, 1841, 1843, 1851, 1852, 1857. Other 38 unregistered 182-like alleles were found in the dataset used in this project and should not be registered if submitted to the MLST database. We suggest that these *gdhB2*-based alleles should be removed from the database, and each of the genomes belonging to these STs should be re-analyzed excluding the paralog *gdhB2* (a stringent allele calling filter on genetic similarity could be useful for this purpose) to find the correct *gdhB* allele and subsequently the current ST. The inclusion of the paralog allele in the database is due to the bioinformatics methods used, which did not take into account the possible presence of such paralog. This does not appear to have ever happened in PCR-based classification, as the *gdhB* primers are sufficiently specific to amplify only the correct locus. While this issue can be solved using bioinformatics, in silico MLST can be performed with different software tools including in-house scripts, so we cannot rule out the possibility of novel *gdhB2*-based alleles to appear in the future.

Another issue of the Oxford scheme, albeit one that mostly impact phylogenetic analyses, is the presence of possible recombinations, previously reported for two of the loci used, *gpi* and *gyrB* (Hamouda et al., 2010). Our analysis does not show a clear signal of recombination in the *gyrB* gene, but we detected a strong recombination signal in the *gpi* gene. The fact that recombination in the *gyrB* locus was detected previously but not within this study could be explained by the use of different methods, as Hamouda and colleagues used a general phylogenetic approach (Hamouda et al., 2010), while we chose an *ad hoc* recombination detection software.

Clearly, the most reliable classification method for *Acinetobacte*r isolates would be one based on genome-wide information, such as a core genome MLST (cgMLST) (Maiden et al., 2013). Whole genome sequencing, which is required to extract cgMLST data, is now a routine task in many research laboratories, with costs comparable to performing the seven PCR amplifications and Sanger sequencing required for the traditional MLST. Whole genome sequencing has been used to study *A. baumannii* phylogeny (Snitkin et al., 2011; Sahl et al., 2013; Chan et al., 2015; Wallace et al., 2016), but only two studies so far used cgMLST schemes for *A. baumannii* (Fitzpatrick et al., 2016; Higgins et al., 2017). The cgMLST scheme by Fitzpatrick and colleagues analyzed genetic similarity based on SNPs in the core genome of a limited number of Acb complex bloodstream isolates, 116 *A. baumannii*, 28 *A. pittii*, and 3 *A. nosocomialis*, and showed higher discriminatory power than PFGE and MLST (Fitzpatrick et al., 2016). Higgins et al. (2017) developed a cgMLST scheme based on 1,339 *A*. *baumannii* genomes and validated on 53 *A*. *baumannii* genomes. The cgMLST clustering showed a good correlation between PFGE types and also matched the classification of *A. baumannii* international clones as previously determined by DiversiLab typing or MLST (Higgins et al., 2017). The set of genes by Higgins works well when analyzing strains of *A. baumannii sensu stricto* but is not fit for the other species of the Acb complex (Higgins et al., 2017) and should thus be restricted to shared genes in order to allow broader use.

As NGS is not yet accessible in all diagnostic laboratories in the world, cgMLST is probably still unfit to be a globally shared typing technique. Additionally, cgMLST classification could in some cases be incompatible with previous works that used MLST classifications, especially if specific STs are found to be polyphyletic and considering that determining STs *in silico* must be done with caution, as highlighted by our discovery of incorrect *gdhB* alleles identified based on a paralog sequence. Therefore, 7-gene MLST is likely to continue being used widely in the near future.

## Conclusion

In conclusion, the two MLST schemes have complementary characteristics, each with their own advantages: the Pasteur scheme shows lower discrimination, is able to better identify clonal lineages, and in general performs better when comparing evolutionary distant clones. The Oxford scheme in turn shows higher concordance with phylogenies and works better for discrimination among strains at short evolutionary distances. However, a novel and important issue of the Oxford scheme in the genomic era is the presence of an alternative *gdhB* locus in the majority (533/730) of the *A. baumannii* genomes. Besides, the presence of primers in sequence templates of two of the genes may have resulted in a few artefactual allele calls.

Previous works recommended the Oxford scheme due to the presence of the *gpi* gene, which is part of the capsular locus, and thus can provide a link between typing and phenotypic information (Kenyon and Hall, 2013; Holt et al., 2016). Other authors criticize both schemes due to the low level of resolution or polyphyly of Sts (Castillo-Ramírez and Graña-Miraglia, 2019). An opposite view could recommend the use of both schemes to provide a finer characterization. On the other hand, our opinion is to recommend the Pasteur scheme because of the following reasoning: both the link to phenotypic information and the finer characterization will be soon accomplished by the blooming cgMLST method, and will thus not be required for classical MLST schemes in the future. An MLST scheme in the genome era, however, retains great importance as a fundamental nomenclature tool. As such, absence of recombination, absence of wrongly called variants, and better adherence to the main epidemiological clones should be considered the main reasons to choose one of the two available schemes.

To summarize, we recommend the utilization of the Pasteur scheme for 7-gene MLST classification of *Acinetobacter* isolates of the Acb complex, and that future cgMLST nomenclature of genotypic groups should inherit, as much as possible, the Pasteur MLST denominations that were themselves inherited from pre-MLST international clone nomenclatures.

## Author Contributions

SG, SB, DS, and RZ designed the project. EE, KJ, and RZ curated the database and performed eburst analyses. SG and GB performed the bulk of the bioinformatic analyses. MC performed the phylogenetic analyses. SG, KJ, SB, DS, and RZ wrote the manuscript. All authors read and approved the final manuscript.

## Conflict of Interest Statement

SB was one of the developers of the Pasteur MLST scheme for *Acinetobacter* strains of the Acb complex. The remaining authors declare that the research was conducted in the absence of any commercial or financial relationships that could be construed as a potential conflict of interest.

## Footnotes

1 https://pubmlst.org/abaumannii/

## Supplementary Material

FIGURE S1 **(A)** Geographic and **(B)** temporal characterization of the database of 730 genomes used in the present work.

FIGURE S2 Complete Sankey diagram of the MLST classification of the 730 genomes in use, as performed with the Pasteur and Oxford schemes.

FIGURE S3 Plots showing the correlation of genetic distances of *Acinetobacter* genomes between MLST loci and genome-wide distances. Each data-point corresponds to a single pair of *Acinetobacter* genomes, and shows the divergence of each locus related to core-genome-based distance. For each locus, we show the full range of genome-wide distances and three different subregions representing different genomic distances (first block: 0.0–0.05, second block: 0.05–0.1, third: > 0.1).

FIGURE S4 Heatmap representing the correlation of each MLST locus in comparison to a reference alignment based on the core genome. Four plots were produced for each locus, based on genome-wide distances (see Supplementary Figure 3). In this figure, we report the *R*^2^-values of the interpolation lines.
