# Supplementary figures and images for: Comparative Analysis of the Two Acinetobacter baumannii Multilocus Sequence Typing (MLST) Schemes
Source: Front Microbiol. 2019 May 3;10:930. doi: 10.3389/fmicb.2019.00930 (PMC6510311; doi:10.3389/fmicb.2019.00930)

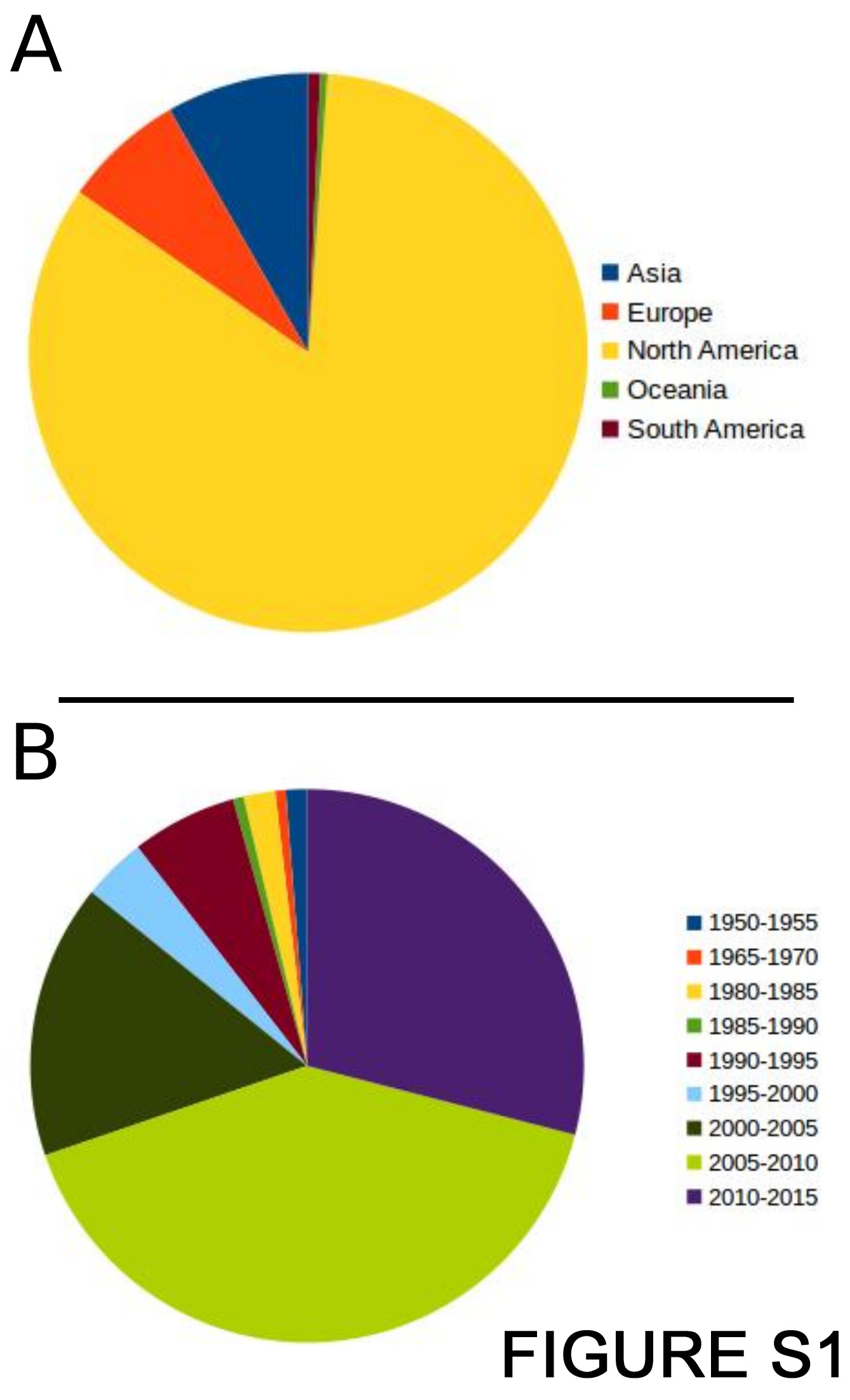

Supplement: FIGURE S1 — (A) Geographic and (B) temporal characterization of the database of 730 genomes used in the present work. [file Image_1.jpg]

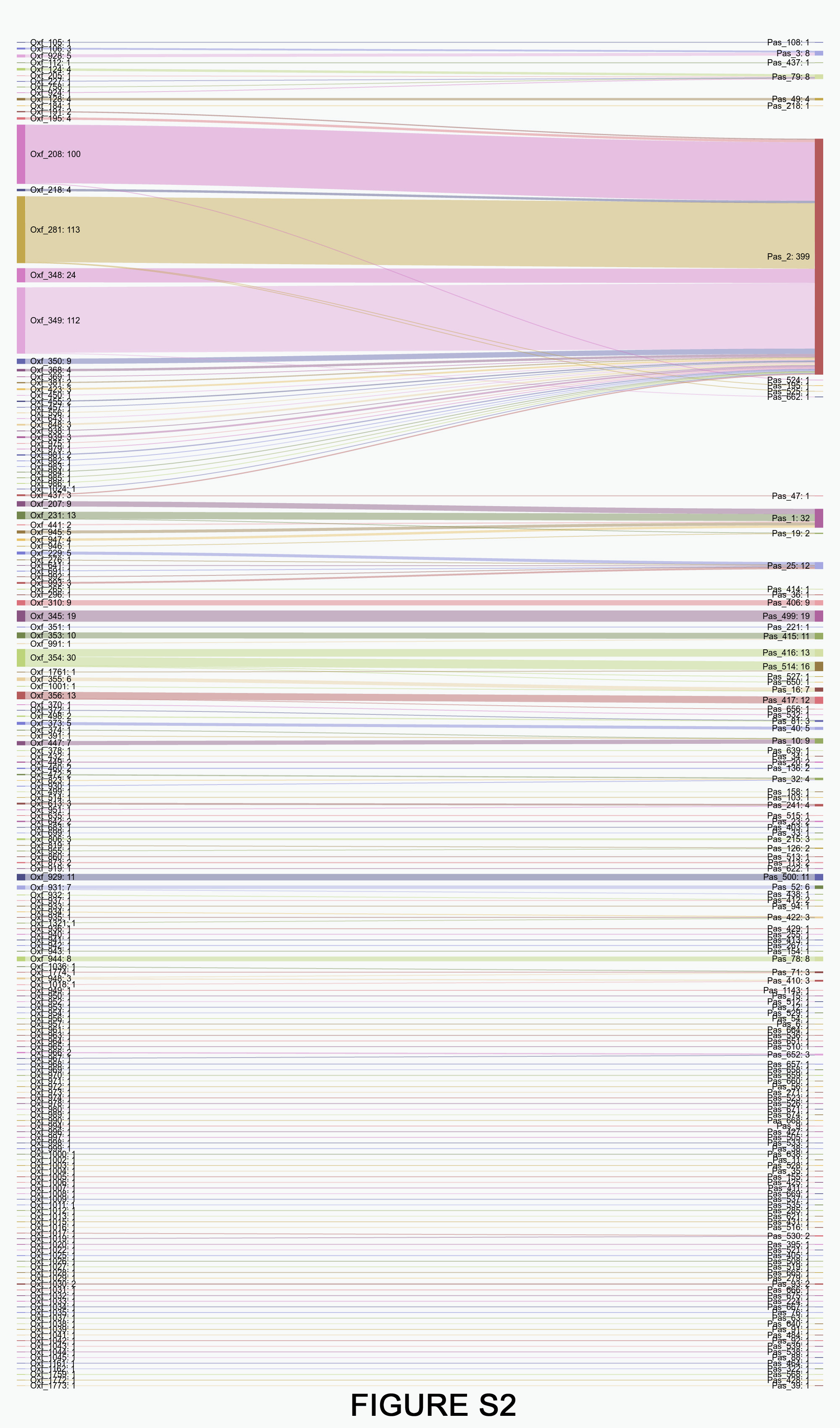

Supplement: FIGURE S2 — Complete Sankey diagram of the MLST classification of the 730 genomes in use, as performed with the Pasteur and Oxford schemes. [file Image_2.jpg]

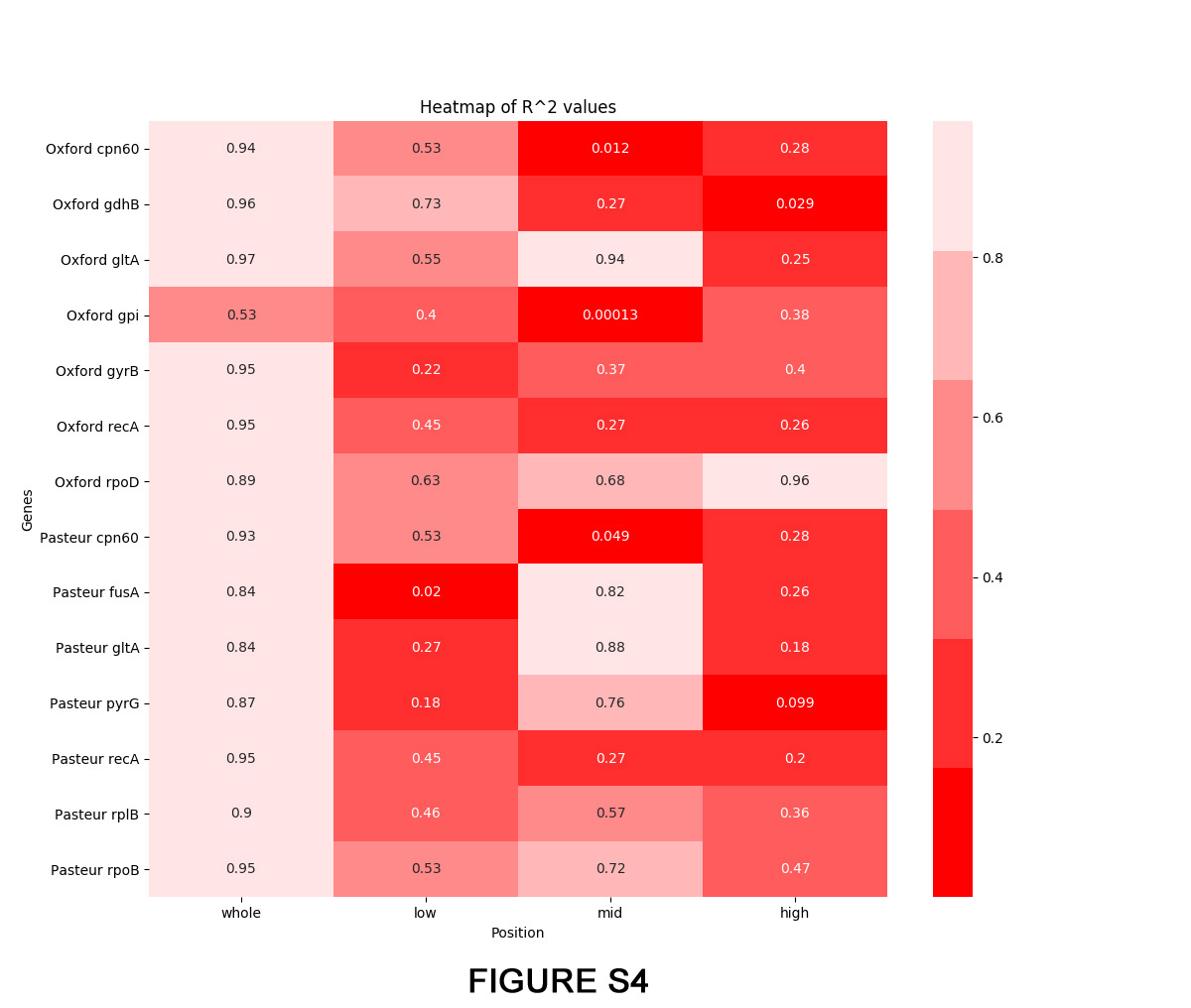

Supplement: FIGURE S4 — Heatmap representing the correlation of each MLST locus in comparison to a reference alignment based on the core genome. Four plots were produced for each locus, based on genome-wide distances (see Supplementary Figure 3). In this figure, we report the R2-values of the interpolation lines. [file Image_4.jpg]
