# Supplementary material for: Comparative Analysis of the Two Acinetobacter baumannii Multilocus Sequence Typing (MLST) Schemes
Source: Front Microbiol. 2019 May 3;10:930. doi: 10.3389/fmicb.2019.00930 (PMC6510311; doi:10.3389/fmicb.2019.00930)
Supplement: FIGURE S3 — Plots showing the correlation of genetic distances of Acinetobacter genomes between MLST loci and genome-wide distances. Each data-point corresponds to a single pair of Acinetobacter genomes, and shows the divergence of each locus related to core-genome-based distance. For each locus, we show the full range of genome-wide distances and three different subregions representing different genomic distances (first block: 0.0–0.05, second block: 0.05–0.1, third: > 0.1). [file Image_3.pdf]

Figure S3

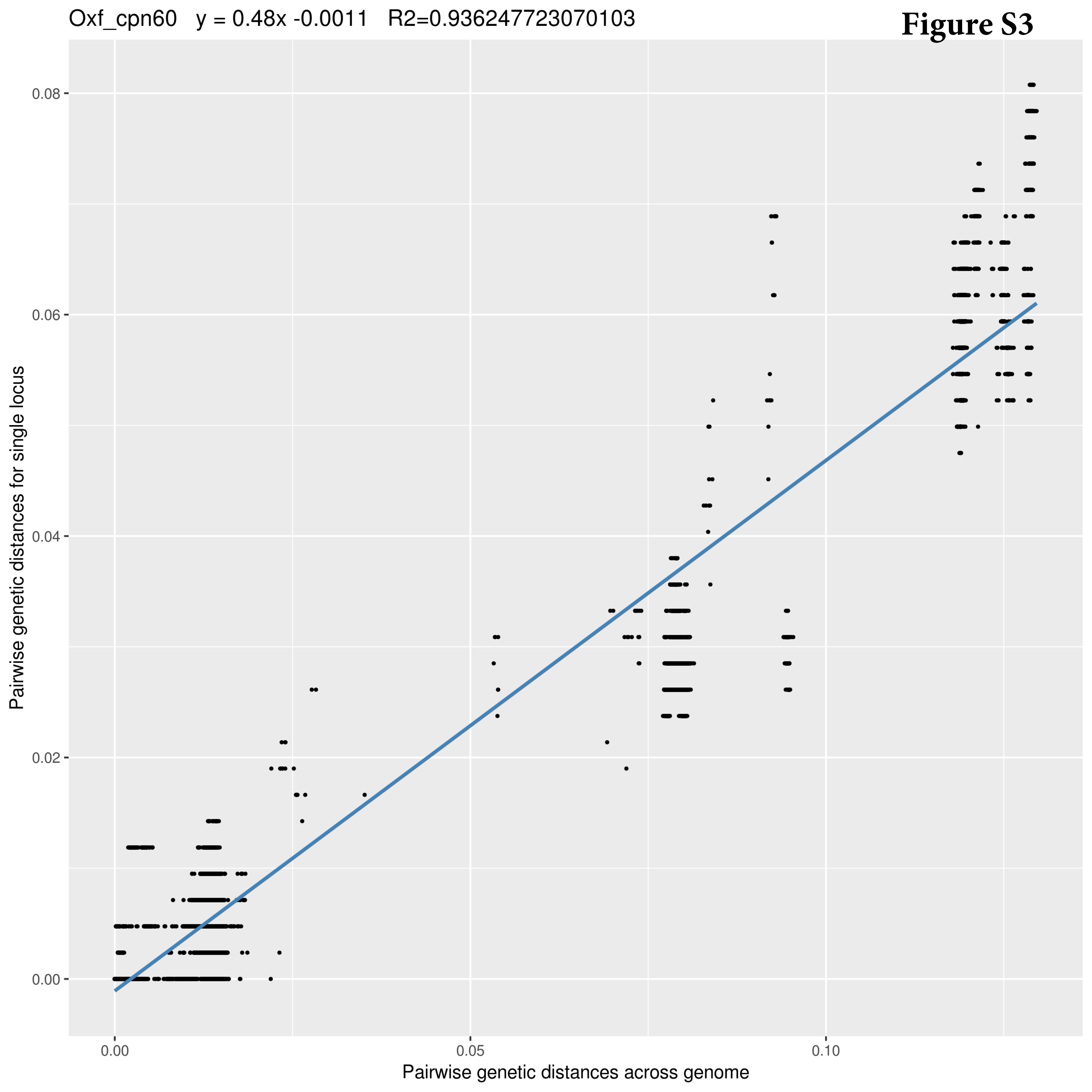
Oxf_cpn60   y = 0.48x -0.0011   R2=0.936247723070103
0.08
0.06
0.04
0.02
0.00
0.00
0.05
0.10
Pairwise genetic distances for single locus
Pairwise genetic distances across genome

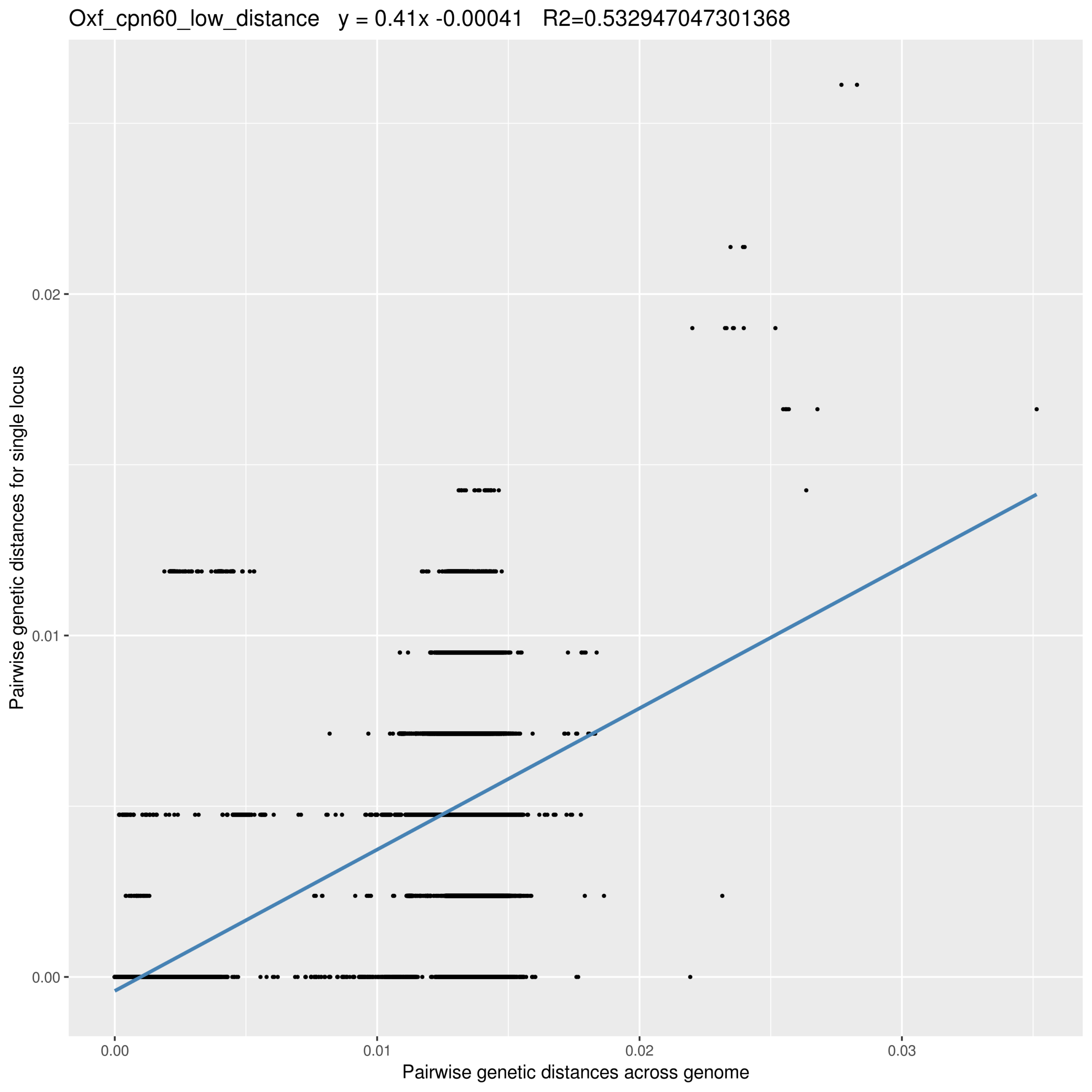

Oxf_cpn60_low_distance y = 0.41x -0.00041 R2=0.532947047301368
Pairwise genetic distances for single locus
0.02
0.01
0.00
0.00
0.01
0.02
0.03
Pairwise genetic distances across genome

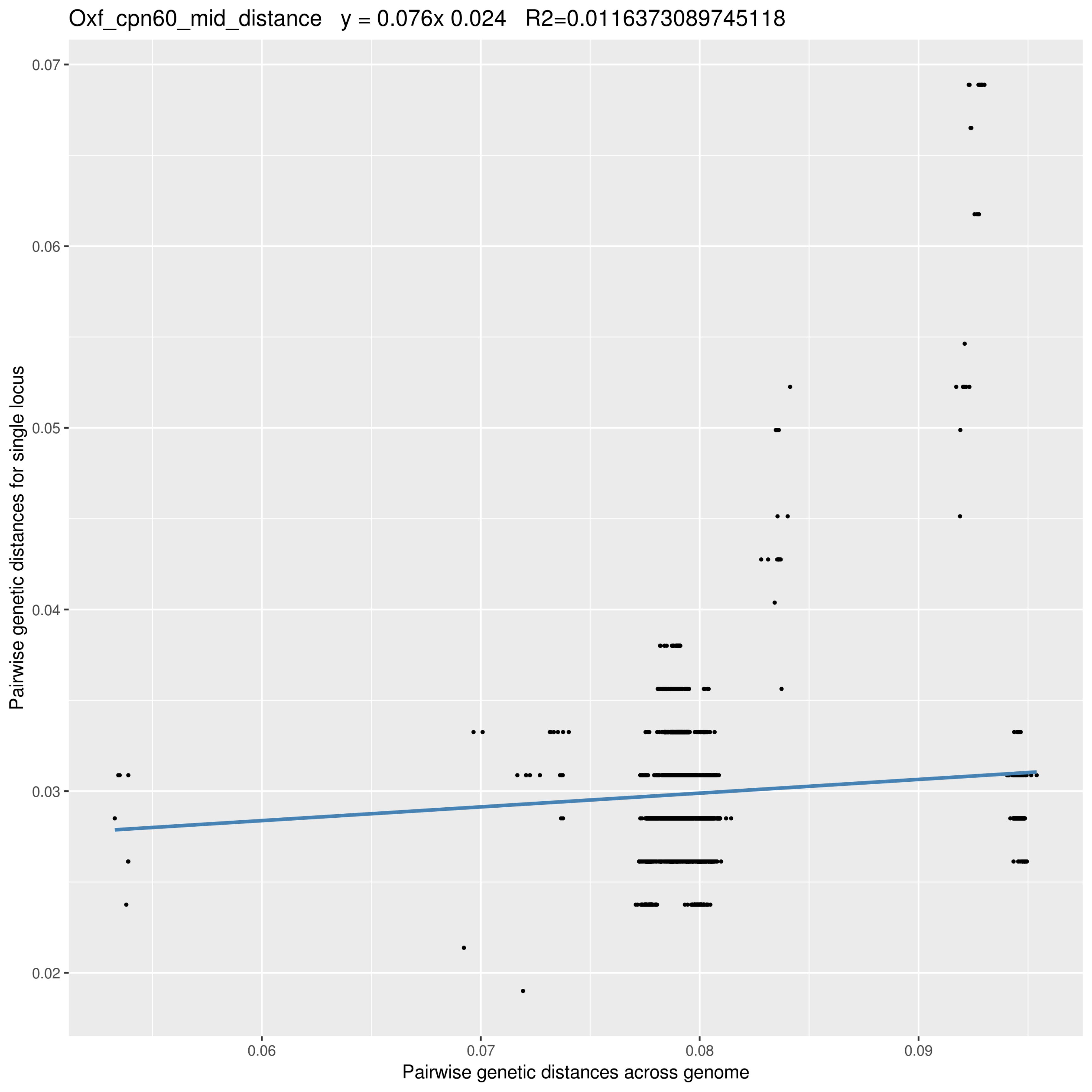

Oxf_cpn60_mid_distance y = 0.076x 0.024 R2=0.0116373089745118
Pairwise genetic distances for single locus
0.07
0.06
0.05
0.04
0.03
0.02
0.06
0.07
0.08
0.09
Pairwise genetic distances across genome

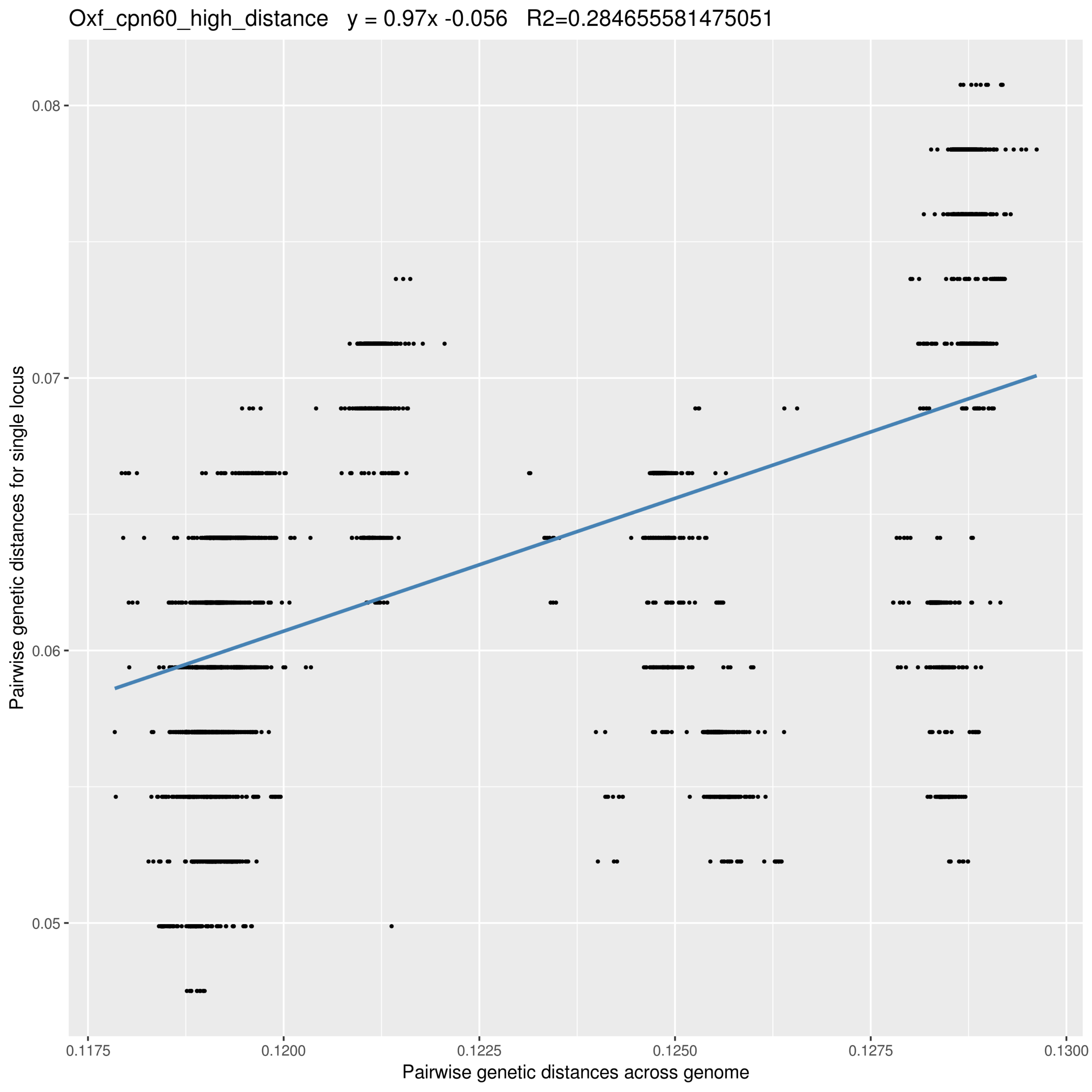

Oxf_cpn60_high_distance y = 0.97x -0.056 R2=0.284655581475051
Pairwise genetic distances for single locus
0.08
0.07
0.06
0.05
0.1175
0.1200
0.1225
0.1250
0.1275
0.1300
Pairwise genetic distances across genome

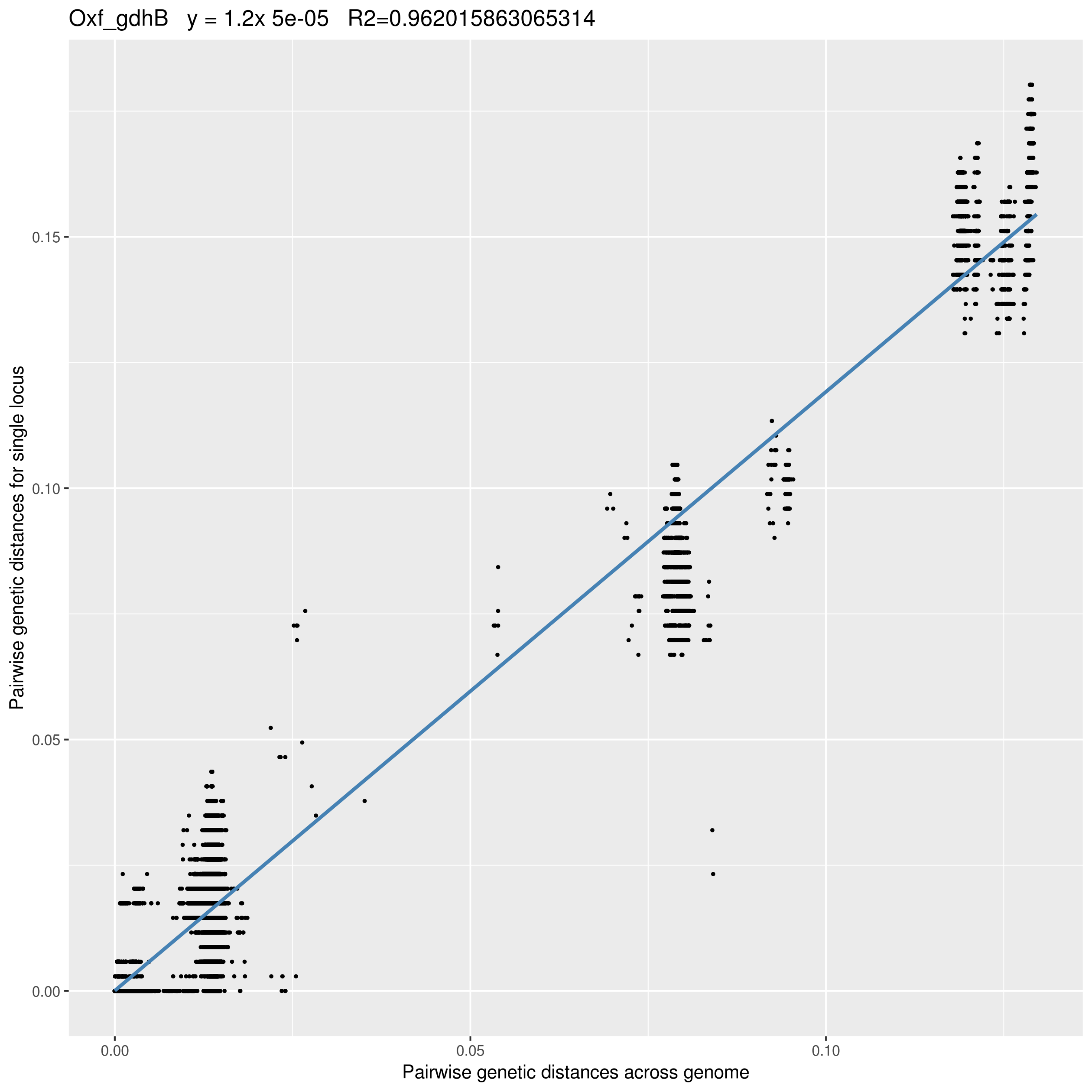

Oxf_gdhB y = 1.2x 5e-05 R2=0.962015863065314
Pairwise genetic distances for single locus
Pairwise genetic distances across genome
0.00
0.05
0.10
0.15

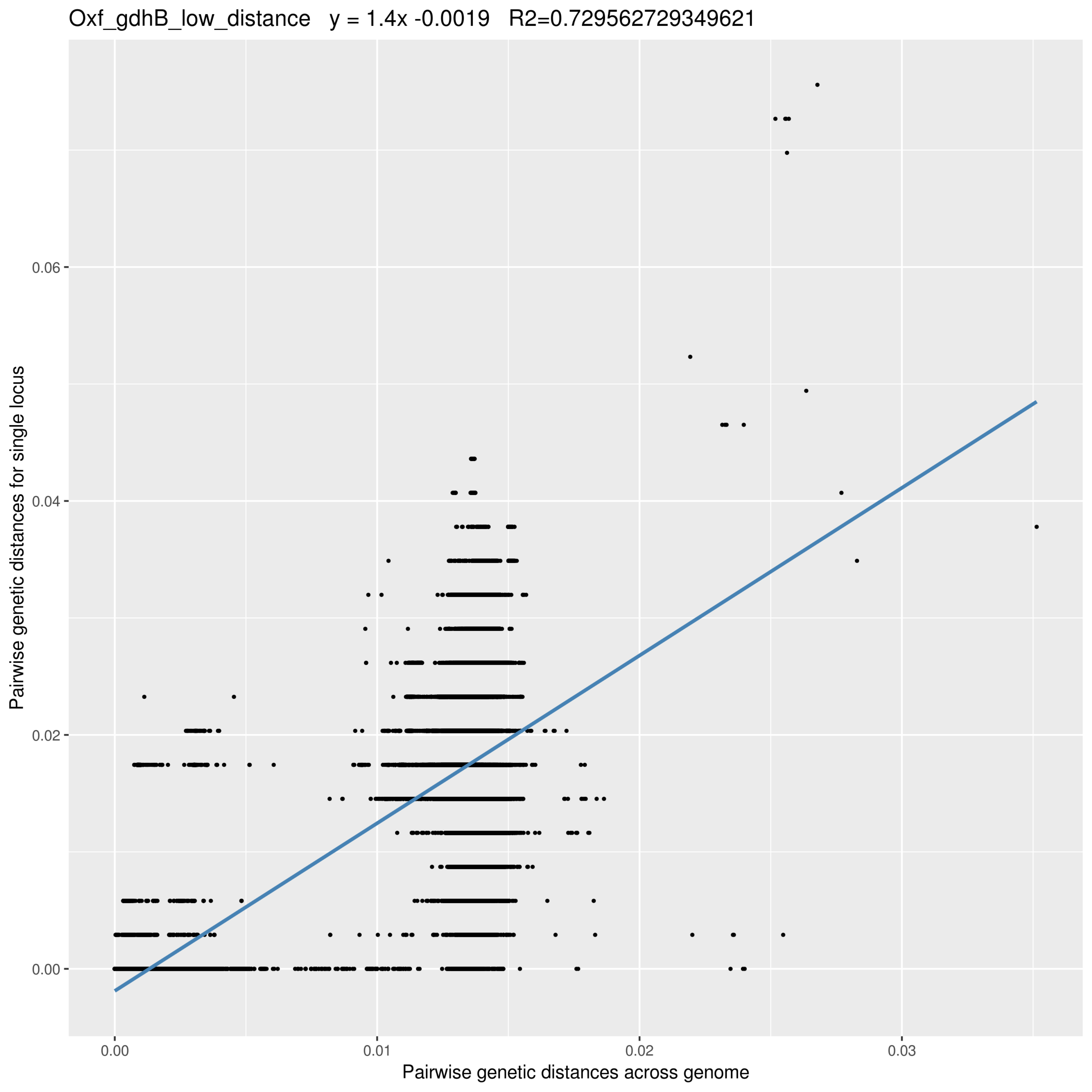
Oxf_gdhB_low_distance y = 1.4x -0.0019 R2=0.729562729349621
Pairwise genetic distances for single locus
0.06
0.04
0.02
0.00
0.00
0.01
0.02
0.03
Pairwise genetic distances across genome

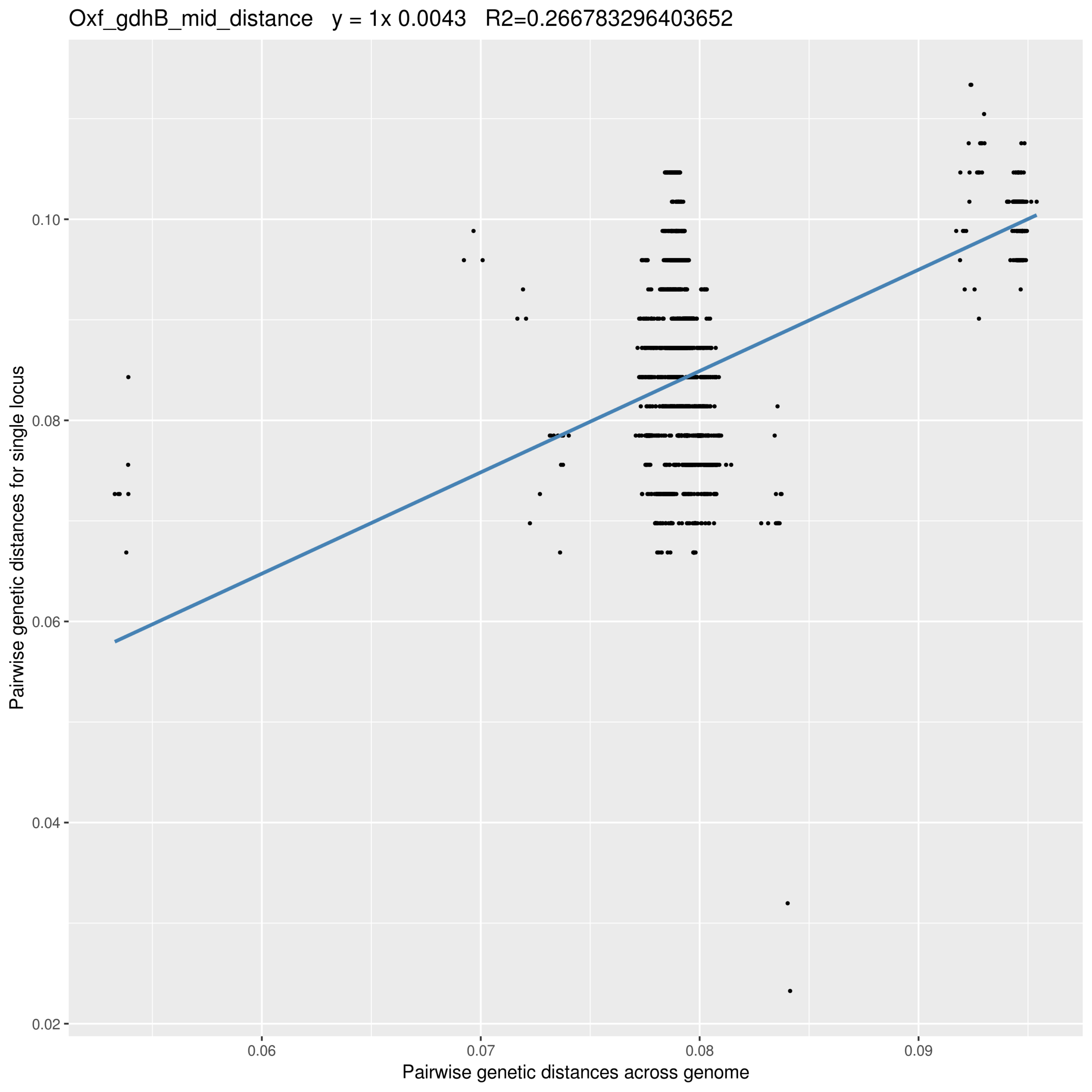
Oxf_gdhB_mid_distance   y = 1x 0.0043   R2=0.266783296403652
Pairwise genetic distances for single locus
0.10
0.08
0.06
0.04
0.02
0.06
0.07
0.08
0.09
Pairwise genetic distances across genome

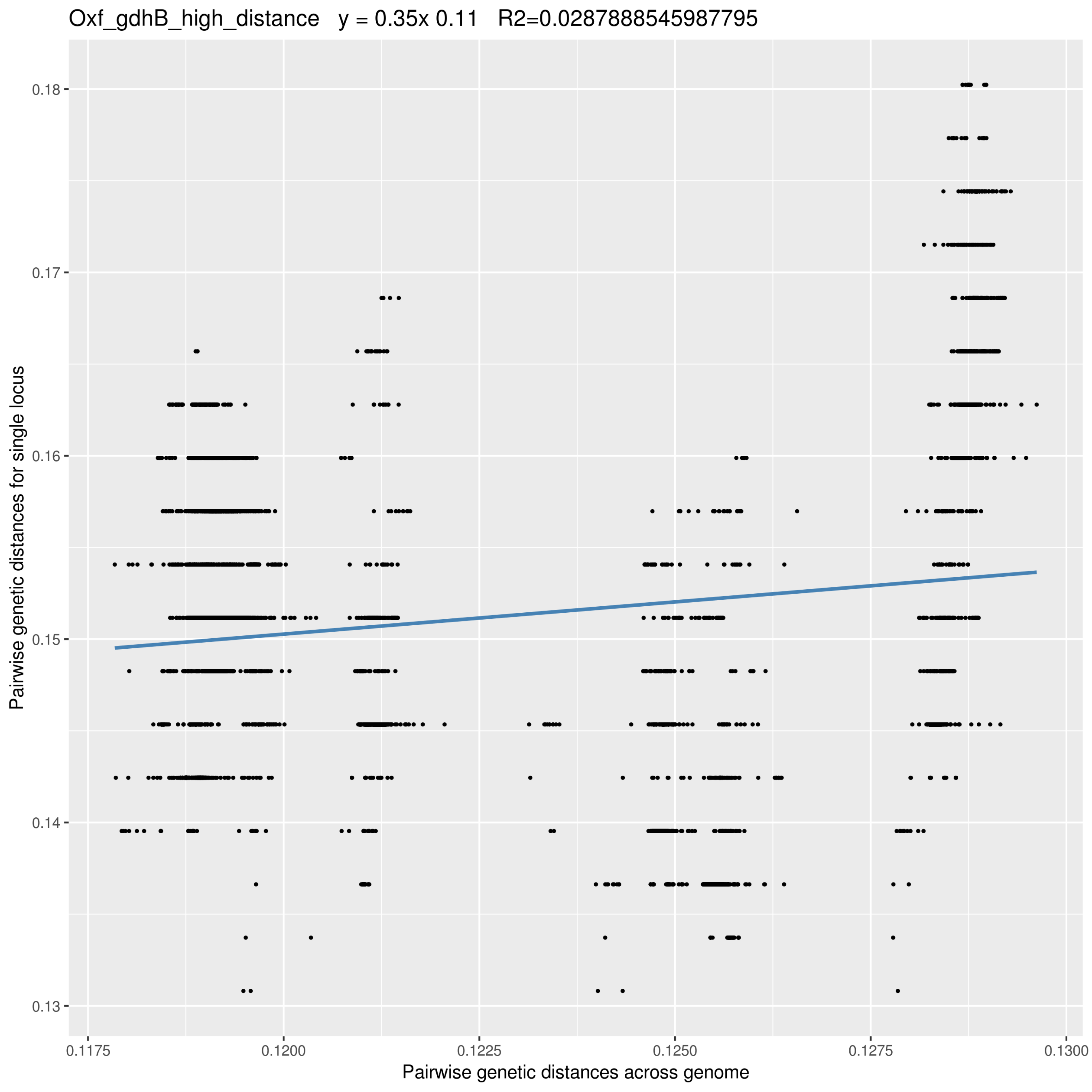
Oxf_gdhB_high_distance y = 0.35x 0.11 R2=0.0287888545987795
Pairwise genetic distances for single locus
0.18
0.17
0.16
0.15
0.14
0.13
0.1175
0.1200
0.1225
0.1250
0.1275
0.1300
Pairwise genetic distances across genome

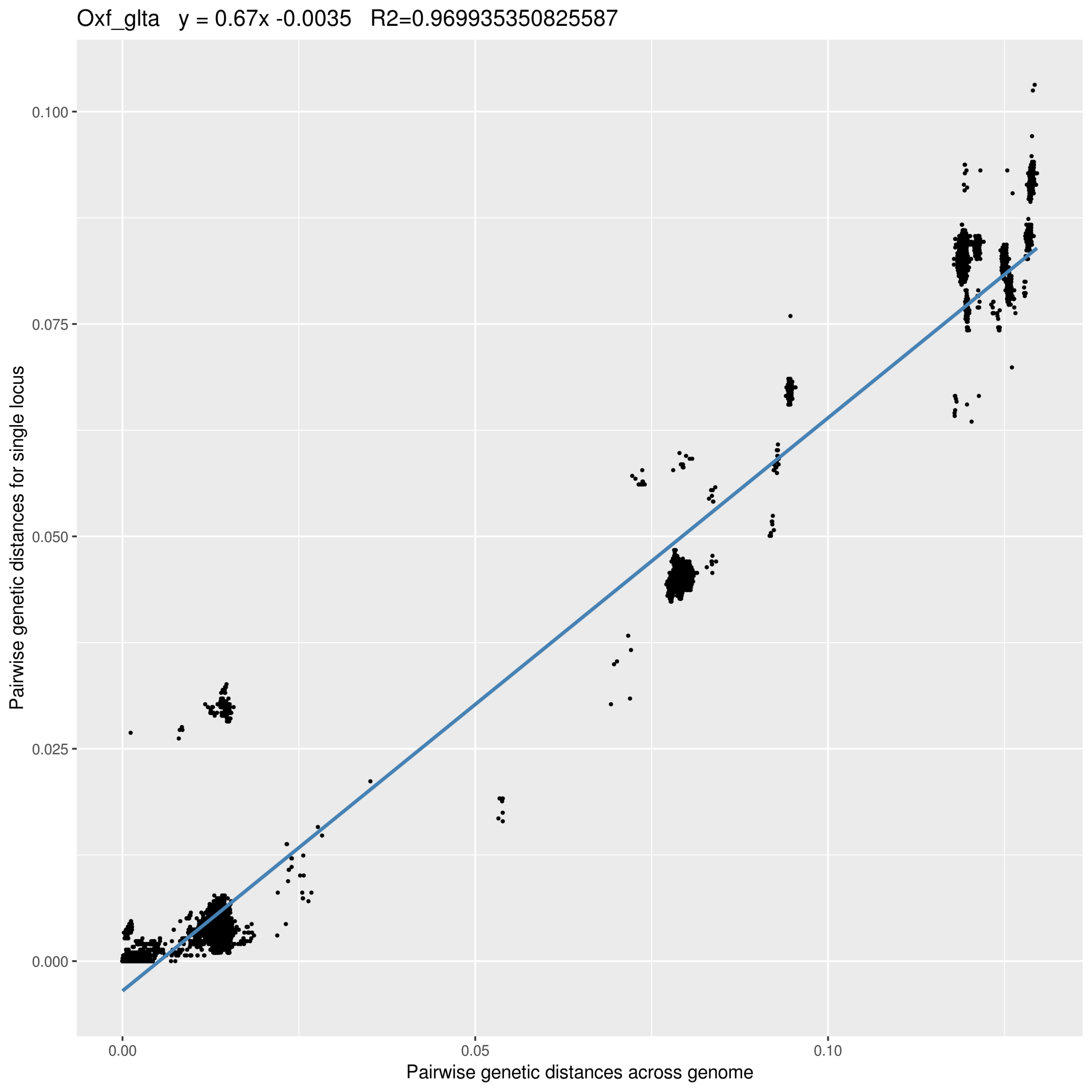

Oxf_glta   y = 0.67x -0.0035   R2=0.969935350825587
Pairwise genetic distances for single locus
0.100
0.075
0.050
0.025
0.000
0.00
0.05
0.10
Pairwise genetic distances across genome

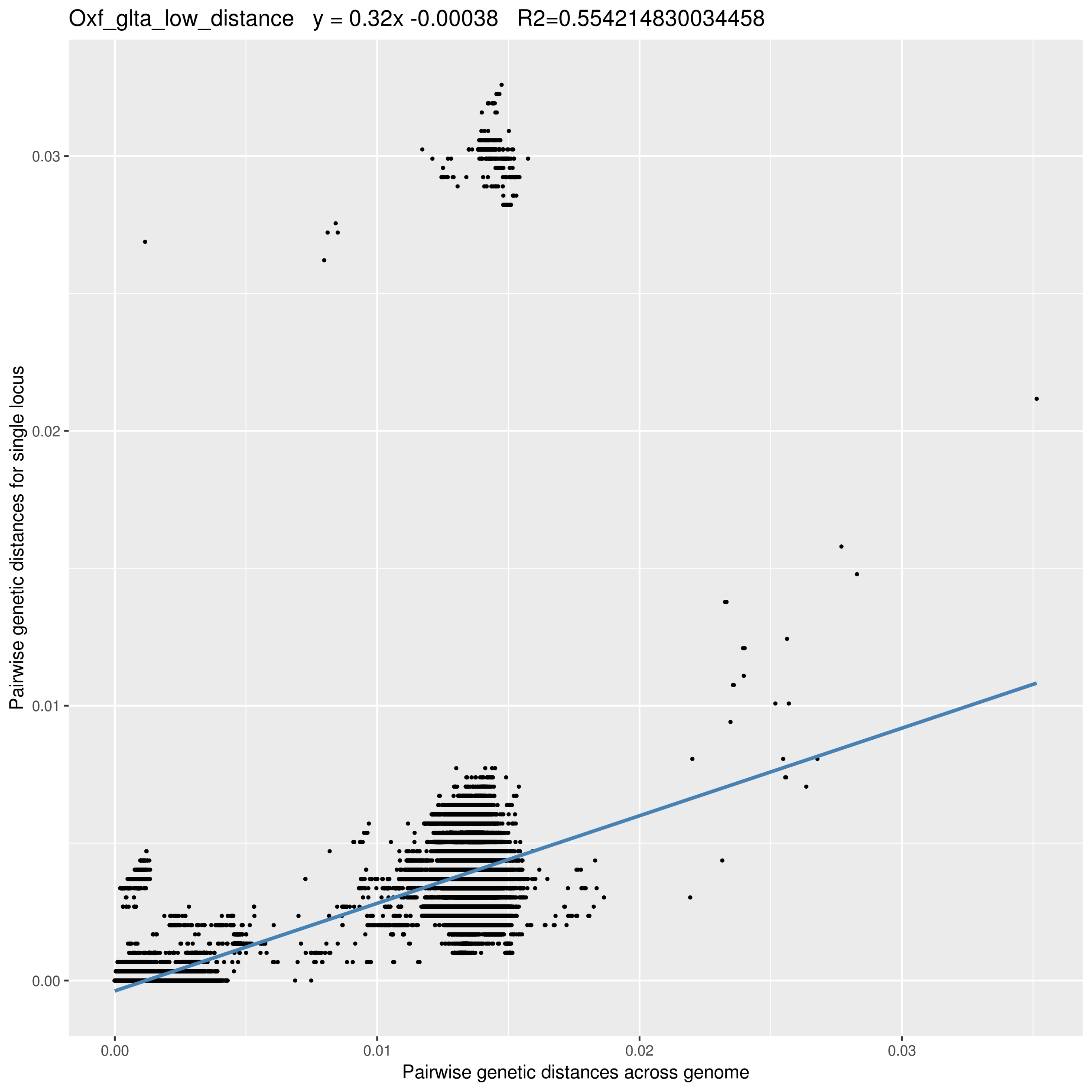
Oxf_glta_low_distance y = 0.32x -0.00038 R2=0.554214830034458
Pairwise genetic distances for single locus
0.00
0.01
0.02
0.03
0.00
0.01
0.02
0.03
Pairwise genetic distances across genome

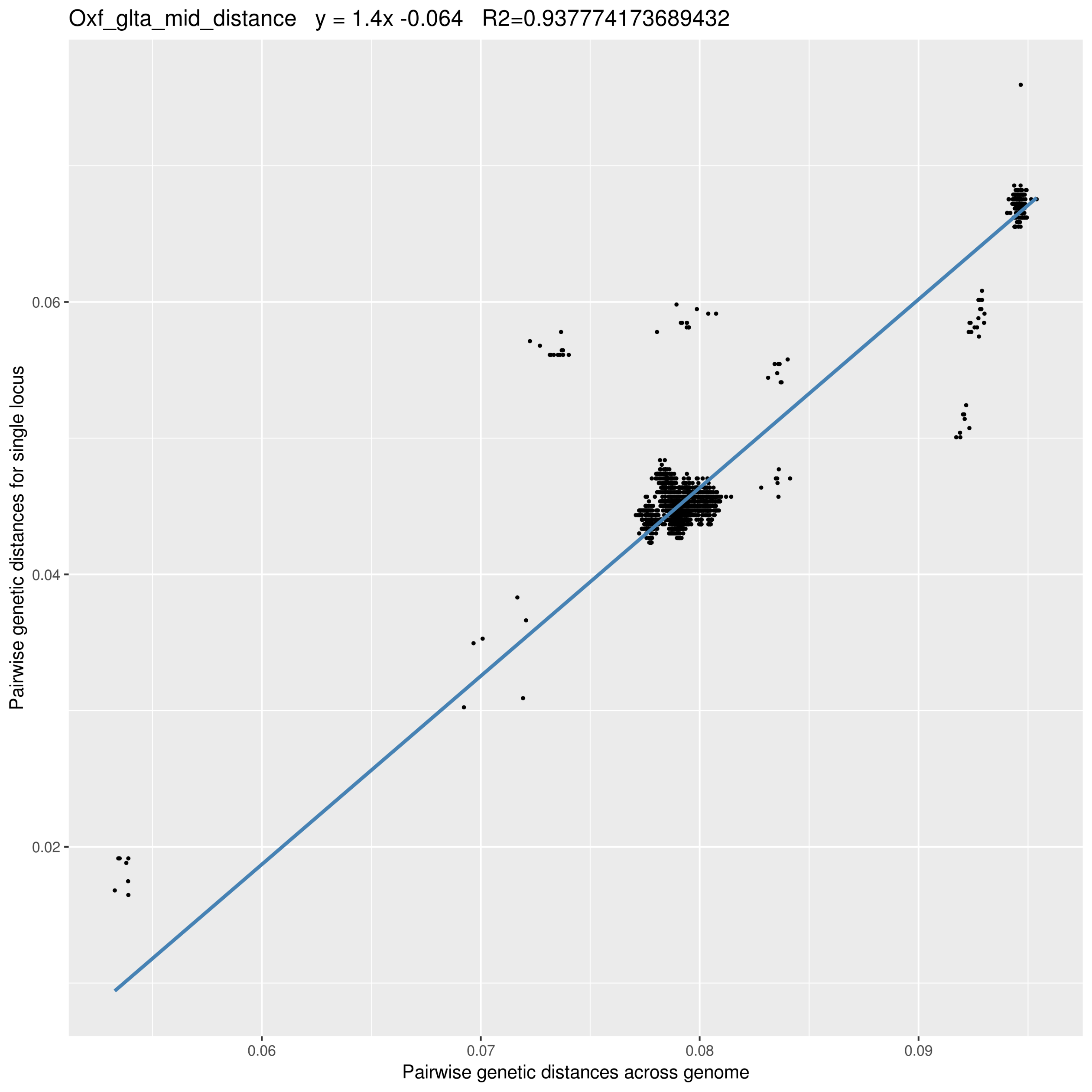

Oxf_glta_mid_distance   y = 1.4x -0.064   R2=0.937774173689432
Pairwise genetic distances for single locus
0.06
0.04
0.02
0.06
0.07
0.08
0.09
Pairwise genetic distances across genome

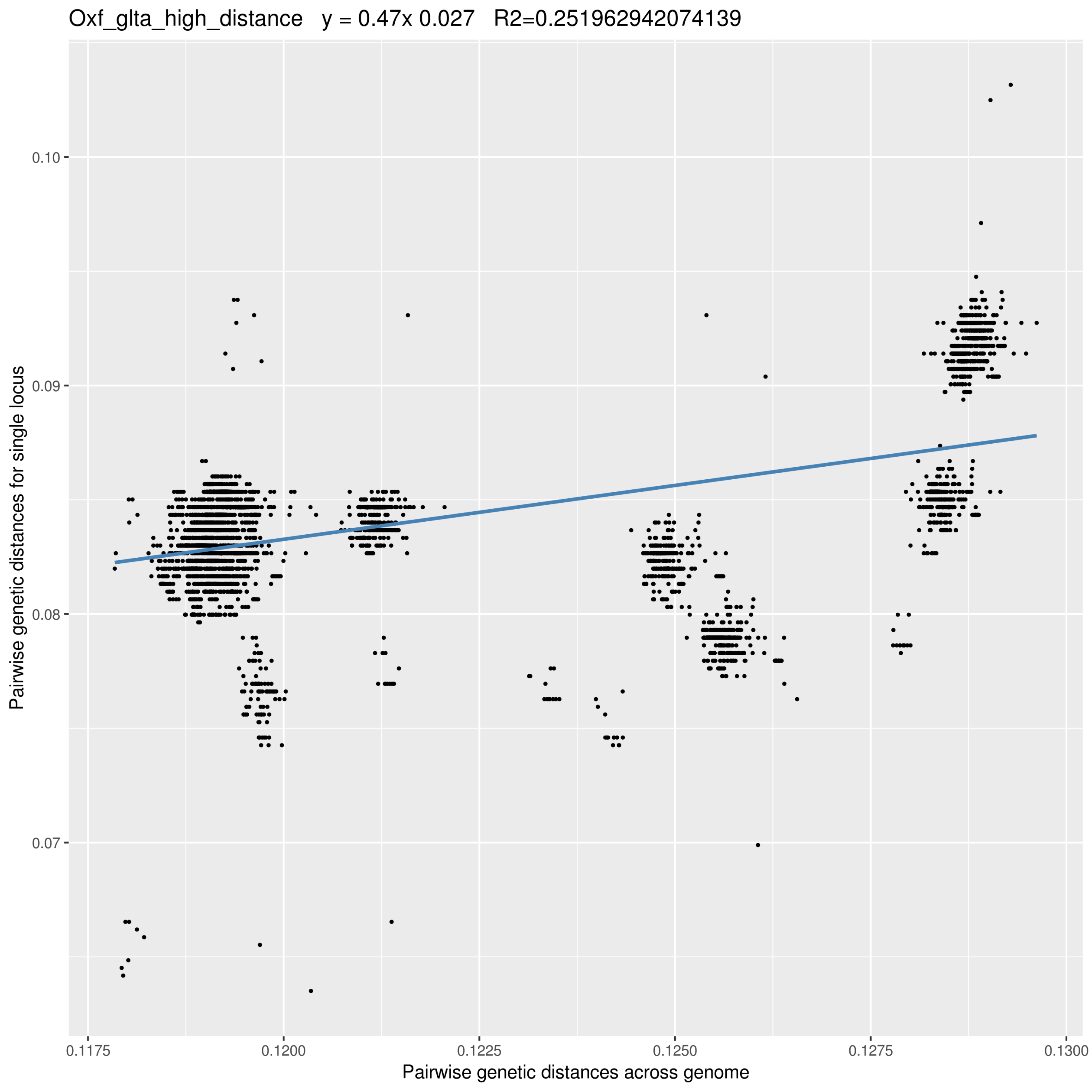
Oxf_glta_high_distance y = 0.47x 0.027 R2=0.251962942074139
Pairwise genetic distances for single locus
0.10
0.09
0.08
0.07
0.1175
0.1200
0.1225
0.1250
0.1275
0.1300
Pairwise genetic distances across genome

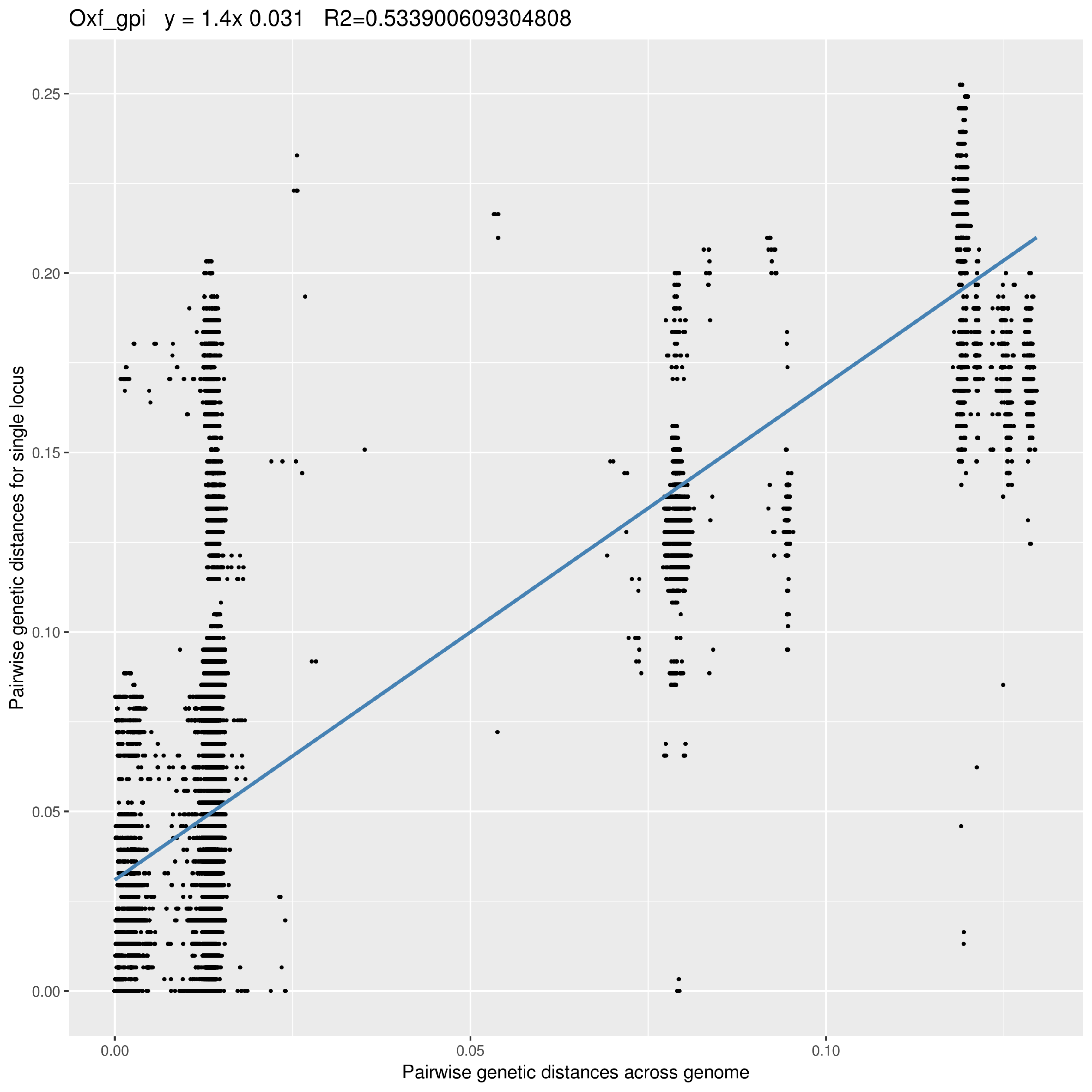

Oxf_gpi y = 1.4x 0.031 R2=0.533900609304808
0.25
0.20
0.15
0.10
0.05
0.00
Pairwise genetic distances for single locus
0.00
0.05
0.10
Pairwise genetic distances across genome

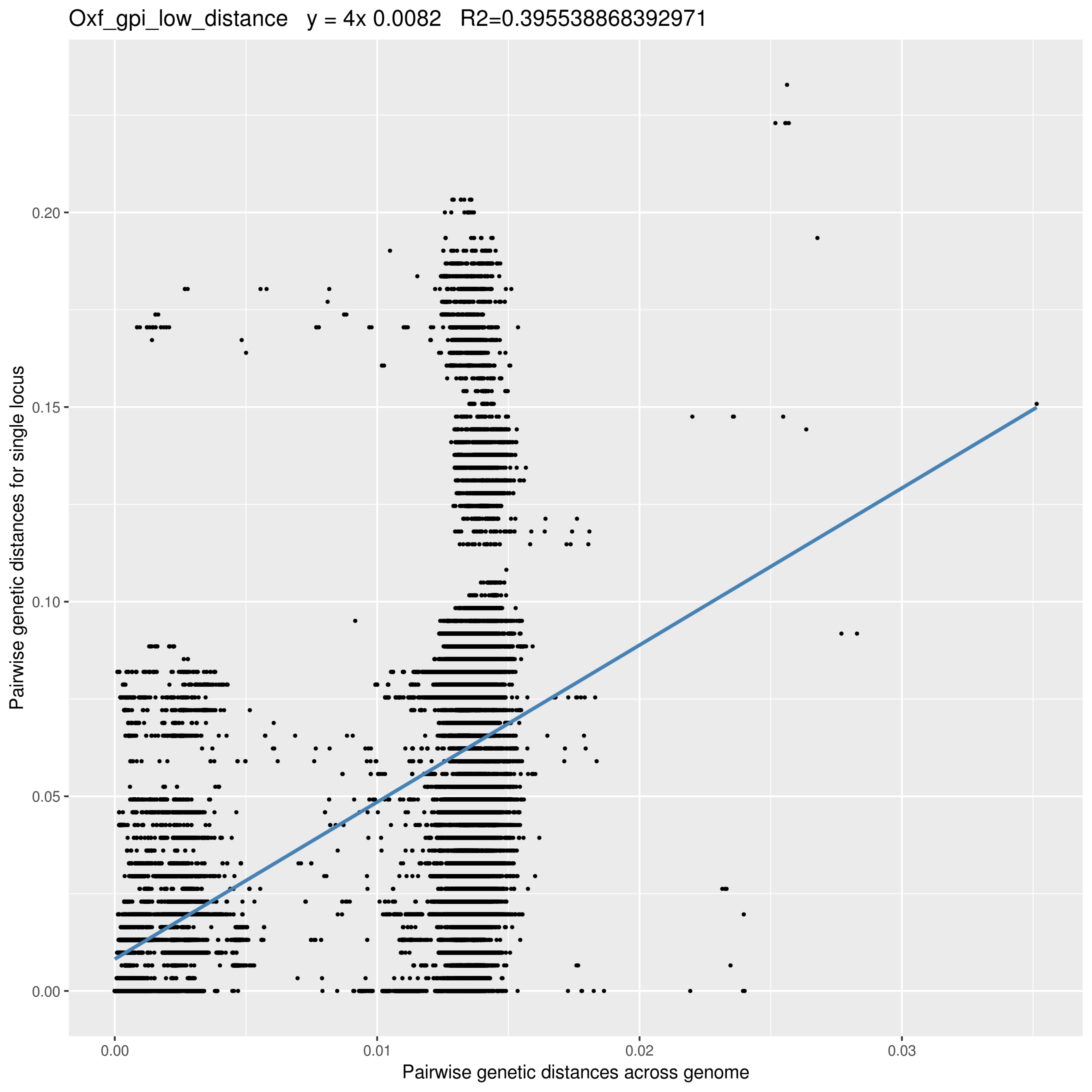
Oxf_gpi_low_distance   y = 4x 0.0082   R2=0.395538868392971
Pairwise genetic distances for single locus
0.00
0.05
0.10
0.15
0.20
0.00
0.01
0.02
0.03
Pairwise genetic distances across genome

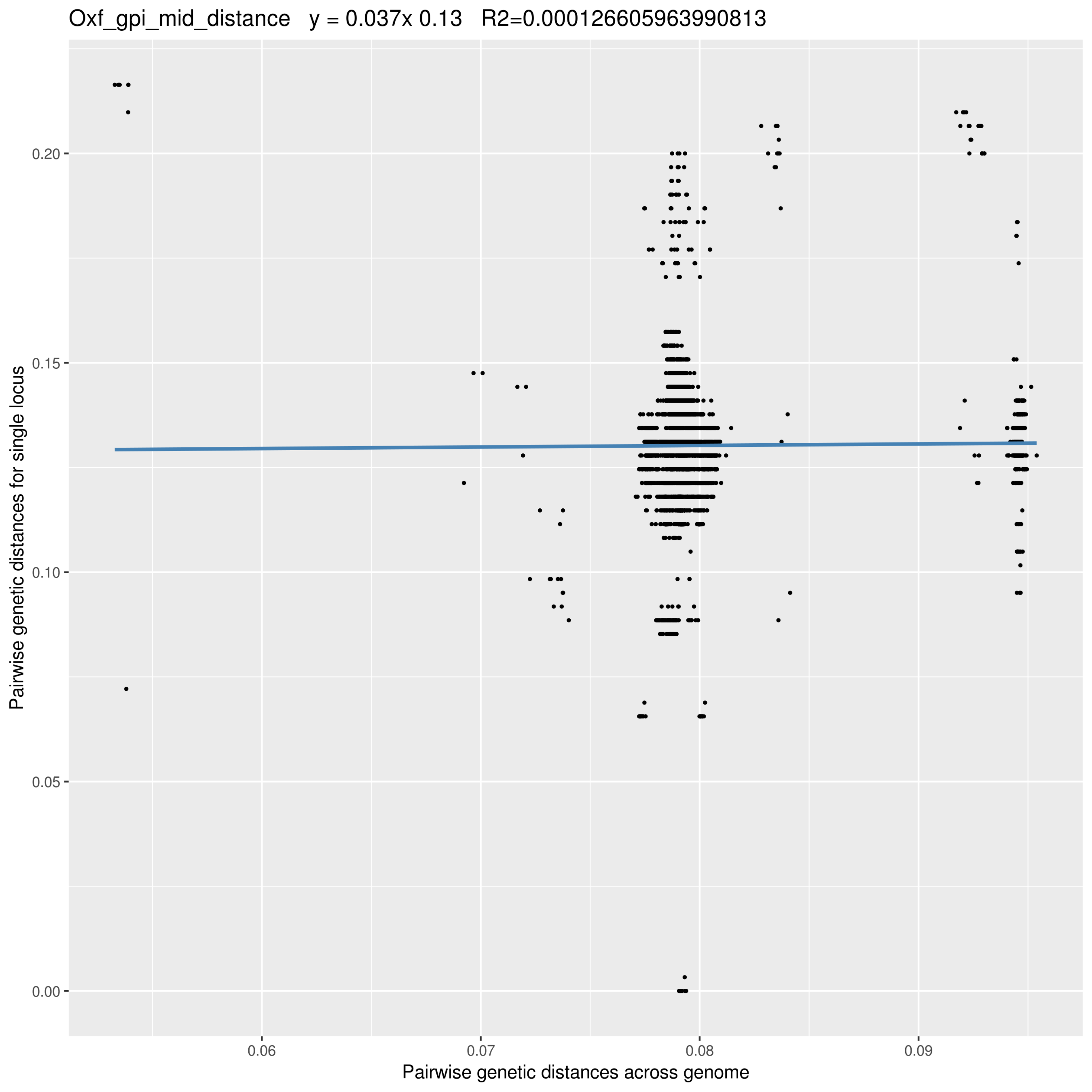
Oxf_gpi_mid_distance y = 0.037x 0.13 R2=0.000126605963990813
Pairwise genetic distances for single locus
0.20
0.15
0.10
0.05
0.00
0.06
0.07
0.08
0.09
Pairwise genetic distances across genome

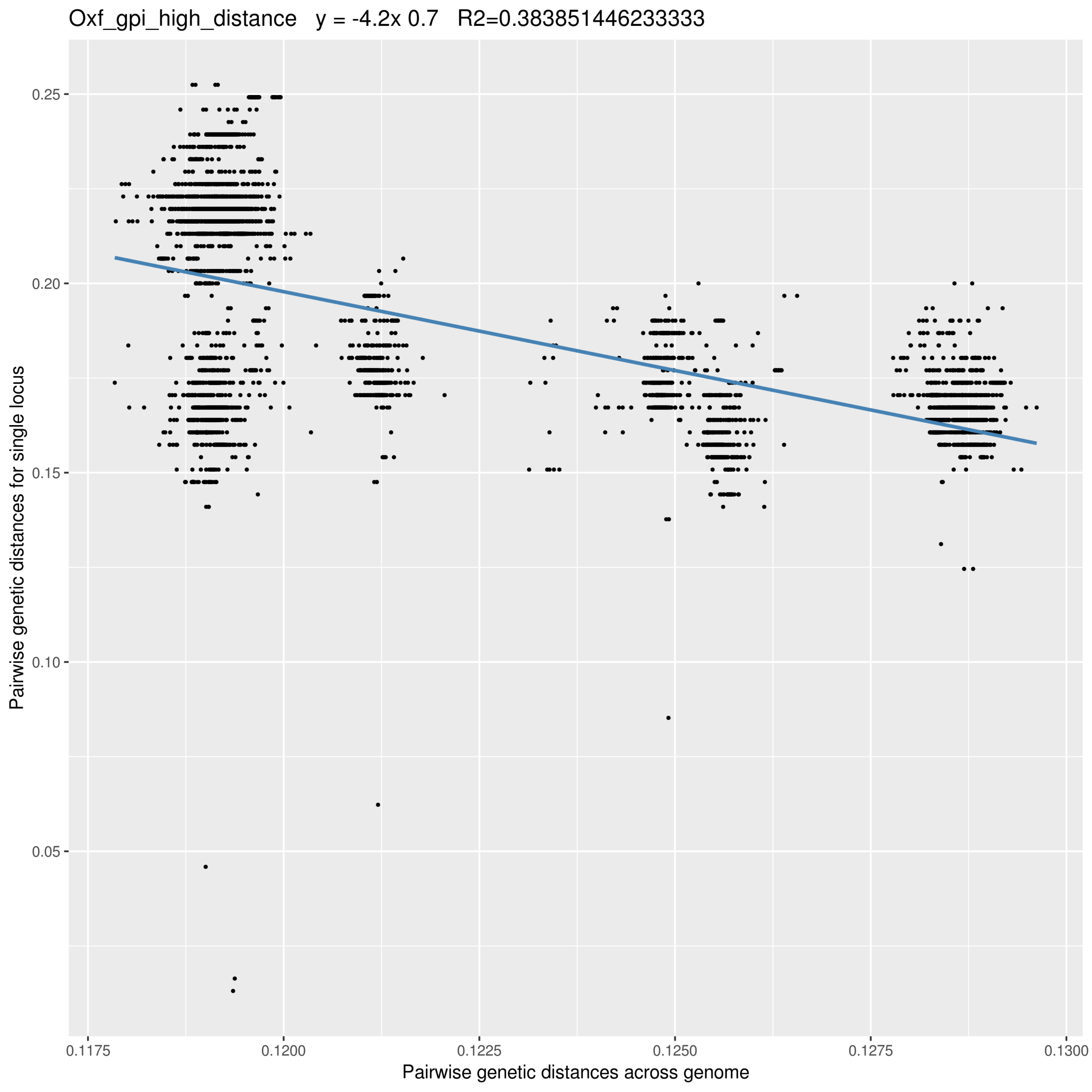

Oxf_gpi_high_distance   y = -4.2x 0.7   R2=0.383851446233333
Pairwise genetic distances for single locus
0.25
0.20
0.15
0.10
0.05
0.1175
0.1200
0.1225
0.1250
0.1275
0.1300
Pairwise genetic distances across genome

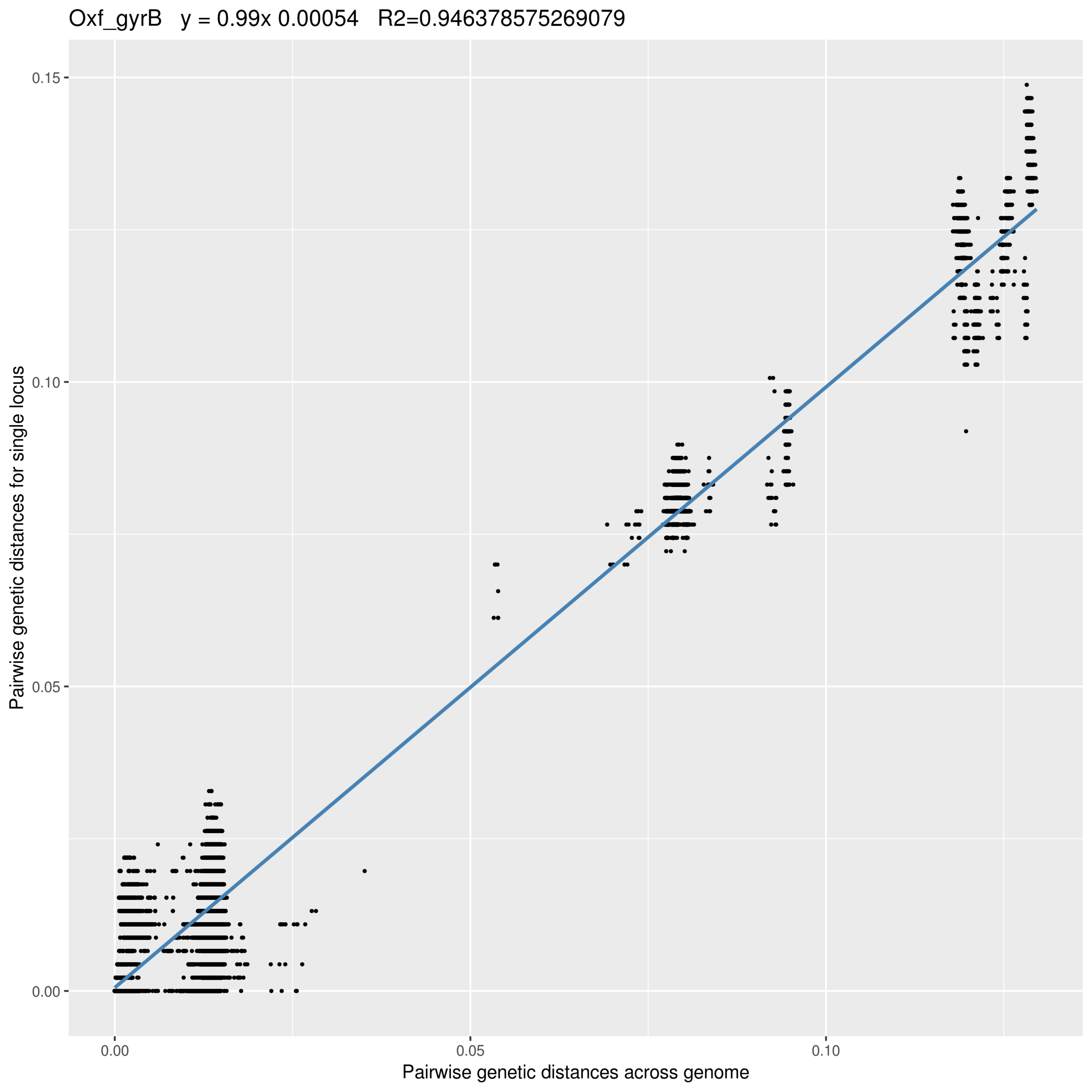
Oxf_gyrB y = 0.99x 0.00054 R2=0.946378575269079
Pairwise genetic distances for single locus
0.15
0.10
0.05
0.00
0.00
0.05
0.10
Pairwise genetic distances across genome

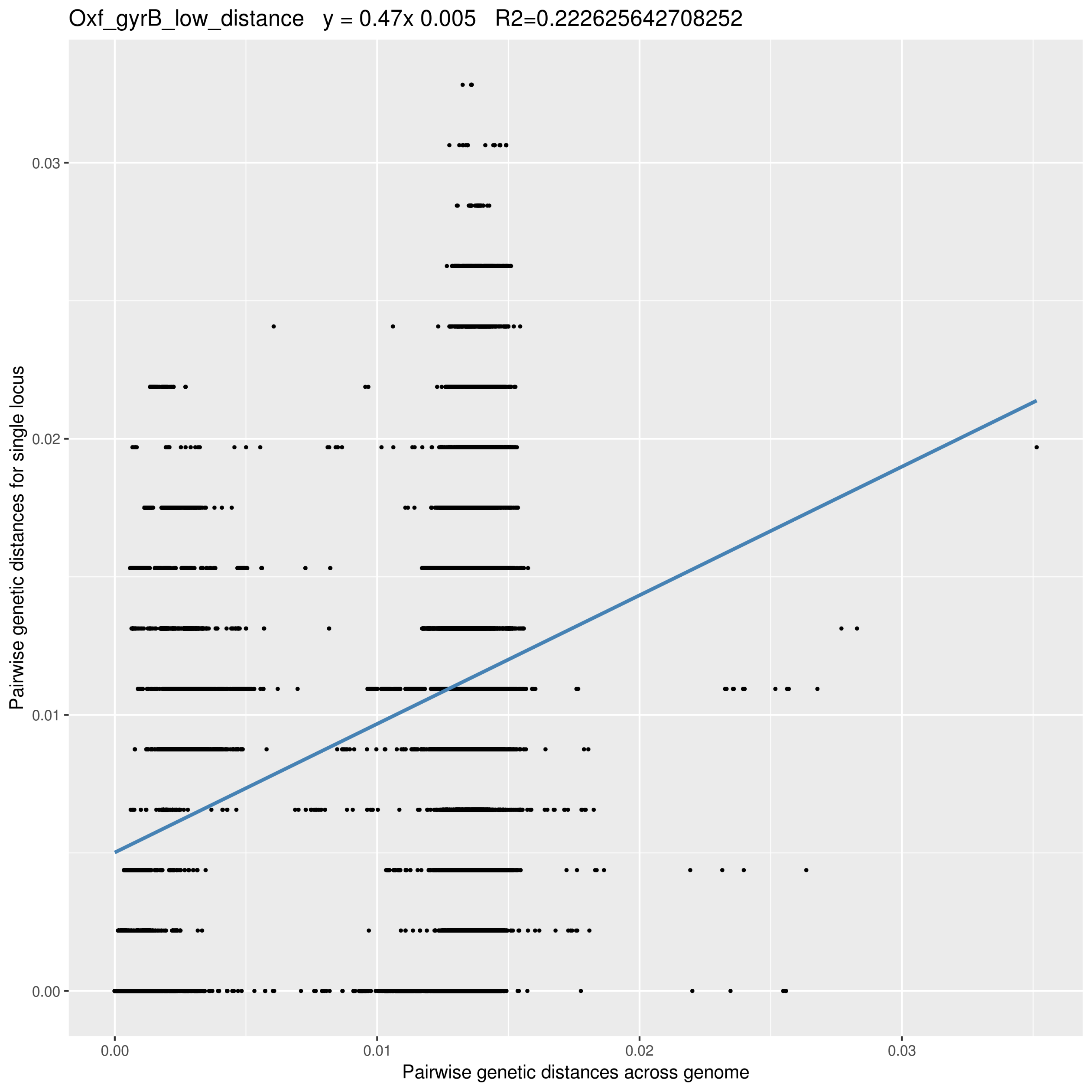

Oxf_gyrB_low_distance   y = 0.47x 0.005   R2=0.222625642708252
Pairwise genetic distances for single locus
0.03
0.02
0.01
0.00
0.00
0.01
0.02
0.03
Pairwise genetic distances across genome

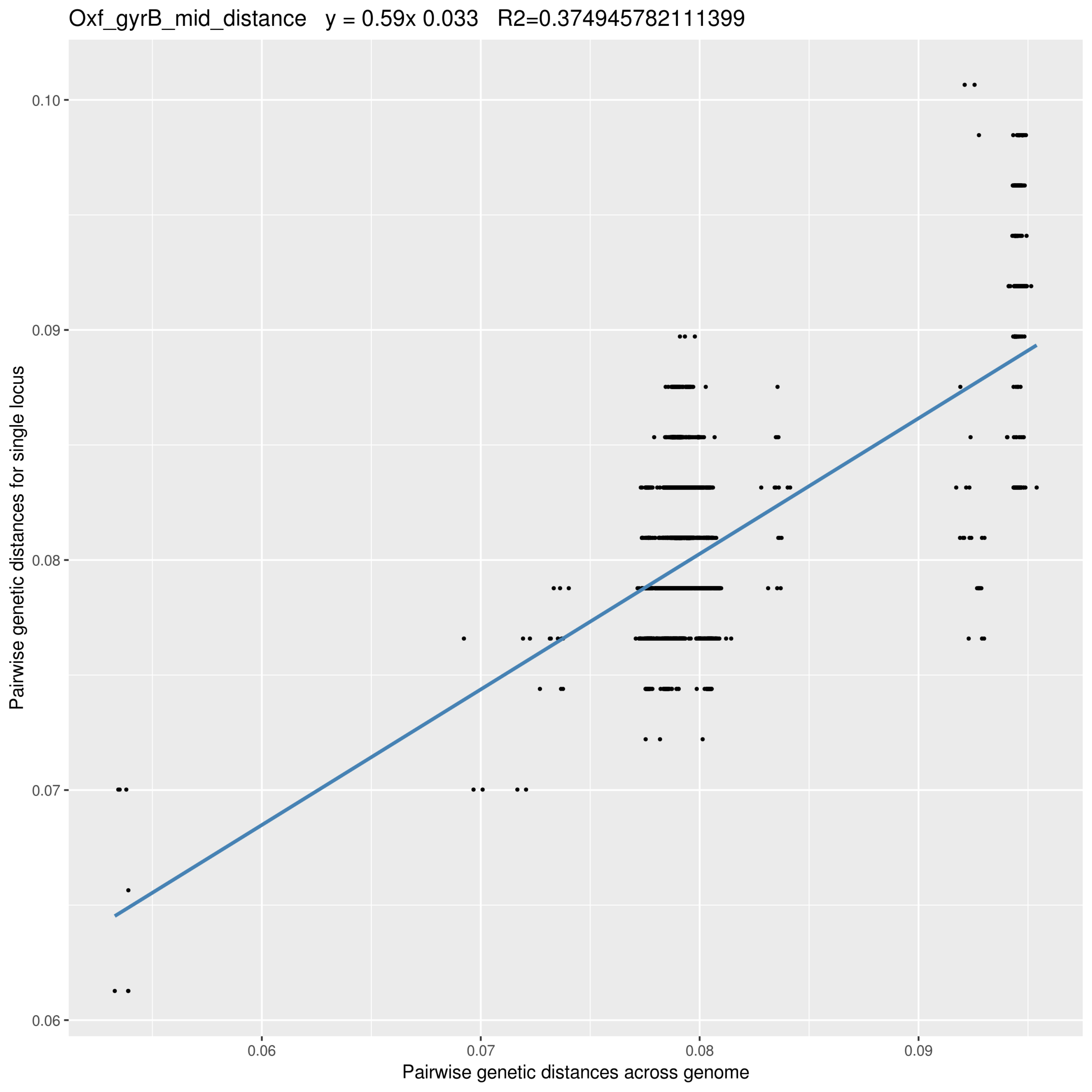

Oxf_gyrB_mid_distance y = 0.59x 0.033 R2=0.374945782111399
Pairwise genetic distances for single locus
0.10
0.09
0.08
0.07
0.06
0.06
0.07
0.08
0.09
Pairwise genetic distances across genome

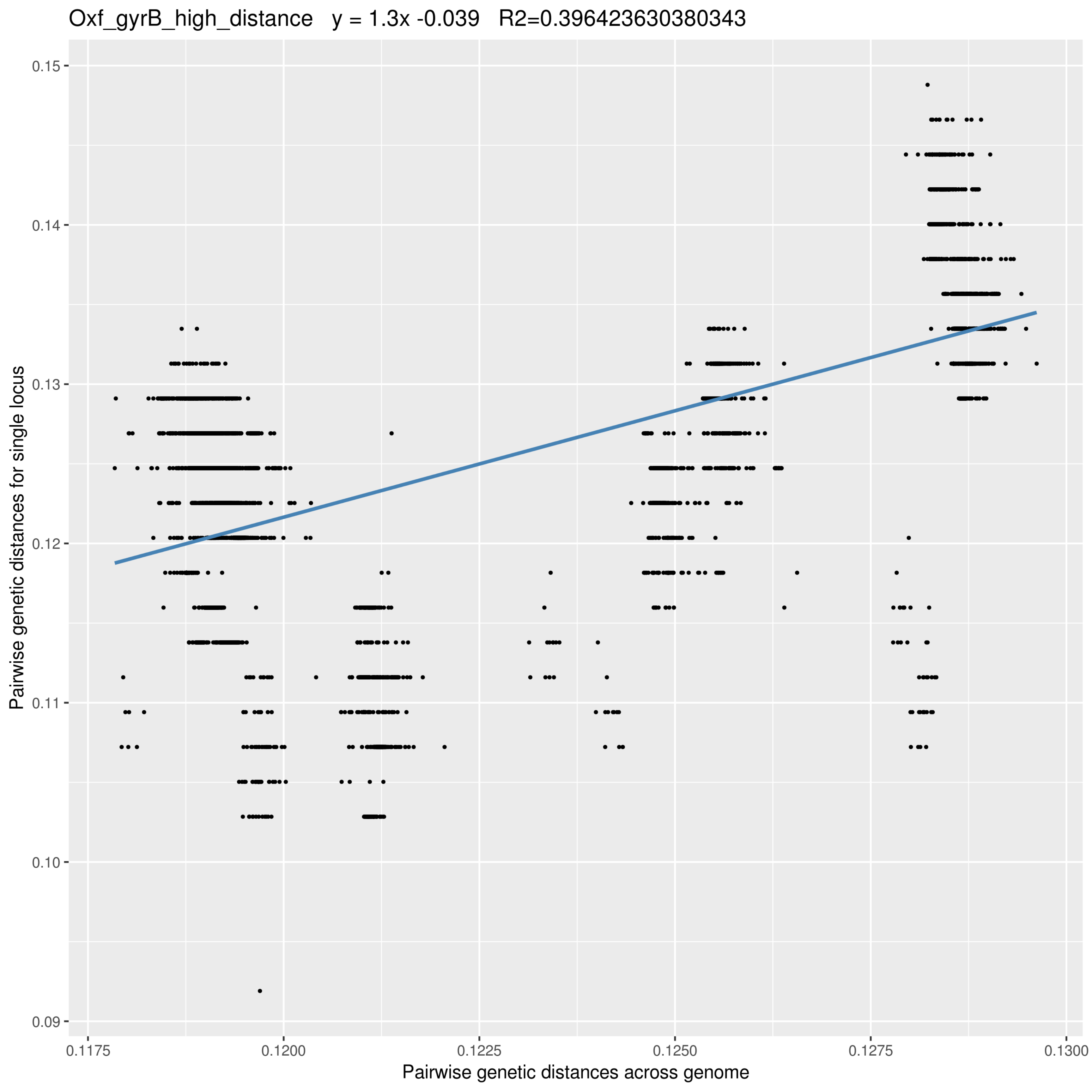

Oxf_gyrB_high_distance   y = 1.3x -0.039   R2=0.396423630380343
Pairwise genetic distances for single locus
0.15
0.14
0.13
0.12
0.11
0.10
0.09
0.1175
0.1200
0.1225
0.1250
0.1275
0.1300
Pairwise genetic distances across genome

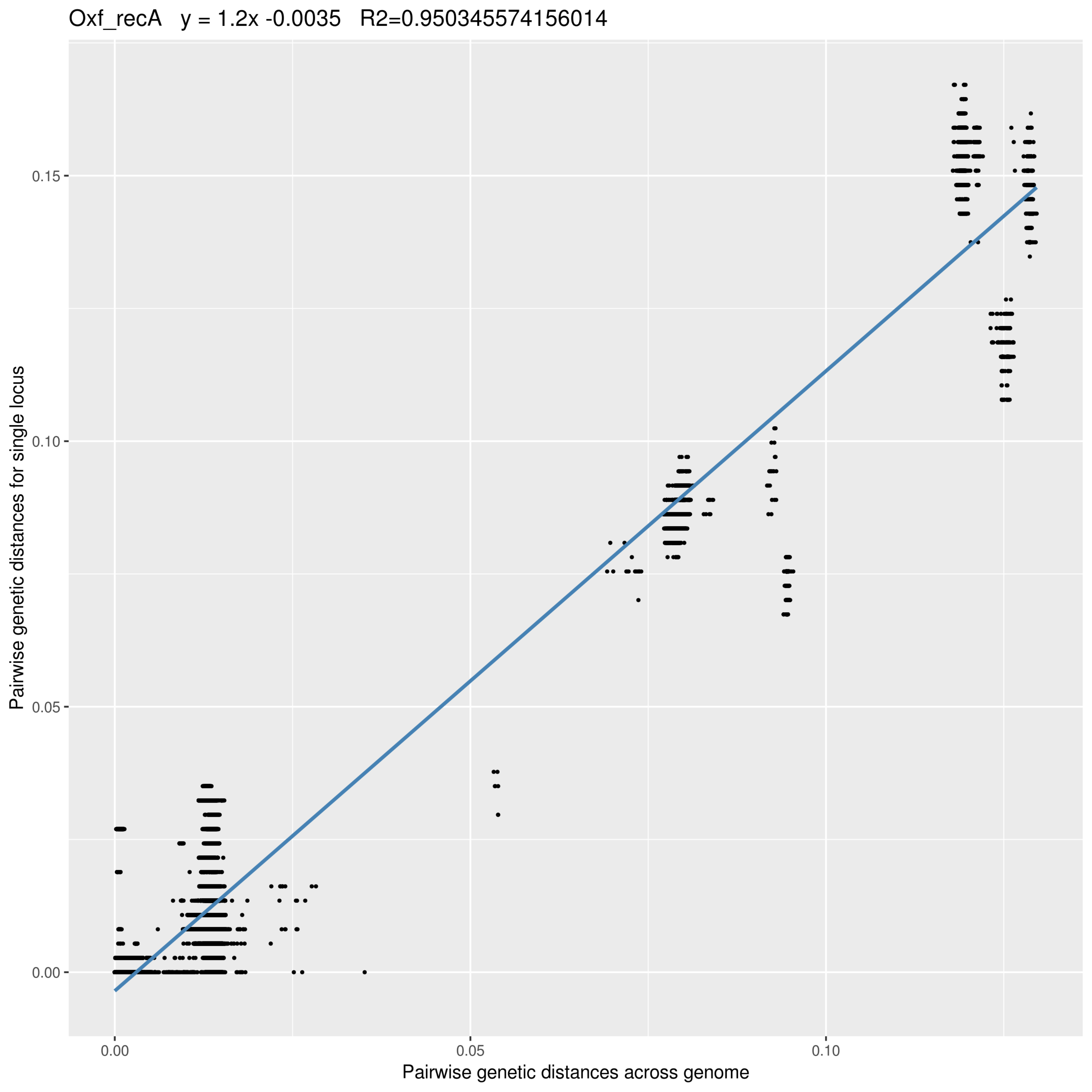

Oxf_recA y = 1.2x -0.0035 R2=0.950345574156014
Pairwise genetic distances for single locus
Pairwise genetic distances across genome
0.00
0.05
0.10
0.15
0.00
0.05
0.10

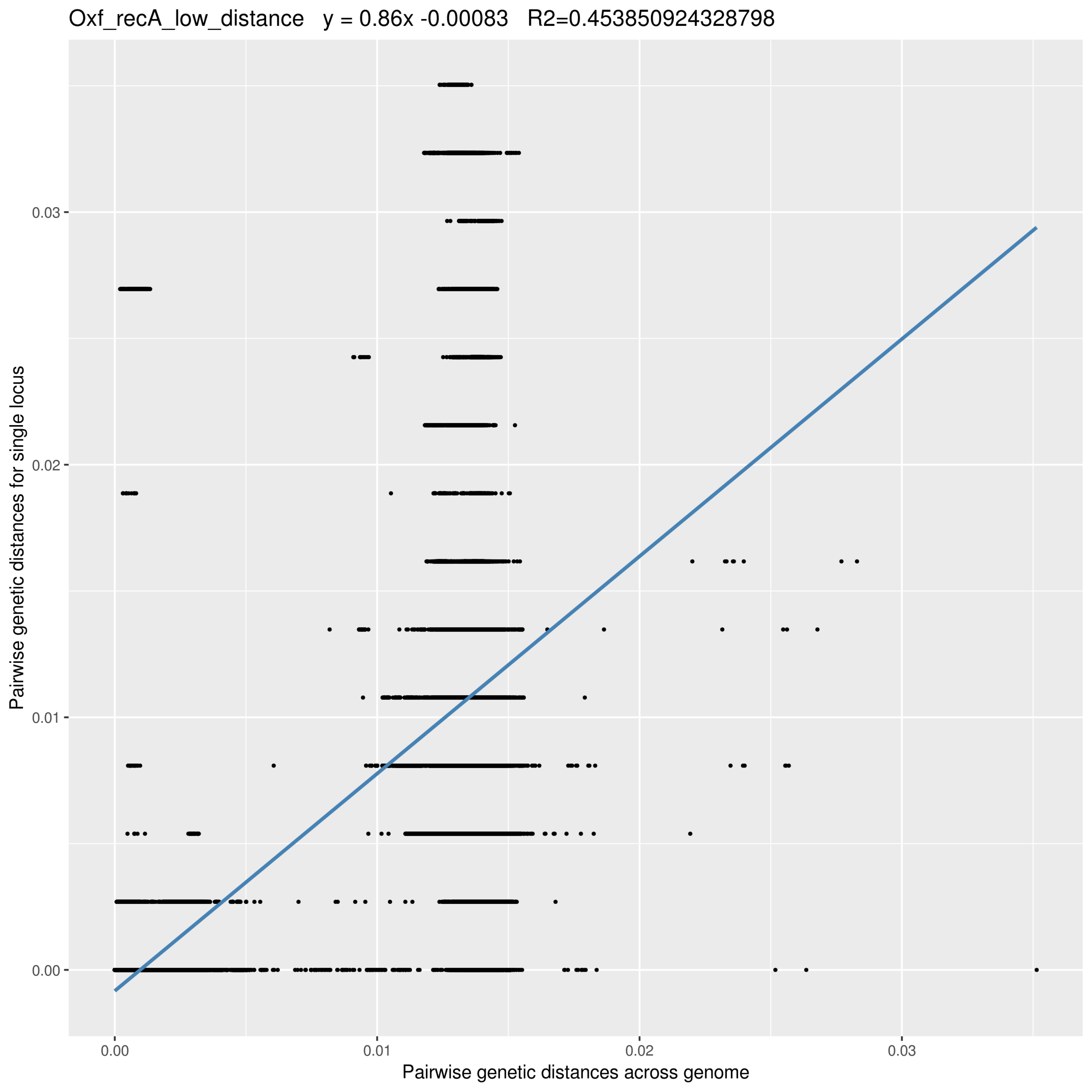
Oxf_recA_low_distance y = 0.86x -0.00083 R2=0.453850924328798
Pairwise genetic distances for single locus
0.03
0.02
0.01
0.00
0.00
0.01
0.02
0.03
Pairwise genetic distances across genome

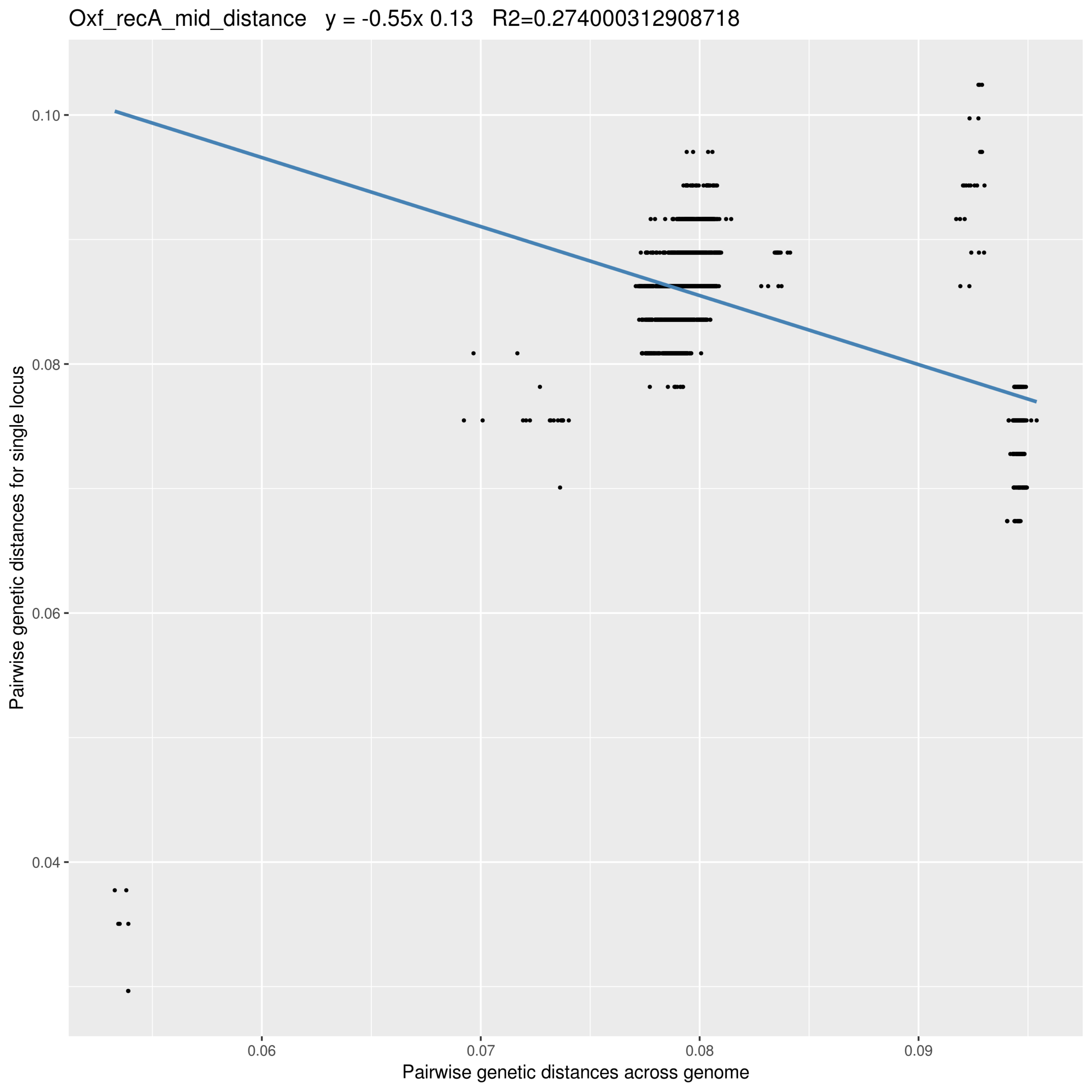
Oxf_recA_mid_distance   y = -0.55x 0.13   R2=0.274000312908718
Pairwise genetic distances for single locus
0.10
0.08
0.06
0.04
0.06
0.07
0.08
0.09
Pairwise genetic distances across genome

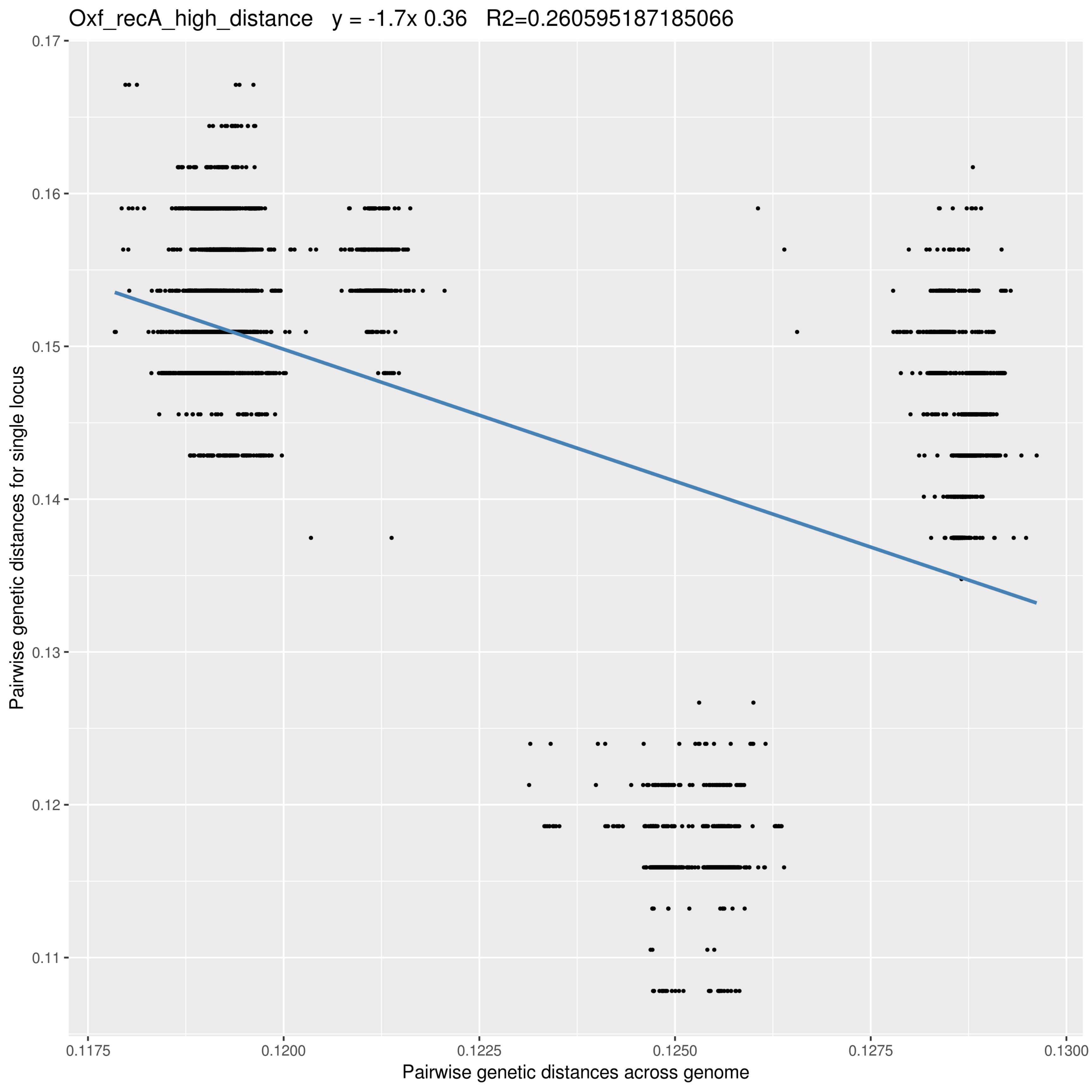

Oxf_recA_high_distance y = -1.7x 0.36 R2=0.260595187185066
0.17
0.16
0.15
0.14
0.13
0.12
0.11
Pairwise genetic distances for single locus
0.1175
0.1200
0.1225
0.1250
0.1275
0.1300
Pairwise genetic distances across genome

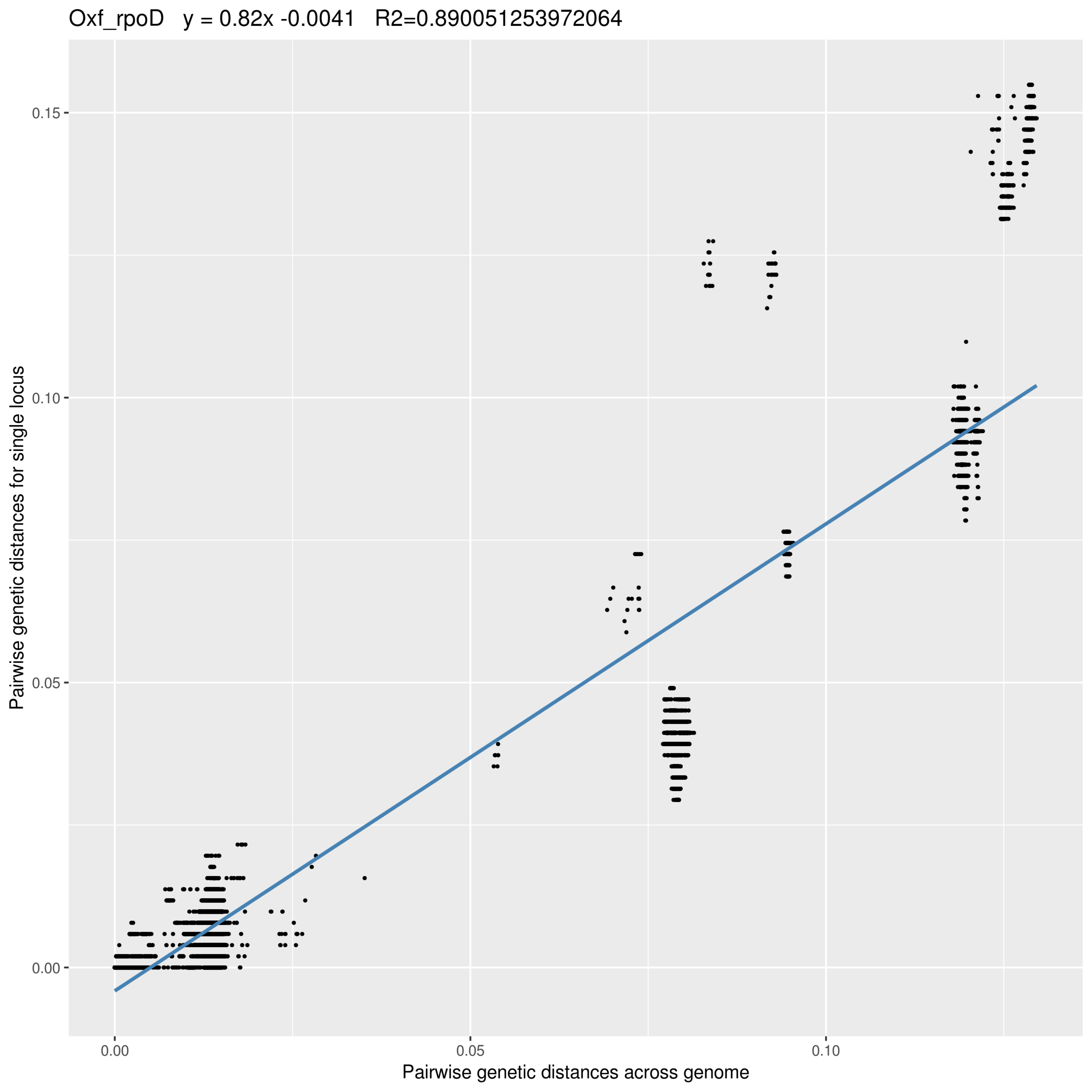

Oxf_rpoD y = 0.82x -0.0041 R2=0.890051253972064
Pairwise genetic distances for single locus
0.15
0.10
0.05
0.00
0.00
0.05
0.10
Pairwise genetic distances across genome

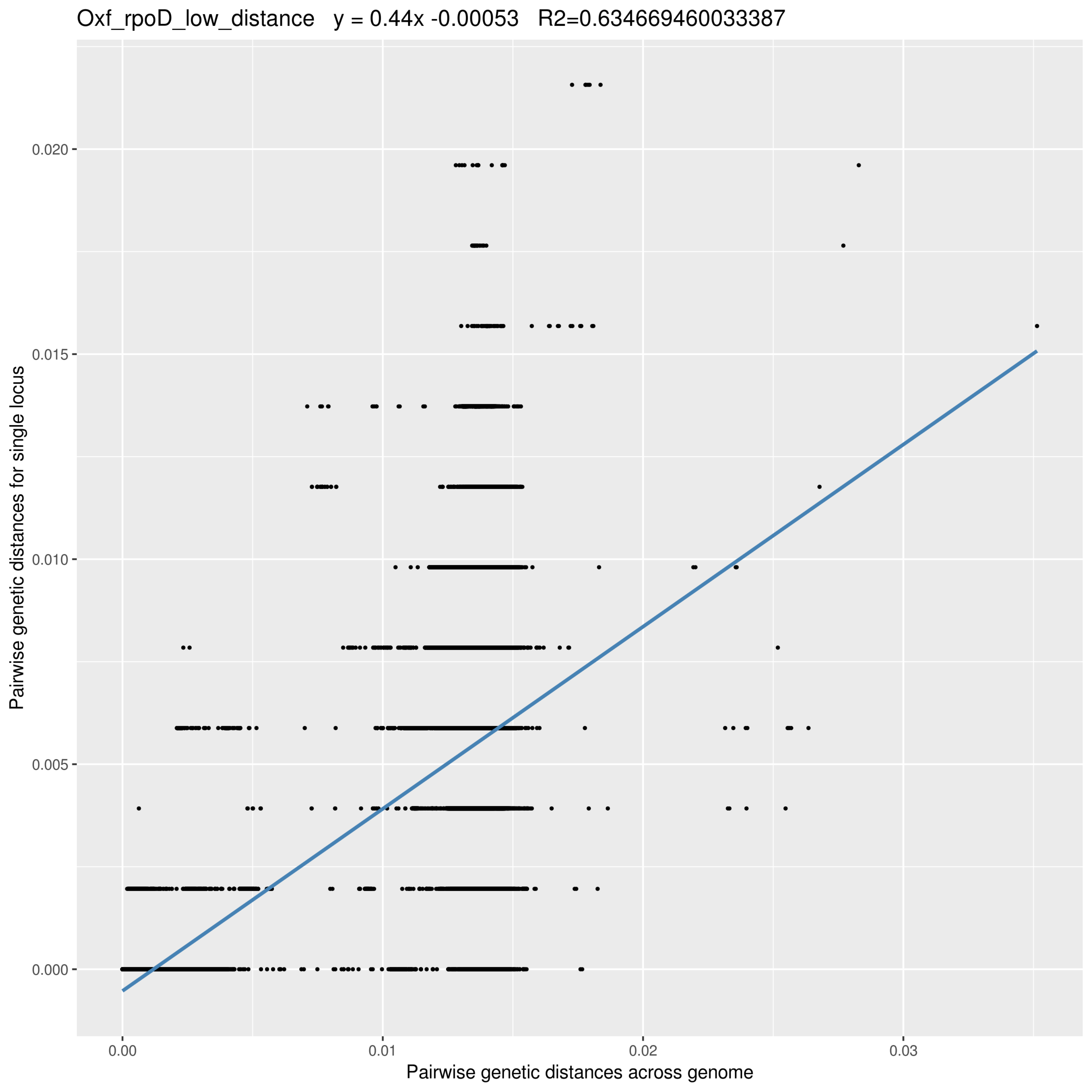

Oxf_rpoD_low_distance   y = 0.44x -0.00053   R2=0.634669460033387
Pairwise genetic distances for single locus
0.000
0.005
0.010
0.015
0.020
0.00
0.01
0.02
0.03
Pairwise genetic distances across genome

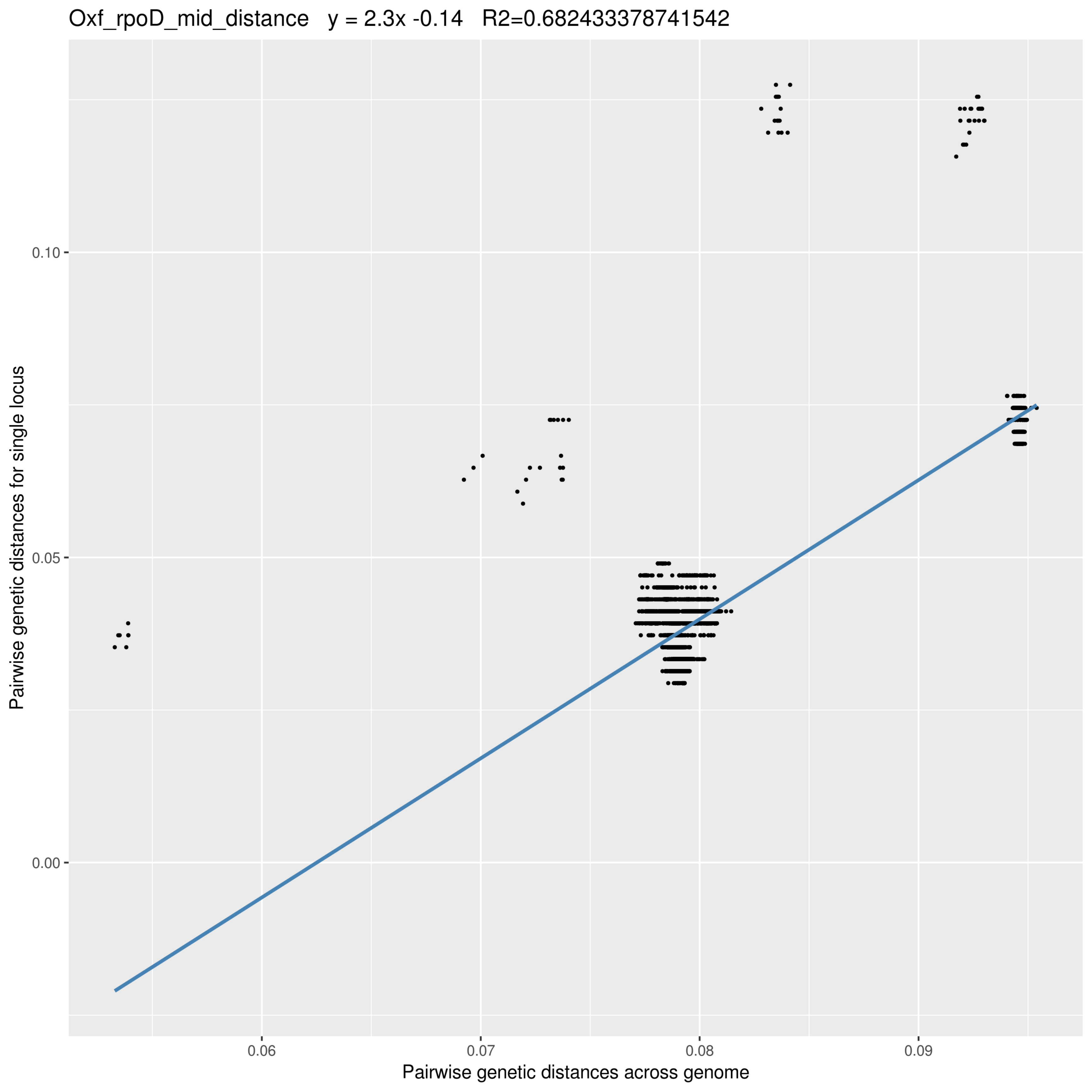

Oxf_rpoD_mid_distance y = 2.3x -0.14 R2=0.682433378741542
0.10
0.05
0.00
0.06
0.07
0.08
0.09
Pairwise genetic distances for single locus
Pairwise genetic distances across genome

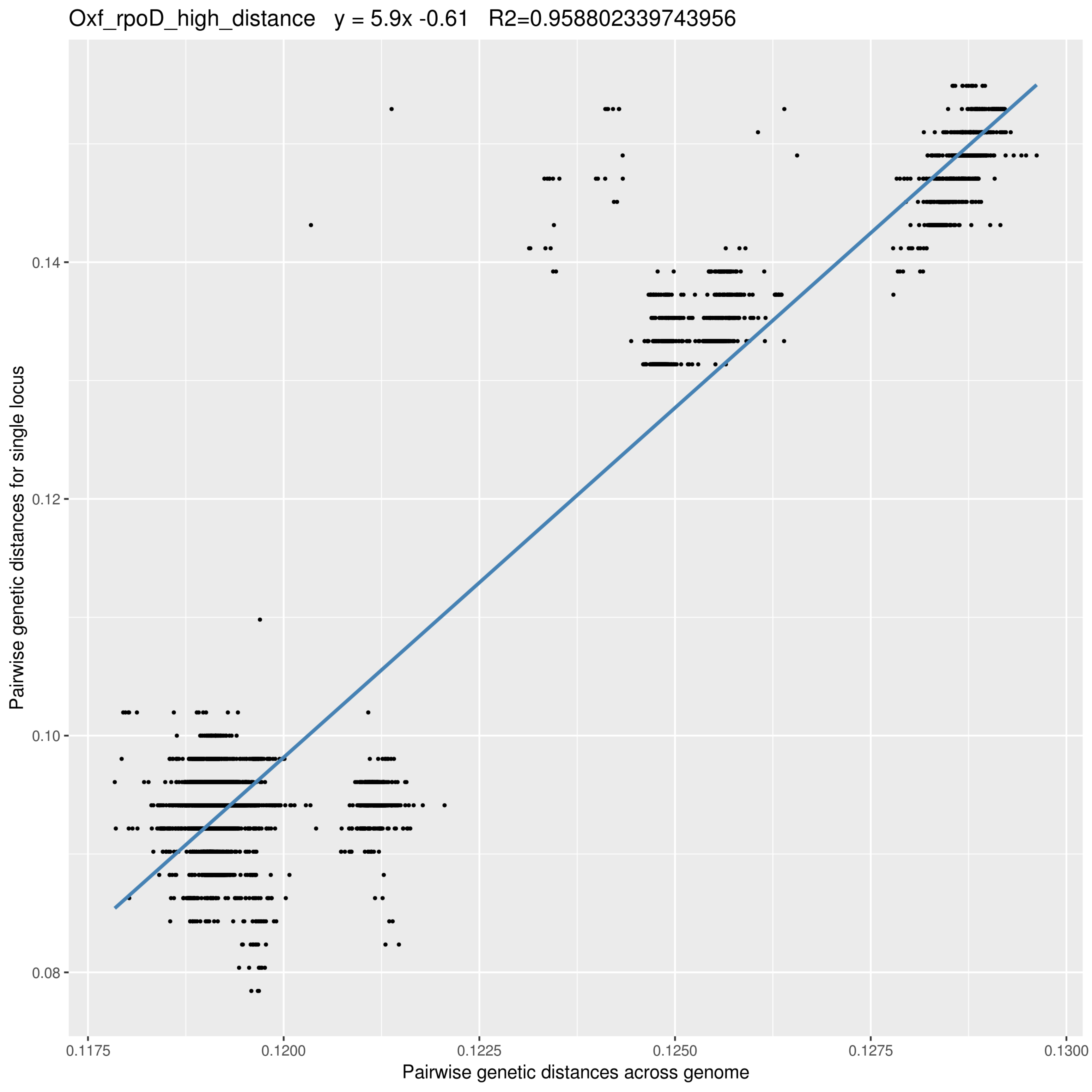

Oxf_rpoD_high_distance y = 5.9x -0.61 R2=0.958802339743956
Pairwise genetic distances for single locus
0.14
0.12
0.10
0.08
0.1175
0.1200
0.1225
0.1250
0.1275
0.1300
Pairwise genetic distances across genome

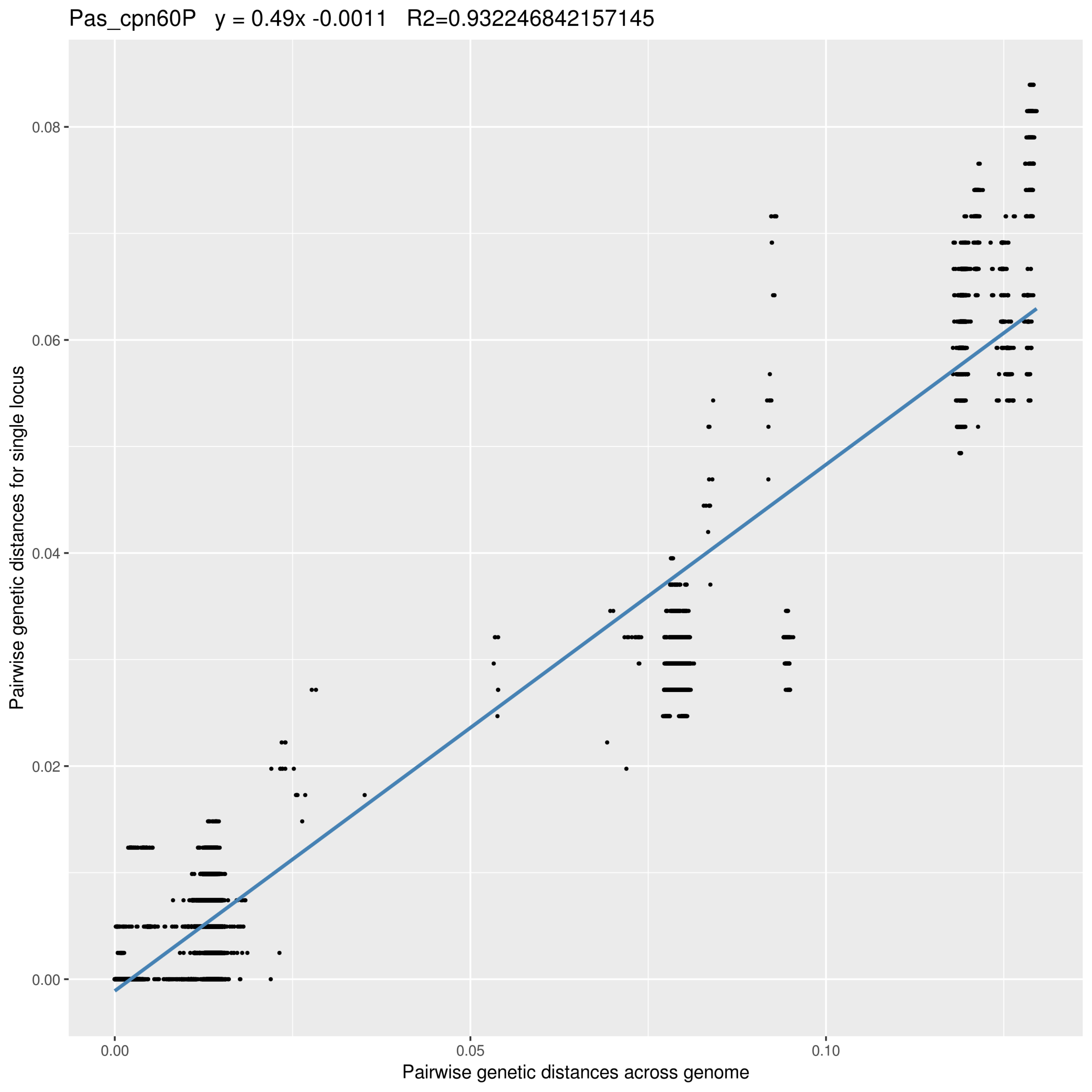

Pas_cpn60P   y = 0.49x -0.0011   R2=0.932246842157145
Pairwise genetic distances for single locus
0.00
0.02
0.04
0.06
0.08
0.00
0.05
0.10
Pairwise genetic distances across genome

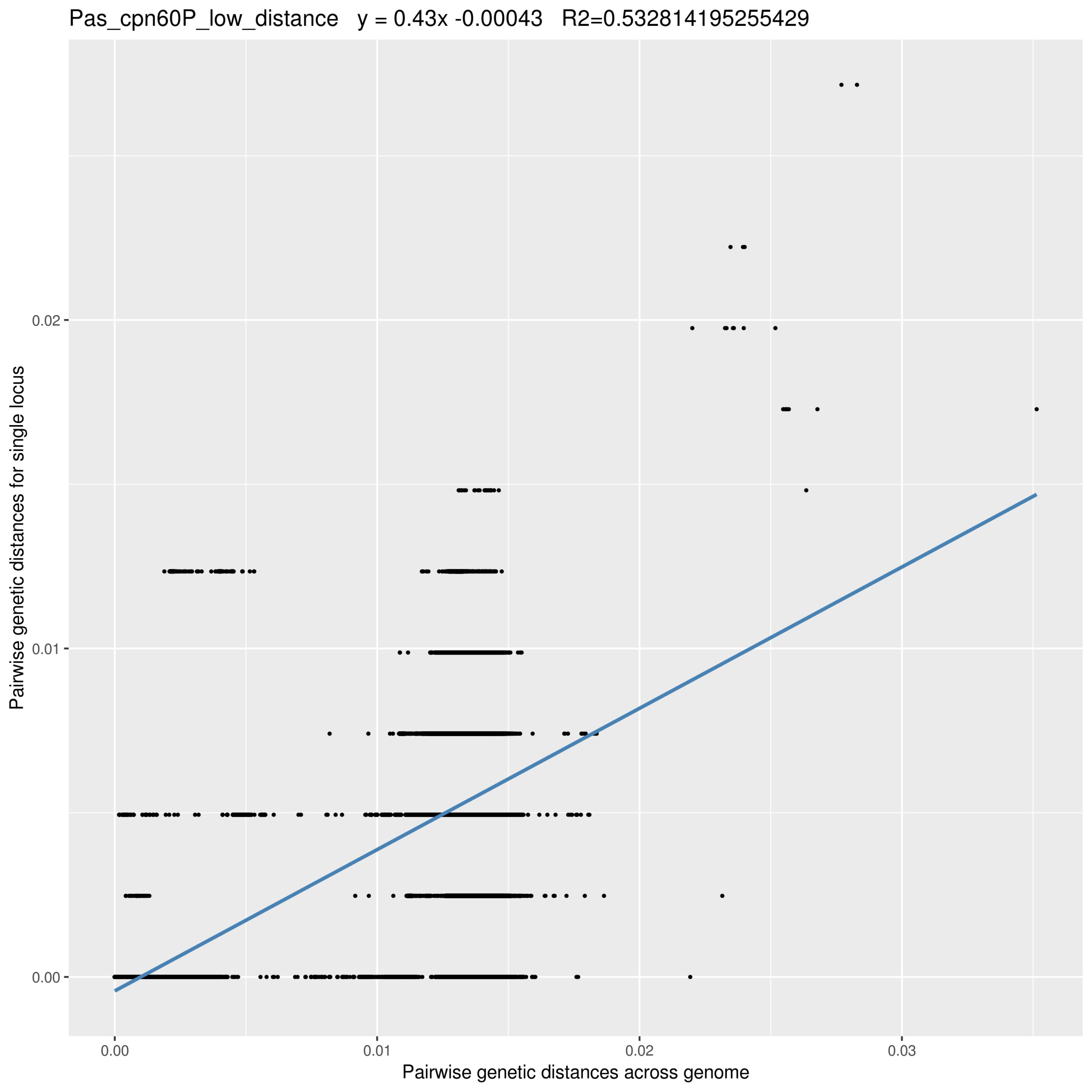
Pas_cpn60P_low_distance y = 0.43x -0.00043 R2=0.532814195255429
Pairwise genetic distances for single locus
0.02
0.01
0.00
0.00
0.01
0.02
0.03
Pairwise genetic distances across genome

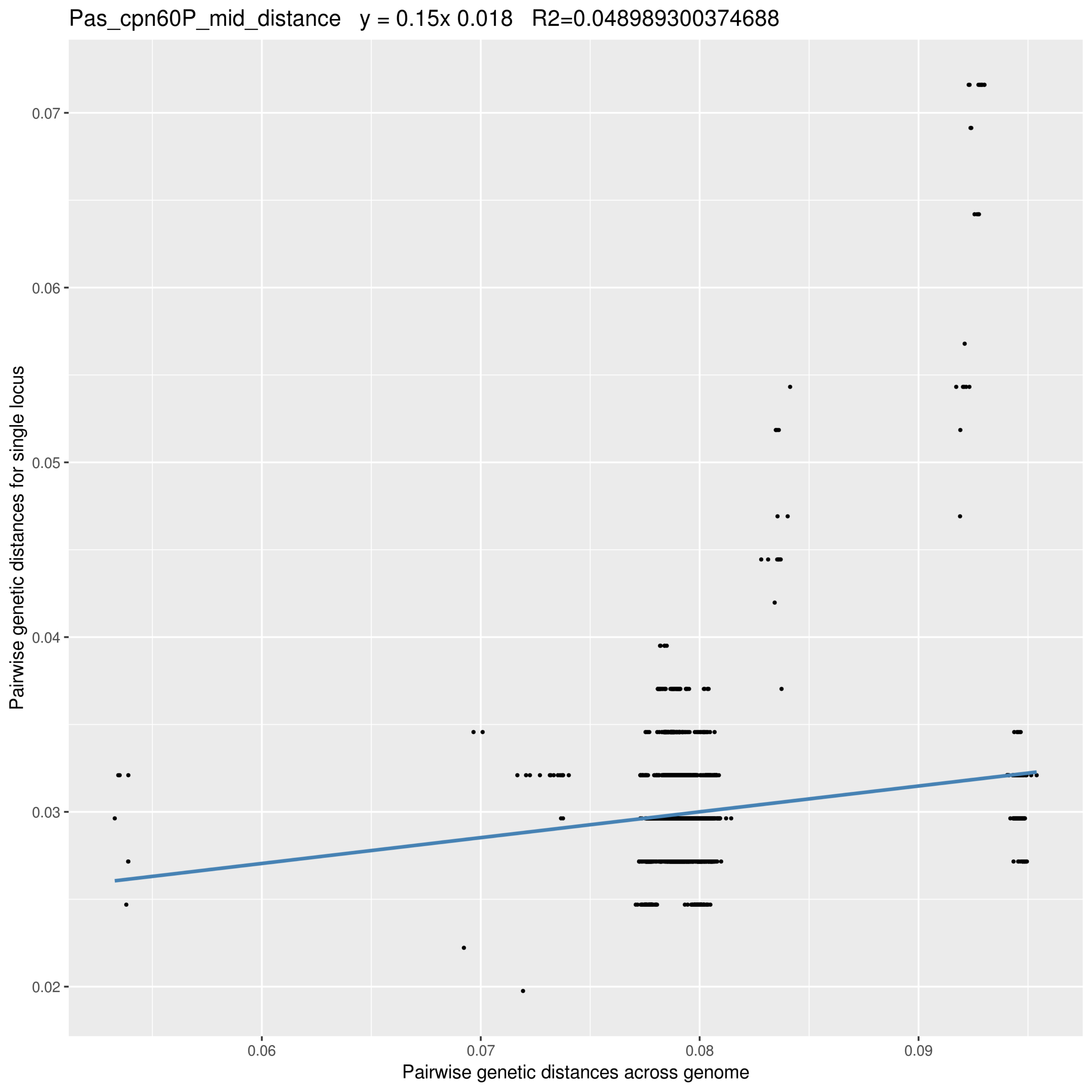

Pas_cpn60P_mid_distance y = 0.15x 0.018 R2=0.048989300374688
Pairwise genetic distances for single locus
0.07
0.06
0.05
0.04
0.03
0.02
0.06
0.07
0.08
0.09
Pairwise genetic distances across genome

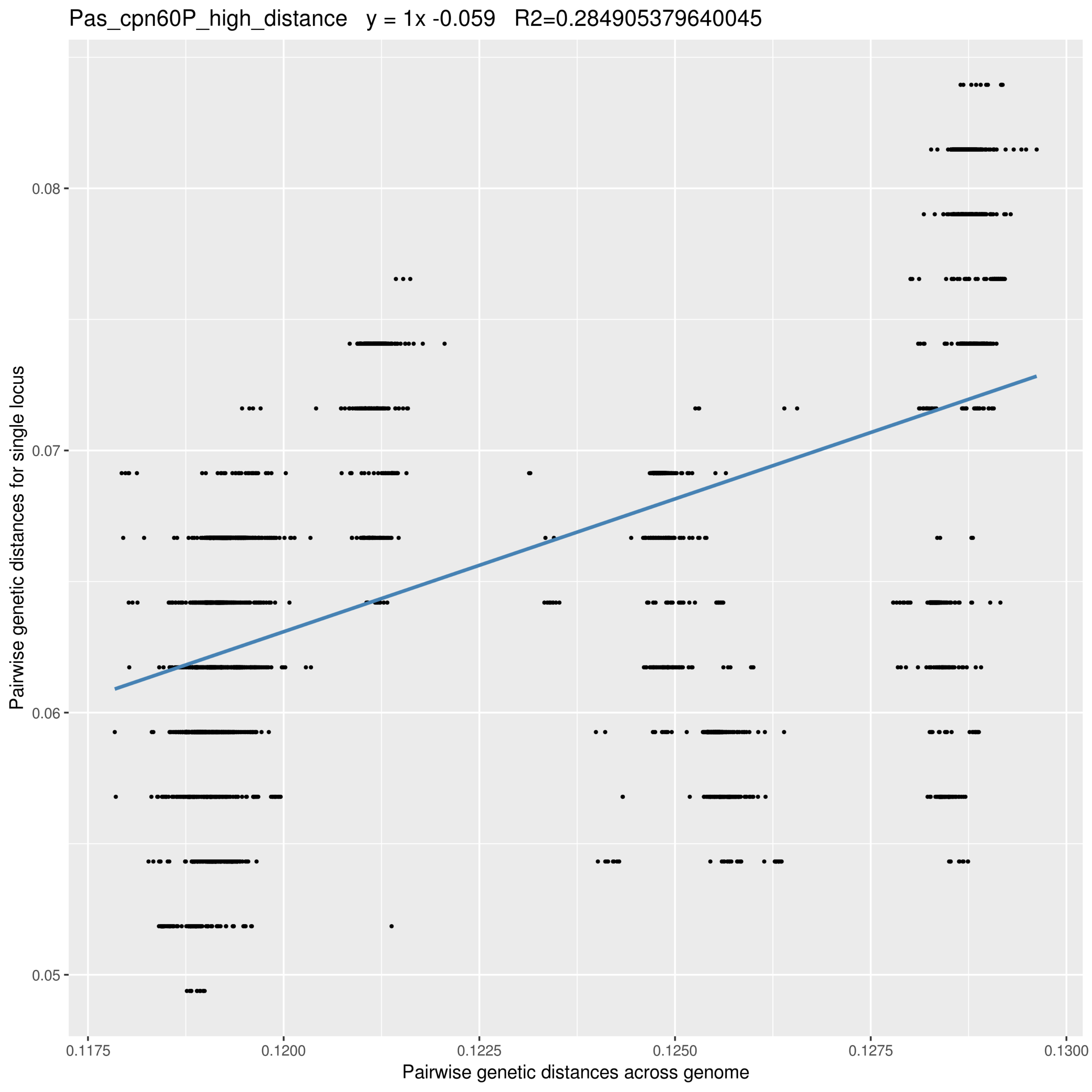
Pas_cpn60P_high_distance y = 1x -0.059 R2=0.284905379640045
Pairwise genetic distances for single locus
0.08
0.07
0.06
0.05
0.1175
0.1200
0.1225
0.1250
0.1275
0.1300
Pairwise genetic distances across genome

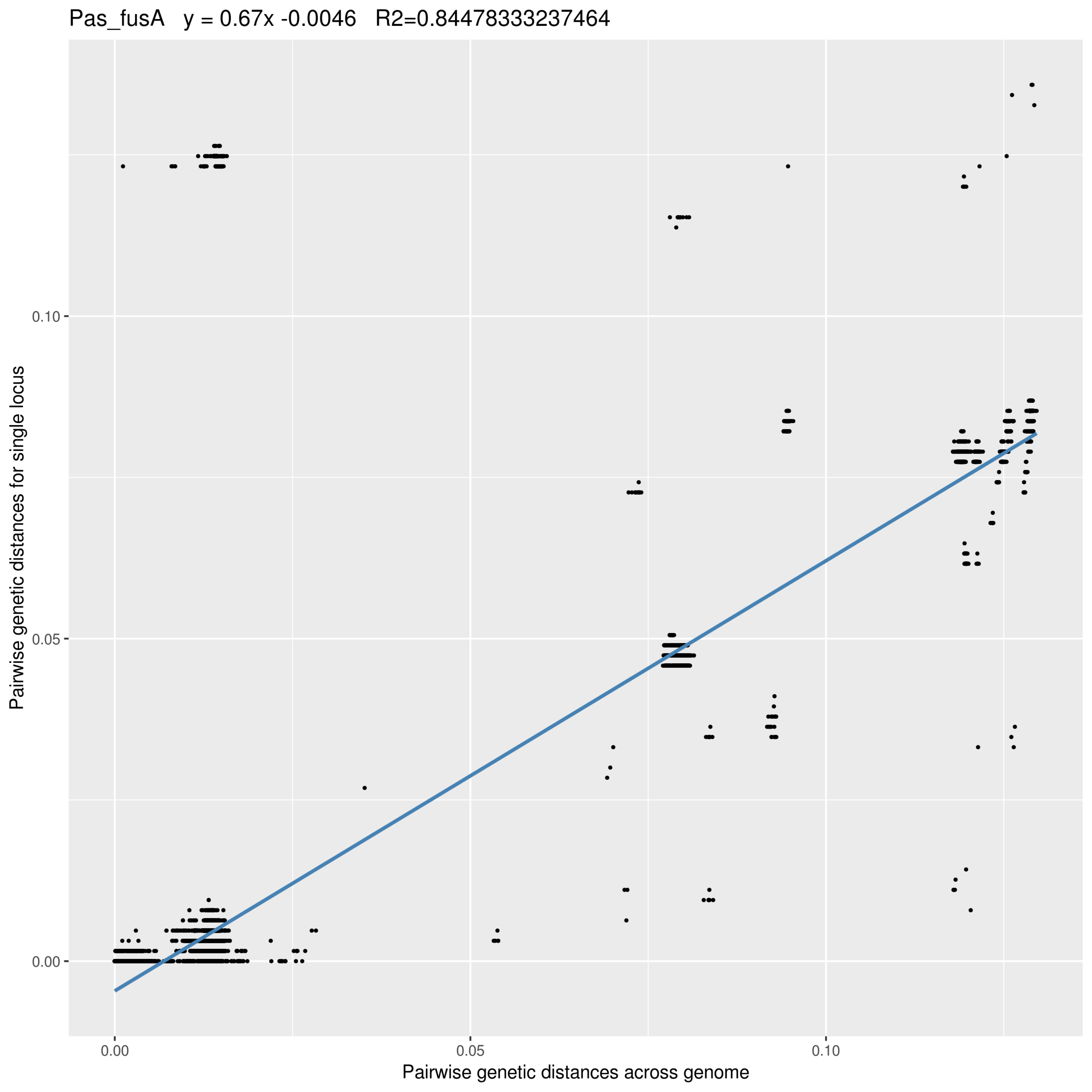
Pas_fusA   y = 0.67x -0.0046   R2=0.84478333237464
Pairwise genetic distances for single locus
0.10
0.05
0.00
0.00
0.05
0.10
Pairwise genetic distances across genome

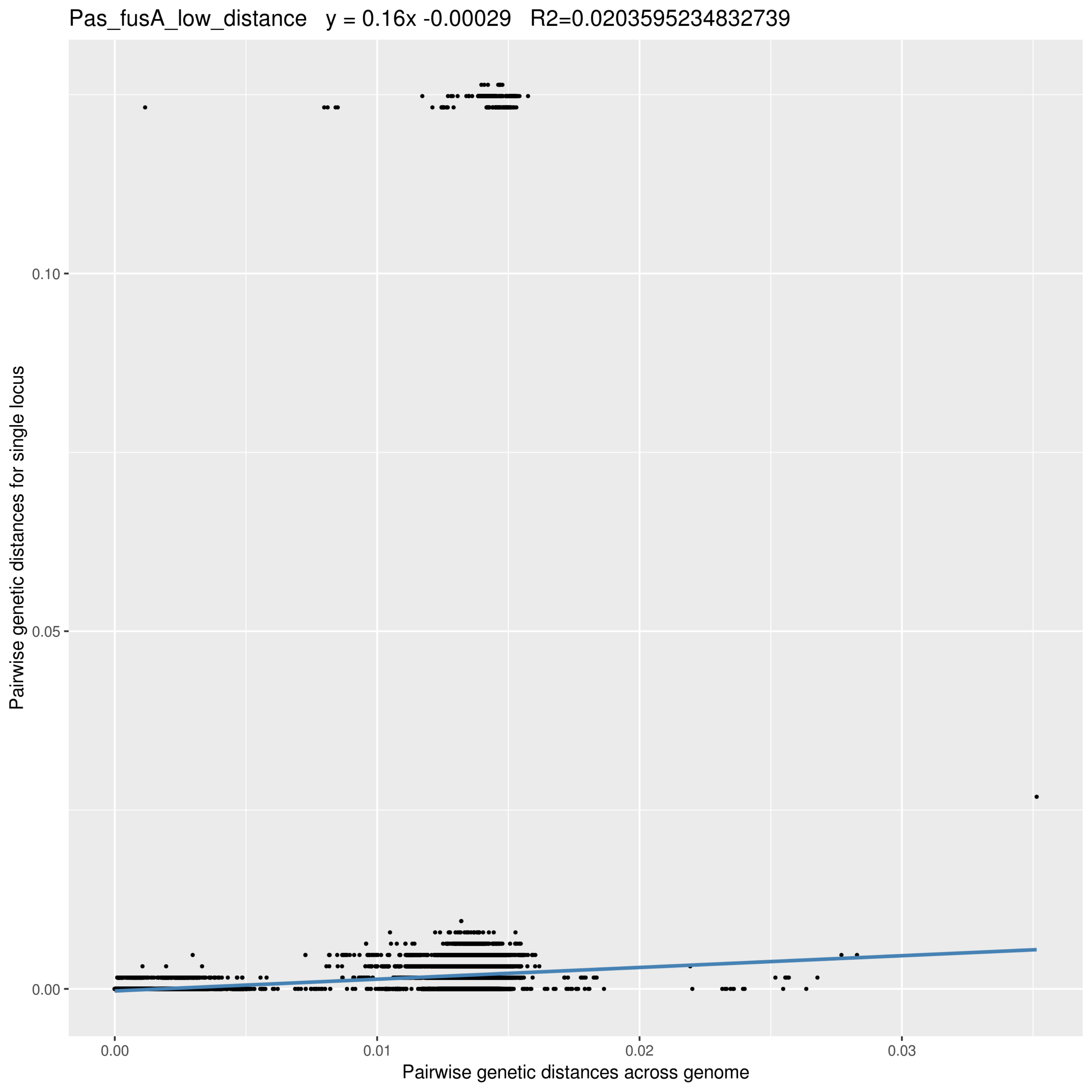
Pas_fusA_low_distance   y = 0.16x -0.00029   R2=0.0203595234832739
Pairwise genetic distances for single locus
0.10
0.05
0.00
0.00
0.01
0.02
0.03
Pairwise genetic distances across genome

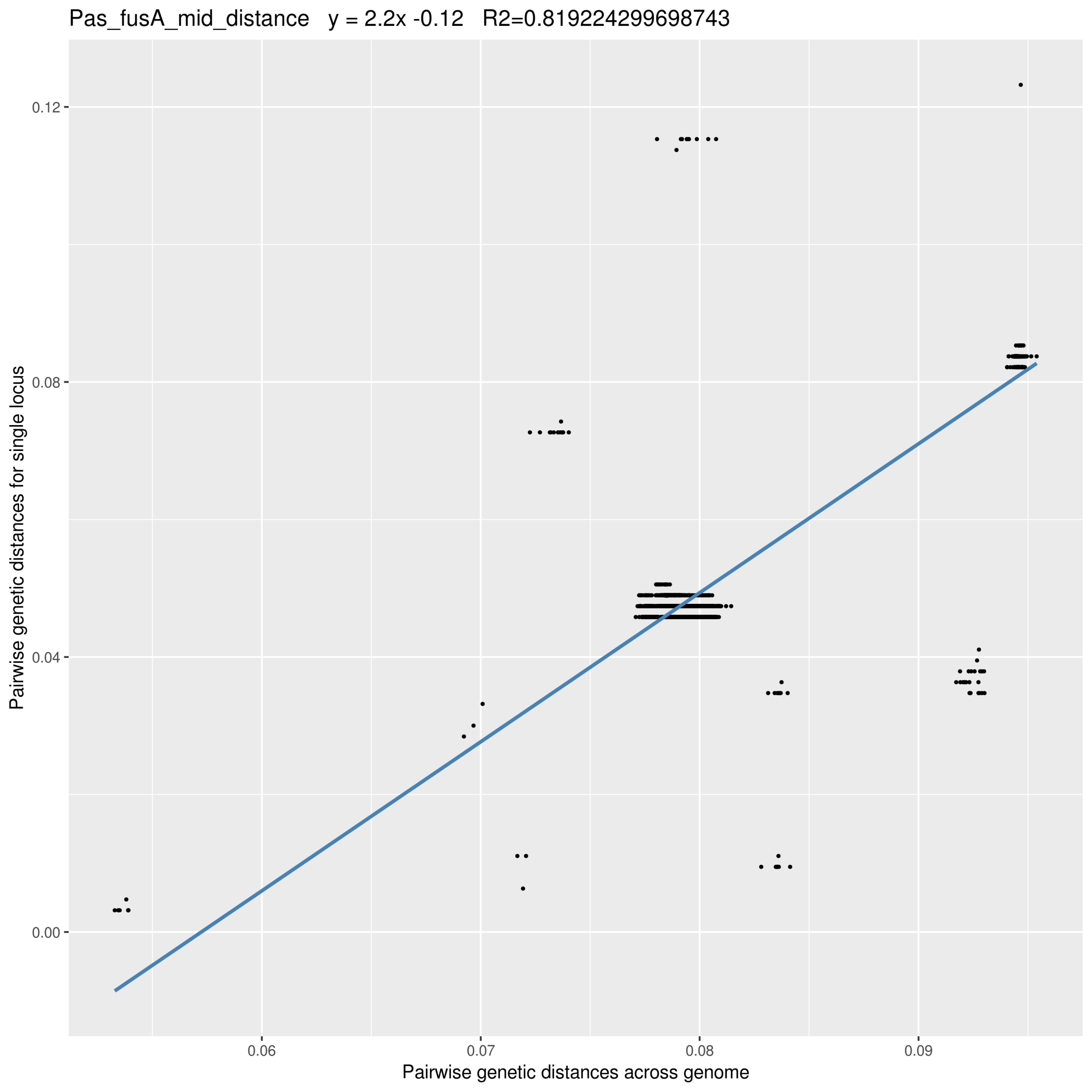

Pas_fusA_mid_distance   y = 2.2x -0.12   R2=0.819224299698743
0.12
0.08
0.04
0.00
0.06
0.07
0.08
0.09
Pairwise genetic distances for single locus
Pairwise genetic distances across genome

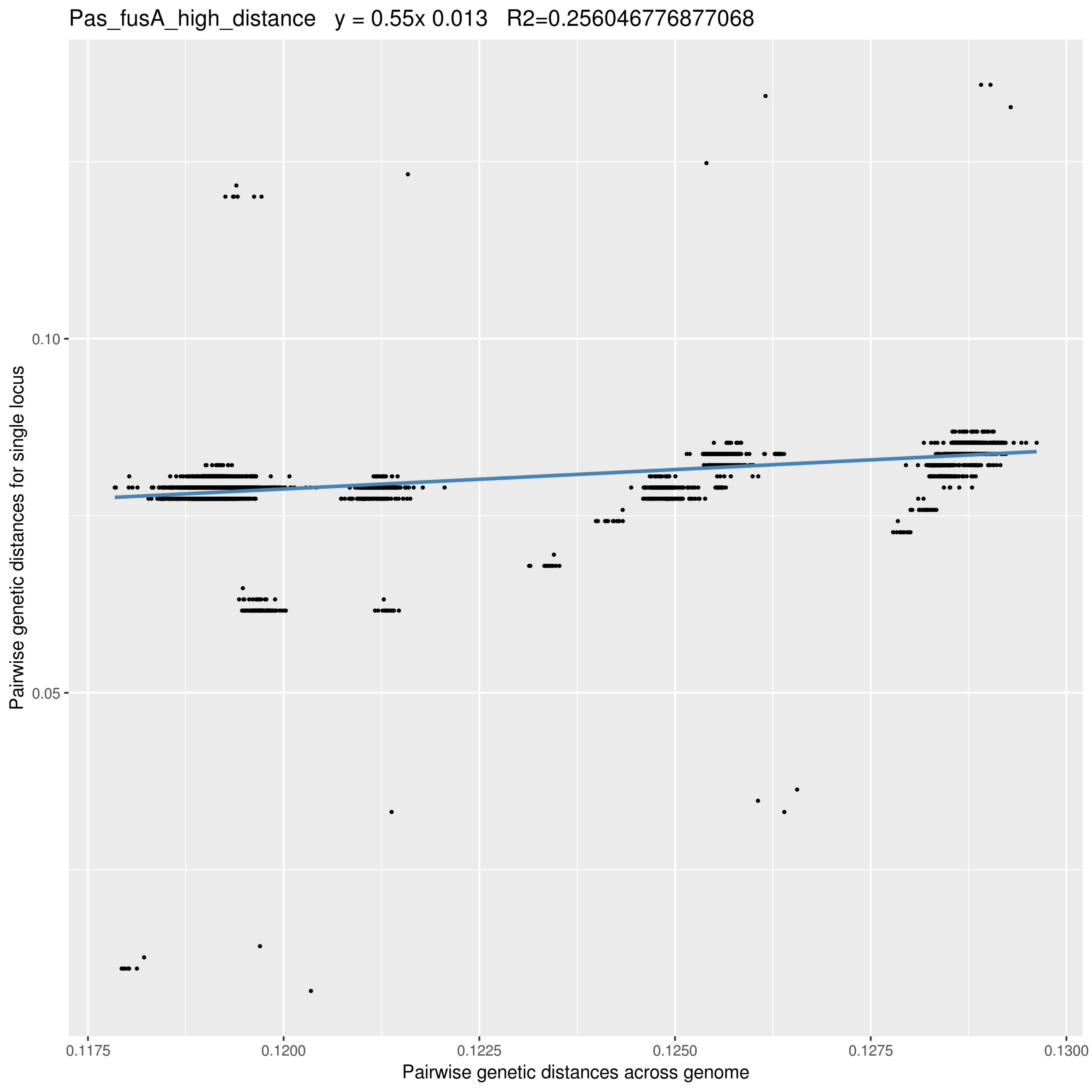

Pas_fusA_high_distance y = 0.55x 0.013 R2=0.256046776877068
Pairwise genetic distances for single locus
0.10
0.05
0.1175
0.1200
0.1225
0.1250
0.1275
0.1300
Pairwise genetic distances across genome

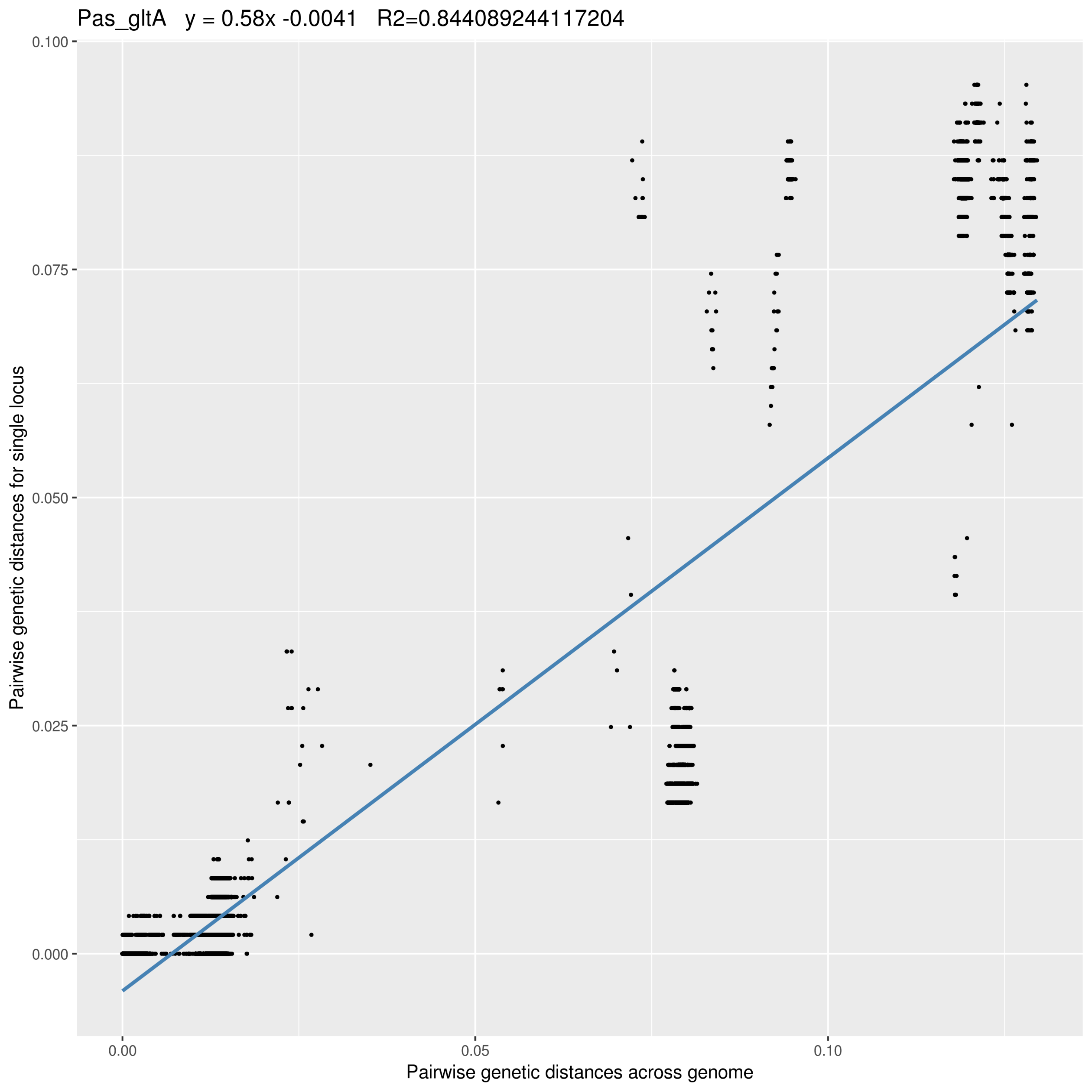

Pas_gltA   y = 0.58x -0.0041   R2=0.844089244117204
0.100
0.075
0.050
0.025
0.000
0.00
0.05
0.10
Pairwise genetic distances for single locus
Pairwise genetic distances across genome

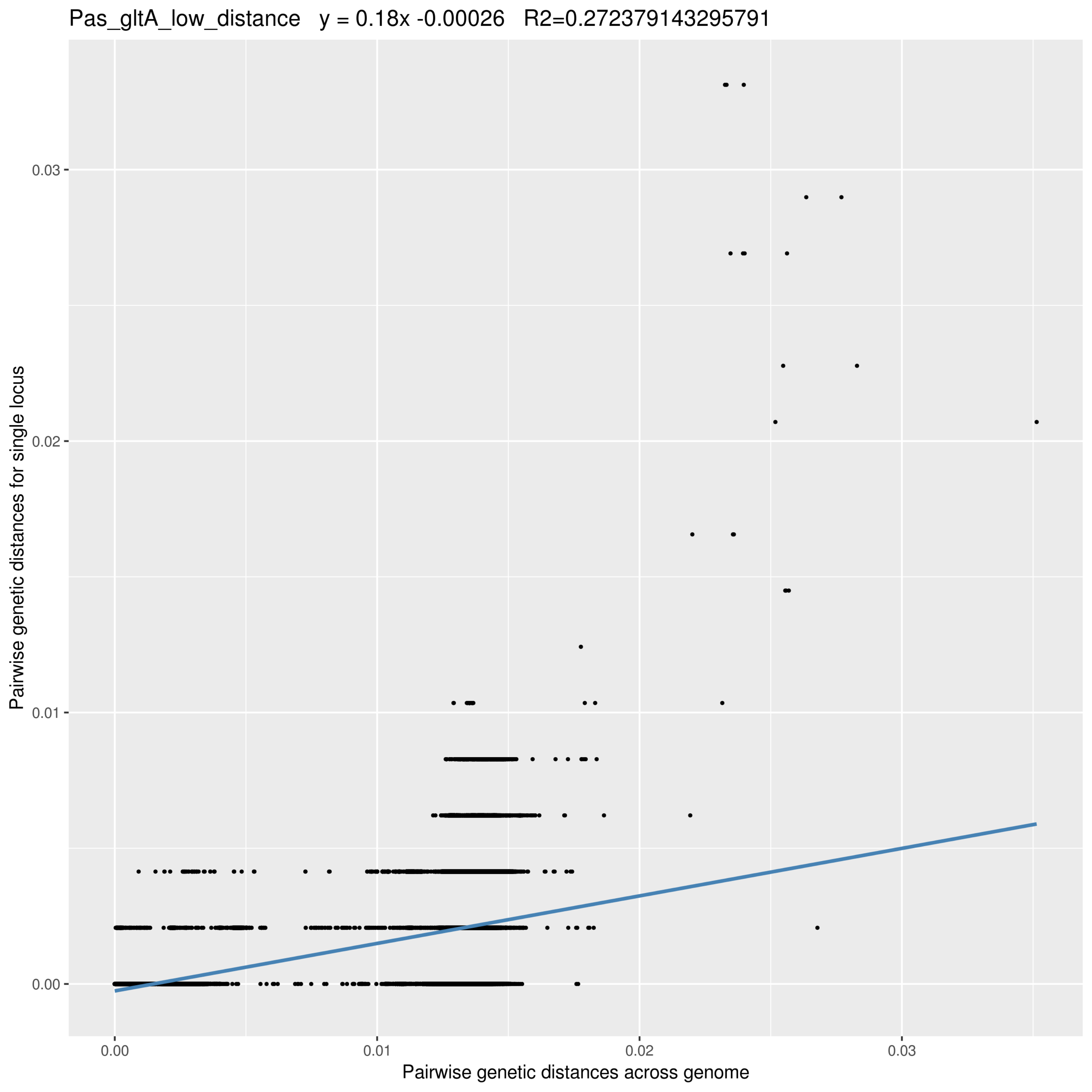
Pas_gltA_low_distance y = 0.18x -0.00026 R2=0.272379143295791
Pairwise genetic distances for single locus
0.03
0.02
0.01
0.00
0.00
0.01
0.02
0.03
Pairwise genetic distances across genome

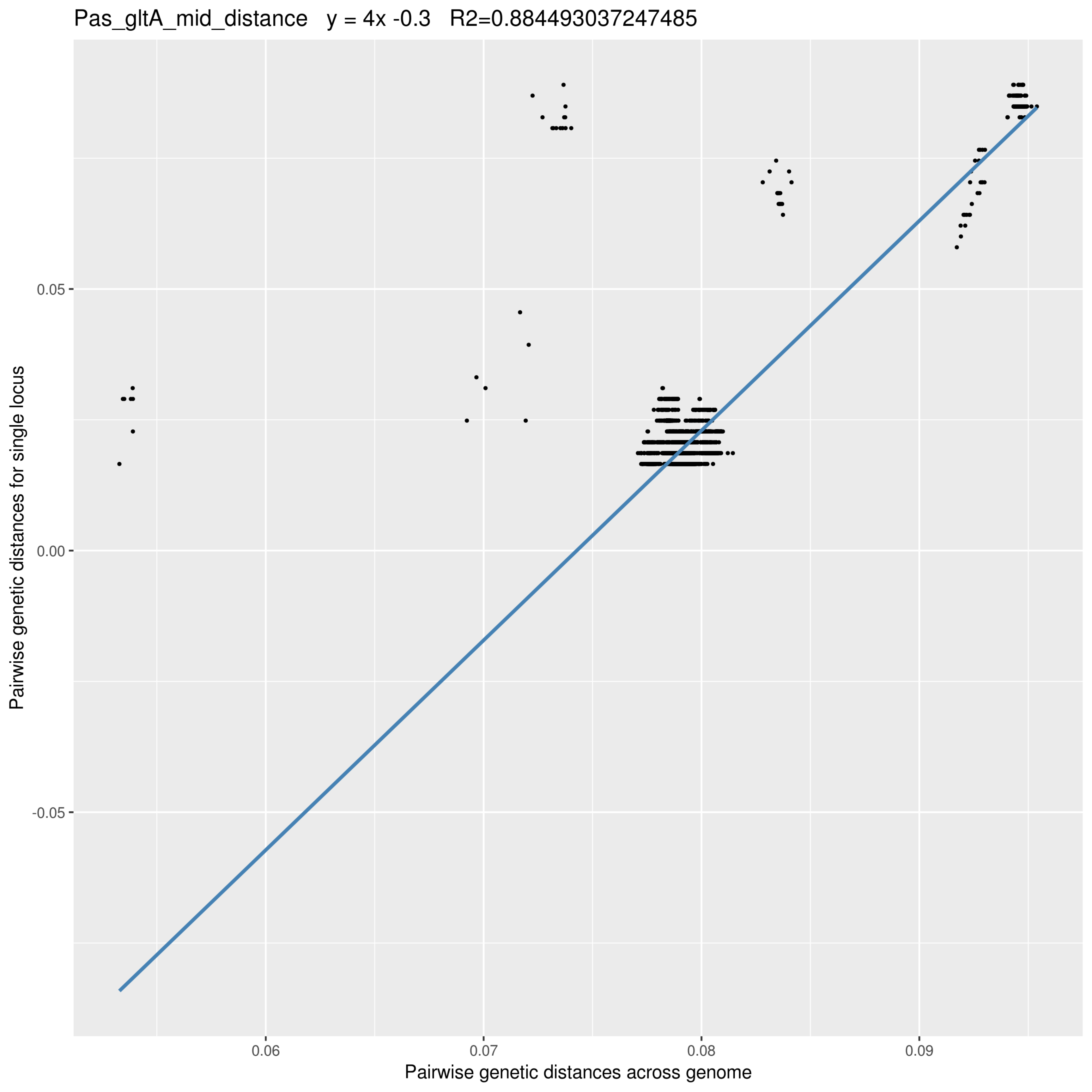
Pas_gltA_mid_distance   y = 4x -0.3   R2=0.884493037247485
Pairwise genetic distances for single locus
0.05
0.00
-0.05
0.06
0.07
0.08
0.09
Pairwise genetic distances across genome

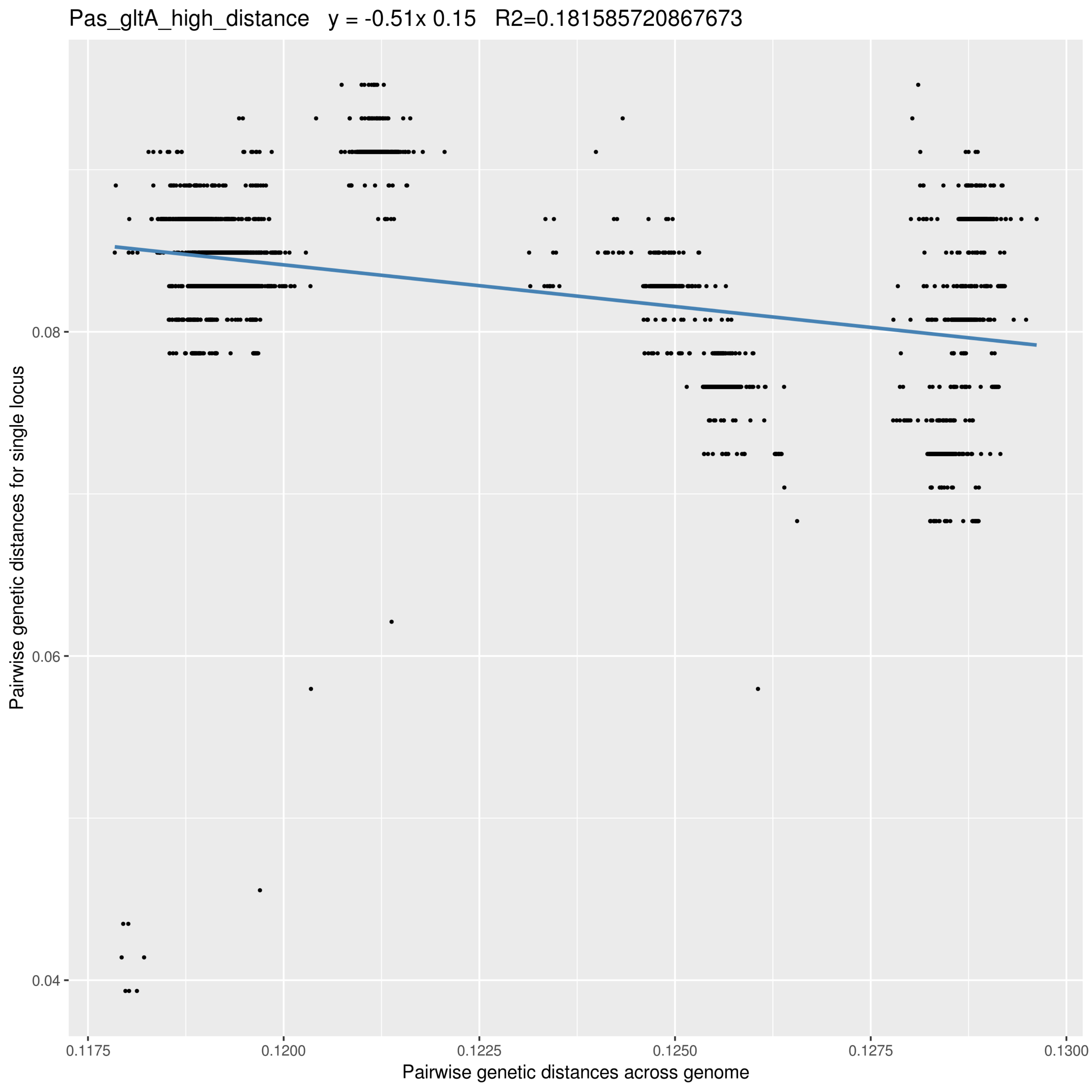
Pas_gltA_high_distance   y = -0.51x 0.15   R2=0.181585720867673
Pairwise genetic distances for single locus
0.08
0.06
0.04
0.1175
0.1200
0.1225
0.1250
0.1275
0.1300
Pairwise genetic distances across genome

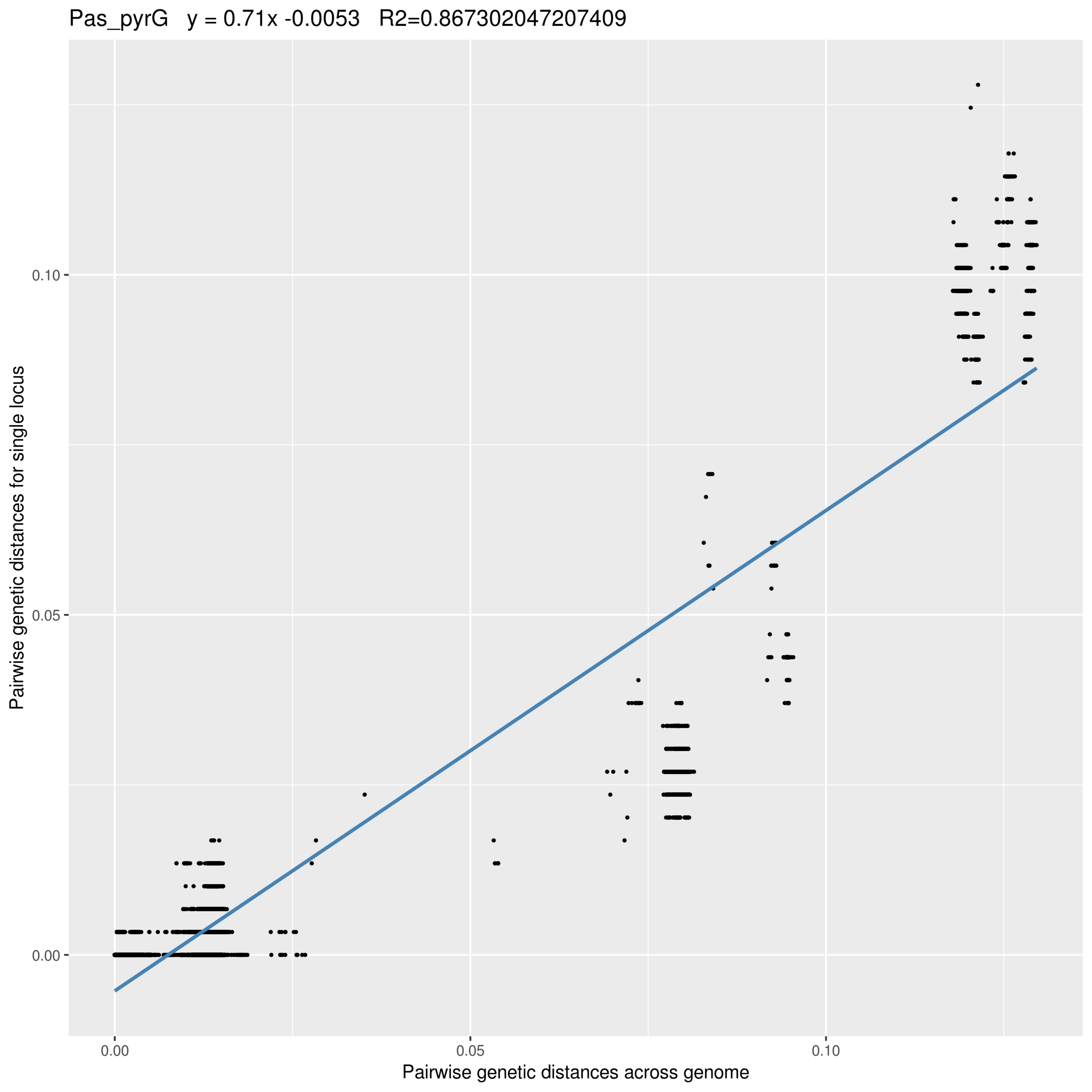

Pas_pyrG   y = 0.71x -0.0053   R2=0.867302047207409
0.10
0.05
0.00
Pairwise genetic distances for single locus
0.00
0.05
0.10
Pairwise genetic distances across genome

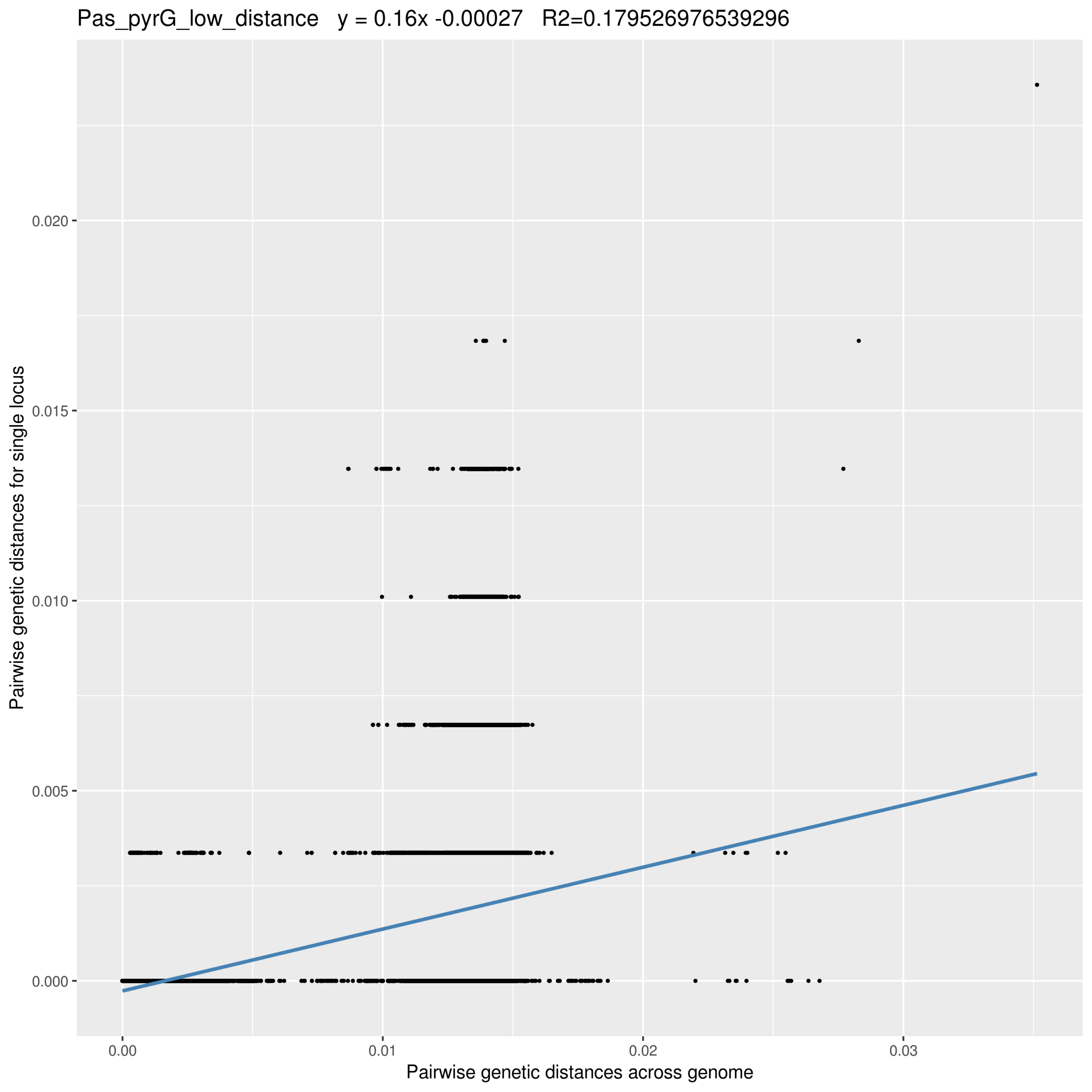
Pas_pyrG_low_distance   y = 0.16x -0.00027   R2=0.179526976539296
Pairwise genetic distances for single locus
0.020
0.015
0.010
0.005
0.000
0.00
0.01
0.02
0.03
Pairwise genetic distances across genome

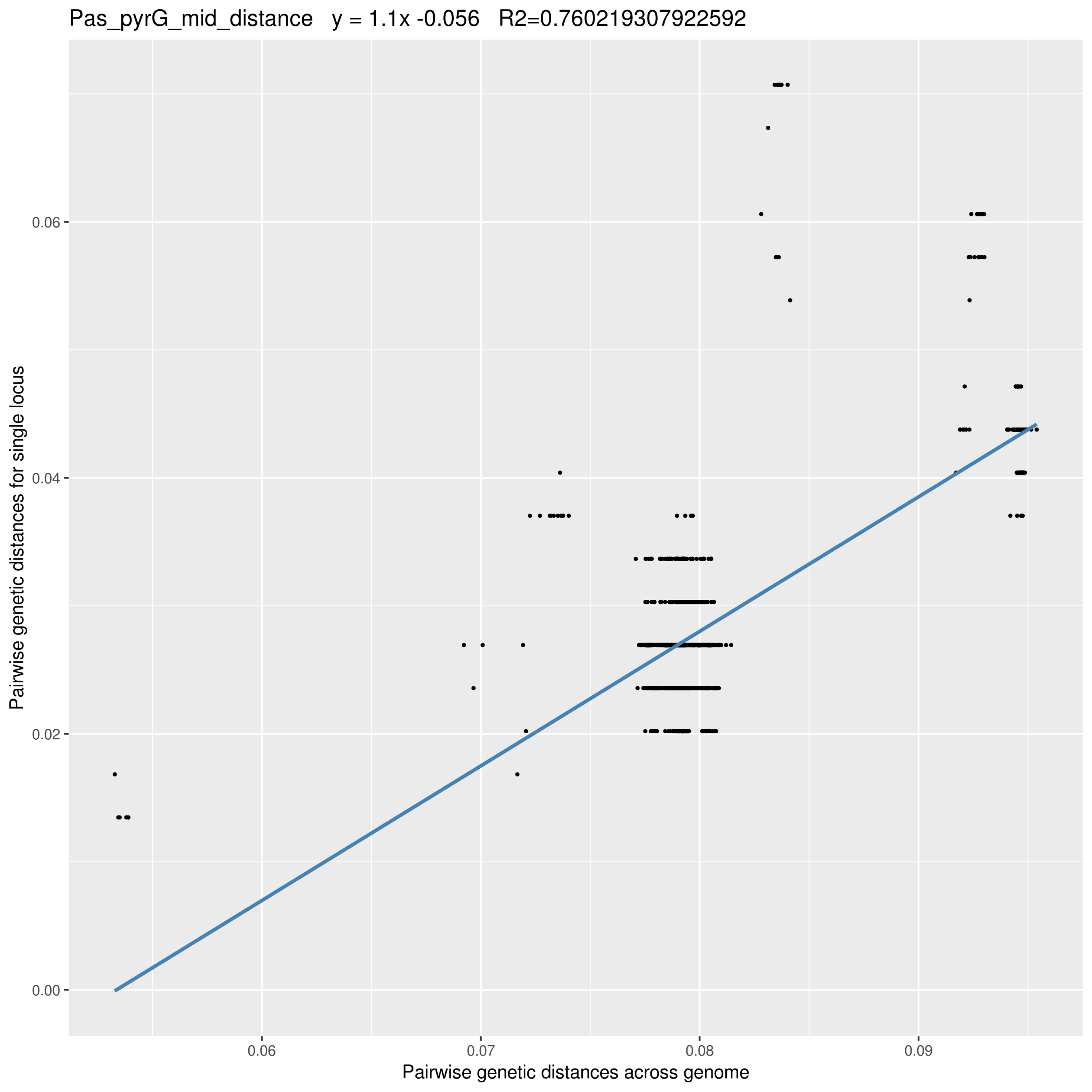

Pas_pyrG_mid_distance   y = 1.1x -0.056   R2=0.760219307922592
0.00
0.02
0.04
0.06
Pairwise genetic distances for single locus
0.06
0.07
0.08
0.09
Pairwise genetic distances across genome

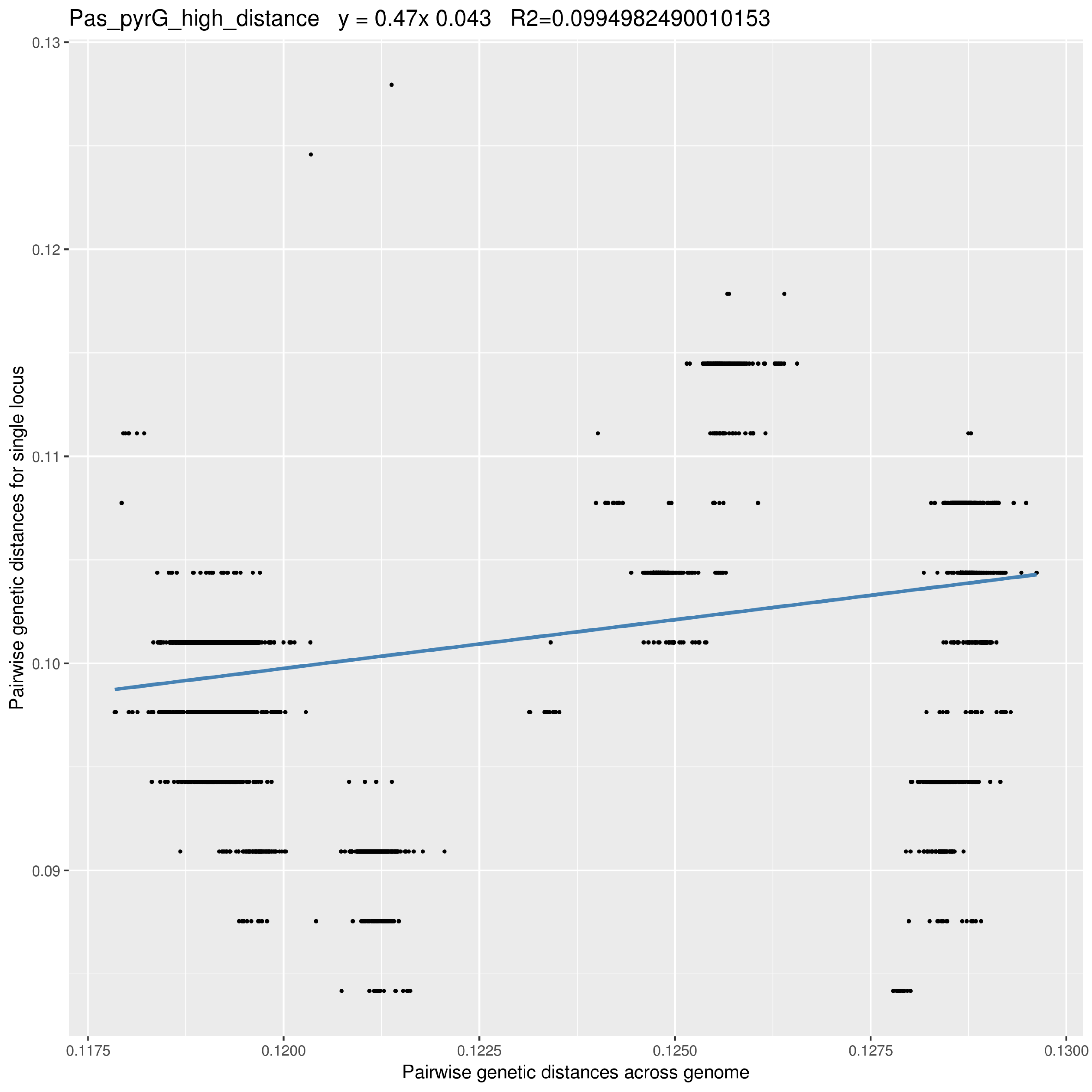

Pas_pyrG_high_distance   y = 0.47x 0.043   R2=0.0994982490010153
Pairwise genetic distances for single locus
0.13
0.12
0.11
0.10
0.09
0.1175
0.1200
0.1225
0.1250
0.1275
0.1300
Pairwise genetic distances across genome

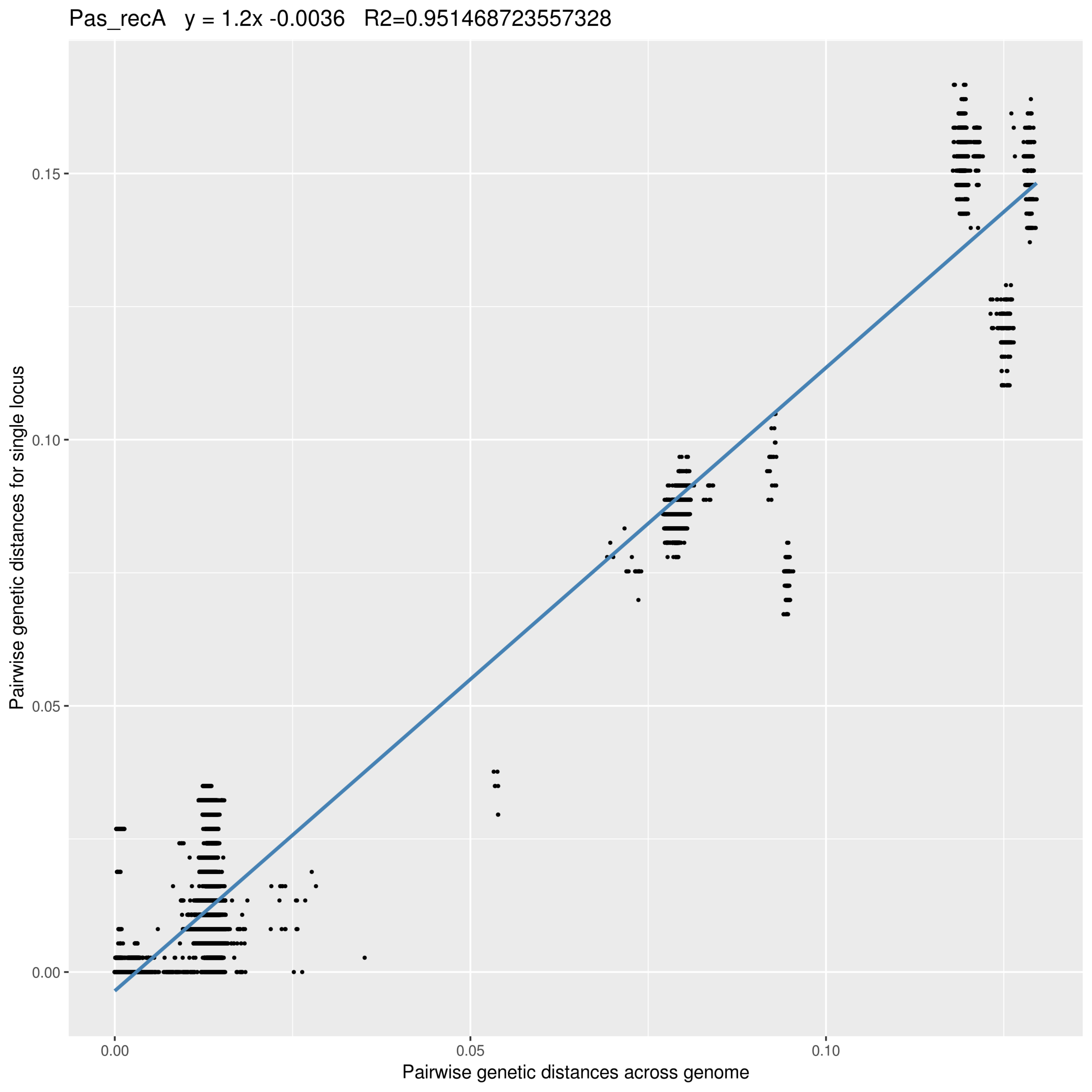

Pas_recA   y = 1.2x -0.0036   R2=0.951468723557328
Pairwise genetic distances for single locus
0.15
0.10
0.05
0.00
0.00
0.05
0.10
Pairwise genetic distances across genome

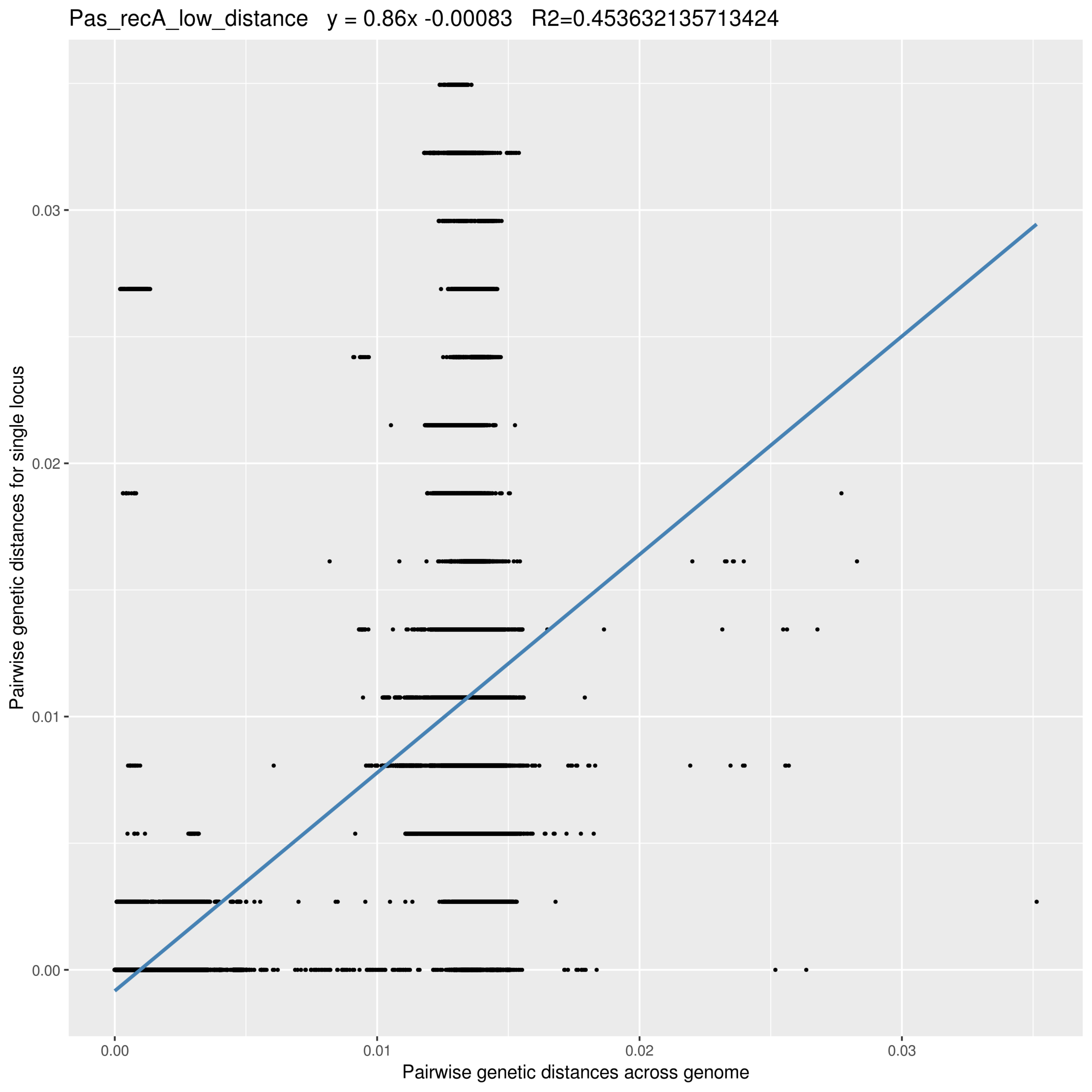

Pas_recA_low_distance   y = 0.86x -0.00083   R2=0.453632135713424
Pairwise genetic distances for single locus
0.03
0.02
0.01
0.00
0.00
0.01
0.02
0.03
Pairwise genetic distances across genome

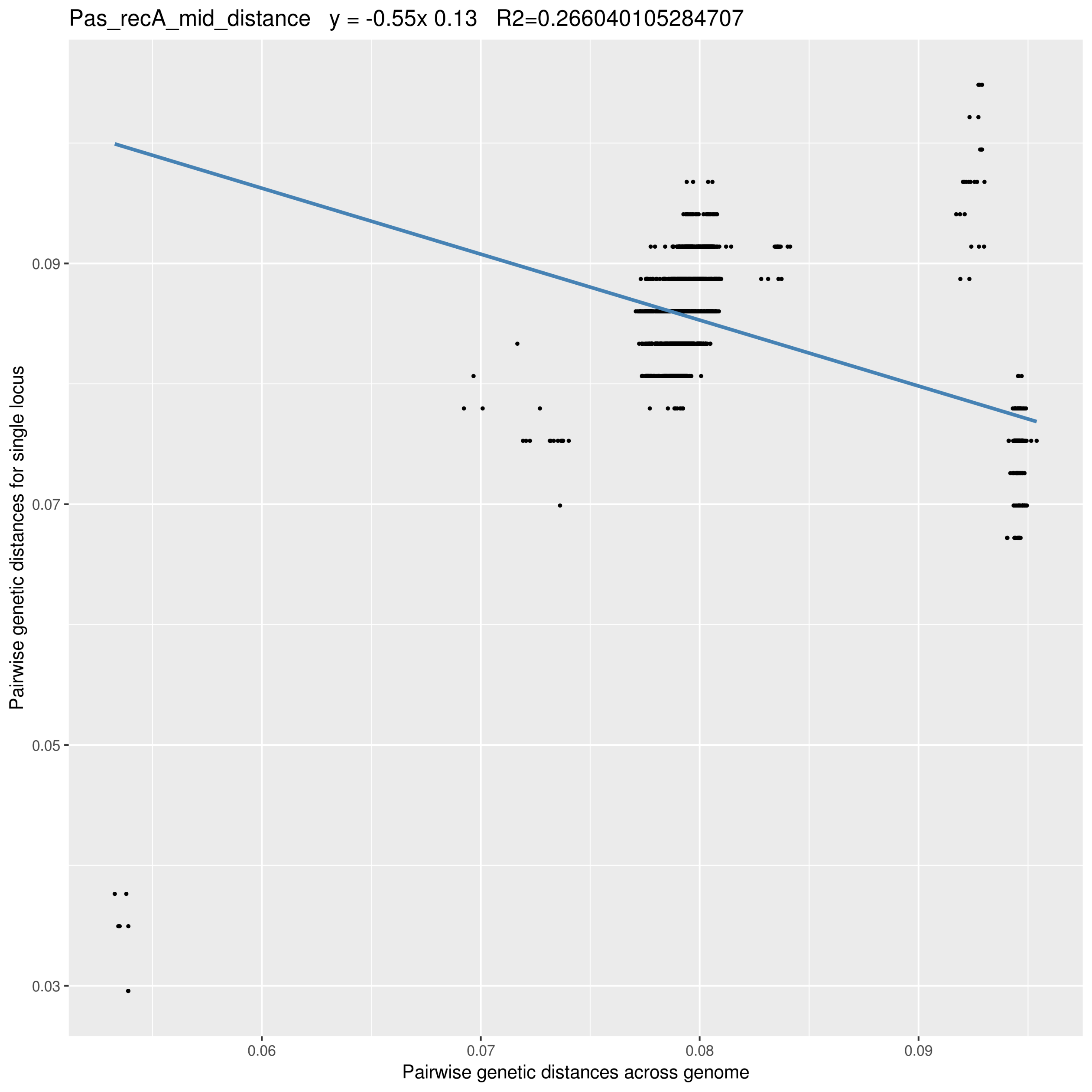

Pas_recA_mid_distance y = -0.55x 0.13 R2=0.266040105284707
Pairwise genetic distances for single locus
0.09
0.07
0.05
0.03
0.06
0.07
0.08
0.09
Pairwise genetic distances across genome

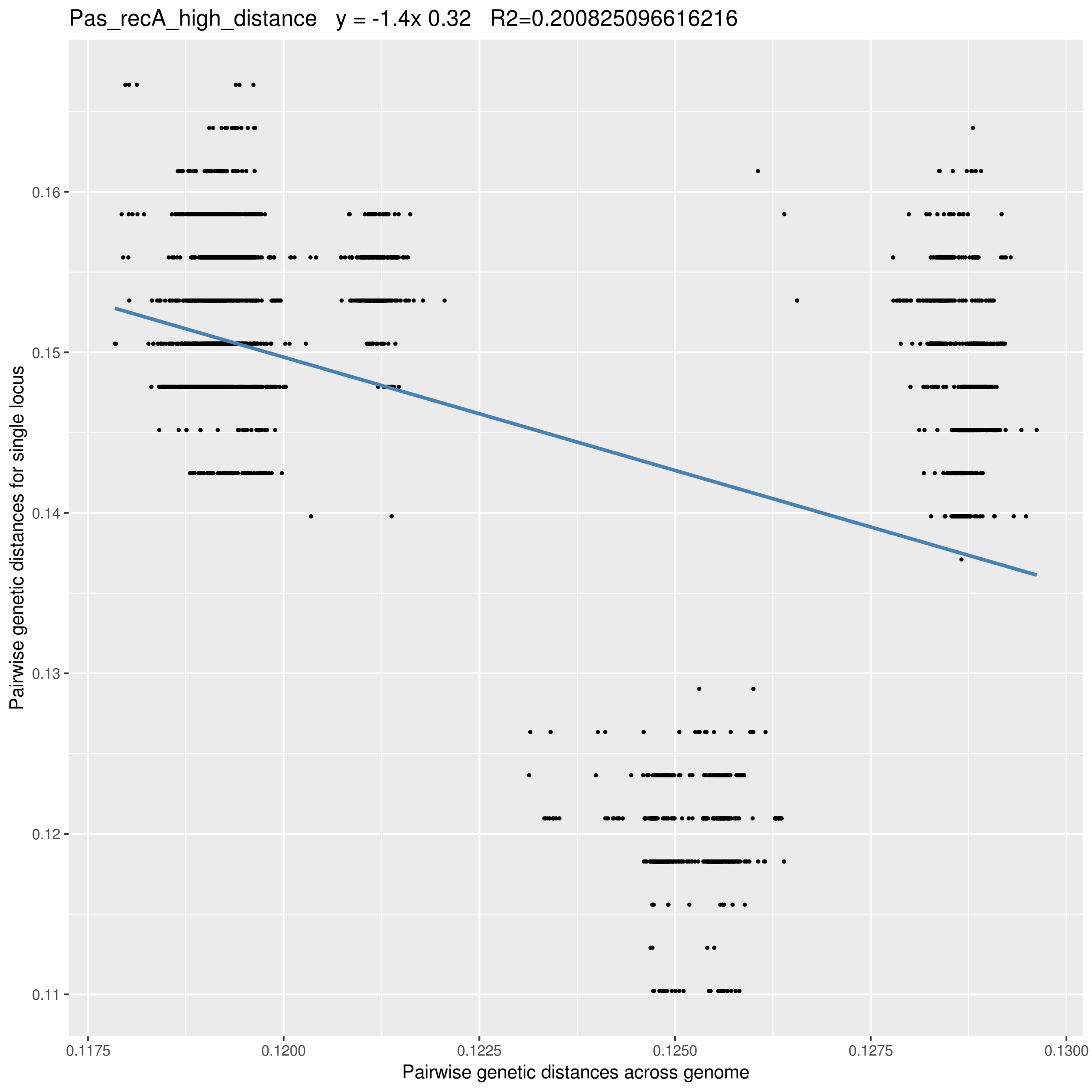

Pas_recA_high_distance y = -1.4x 0.32 R2=0.200825096616216
Pairwise genetic distances for single locus
0.16
0.15
0.14
0.13
0.12
0.11
0.1175
0.1200
0.1225
0.1250
0.1275
0.1300
Pairwise genetic distances across genome

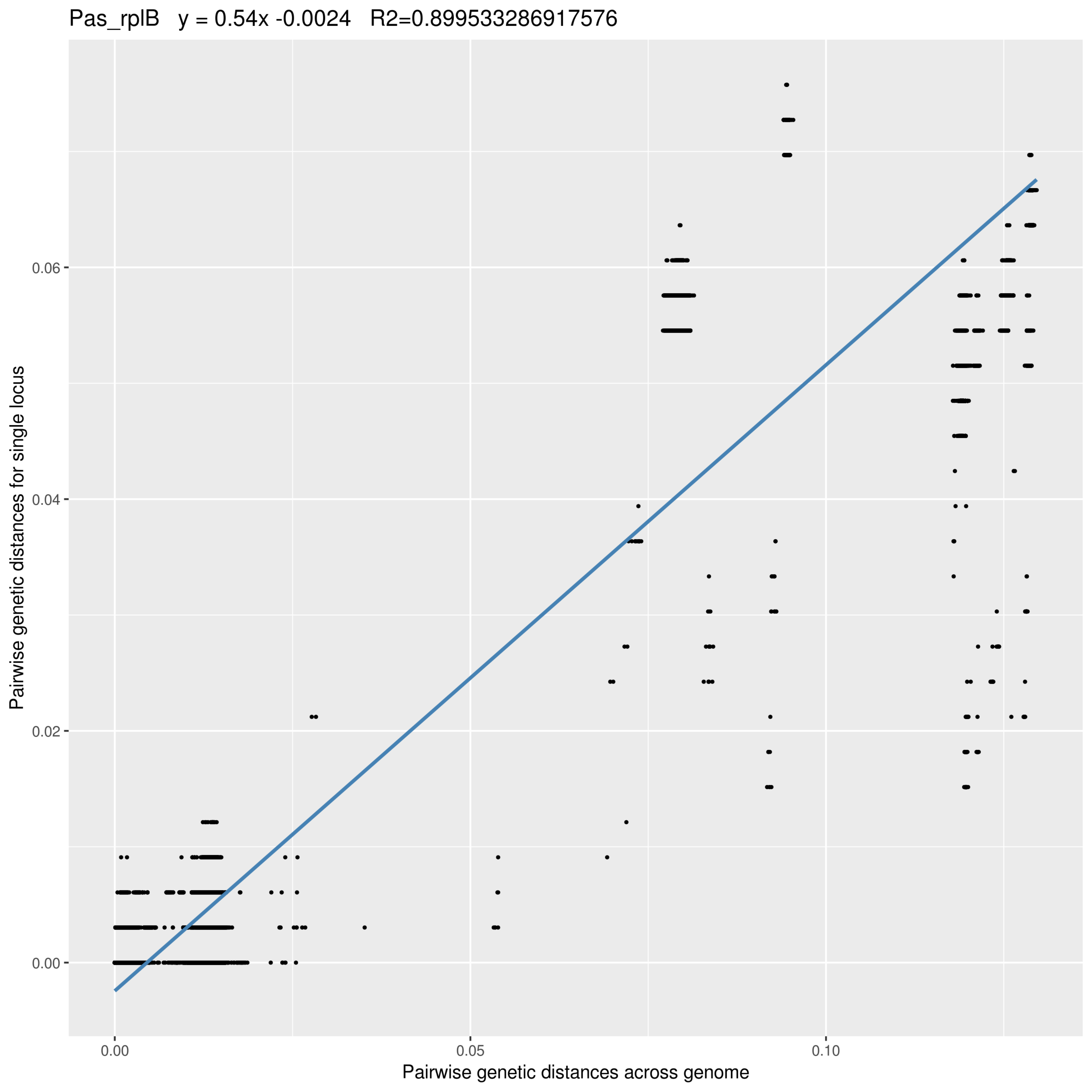
Pas_rplB y = 0.54x -0.0024 R2=0.899533286917576
Pairwise genetic distances for single locus
0.00
0.02
0.04
0.06
0.00
0.05
0.10
Pairwise genetic distances across genome

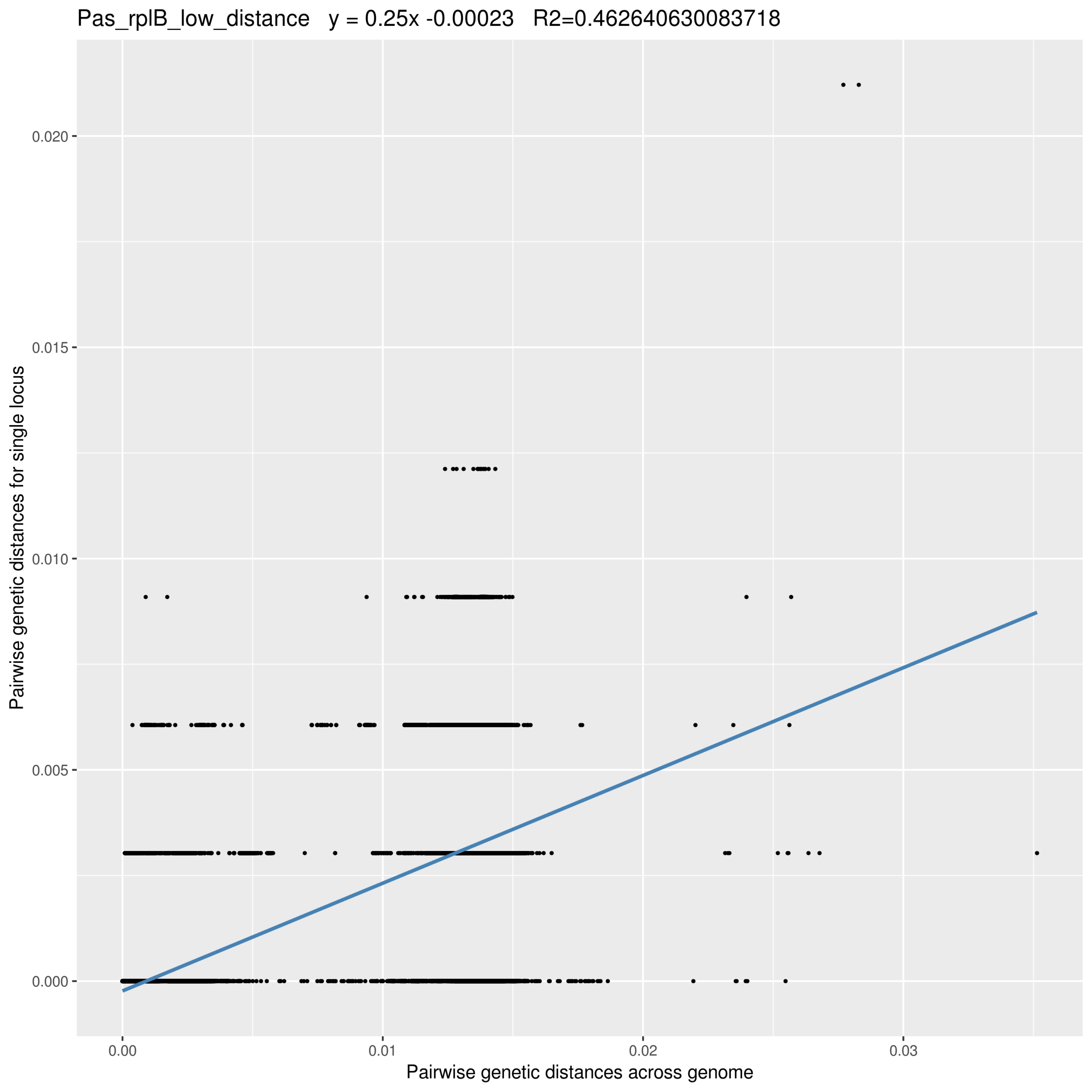

Pas_rplB_low_distance   y = 0.25x -0.00023   R2=0.462640630083718
Pairwise genetic distances for single locus
0.020
0.015
0.010
0.005
0.000
0.00
0.01
0.02
0.03
Pairwise genetic distances across genome

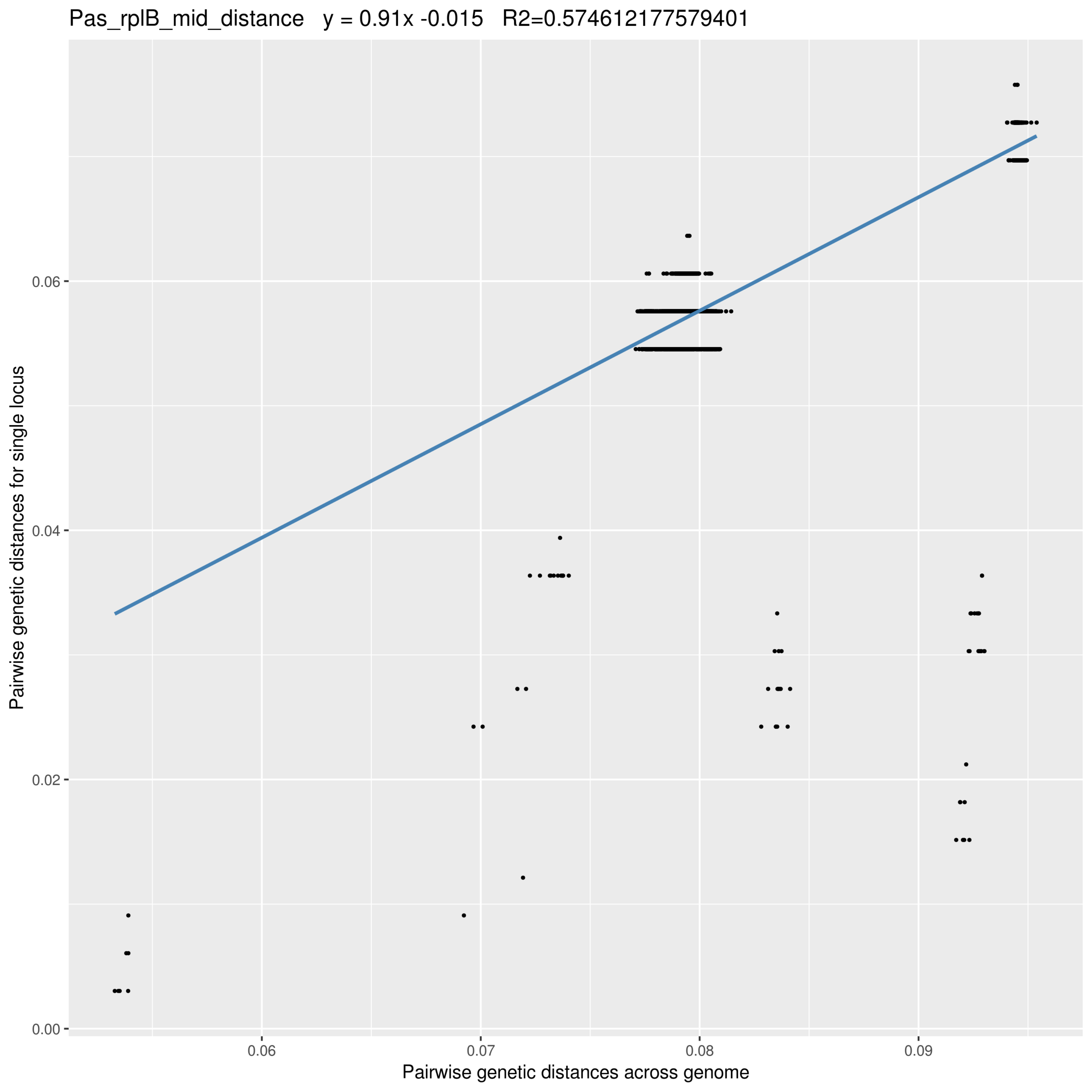

Pas_rplB_mid_distance y = 0.91x -0.015 R2=0.574612177579401
Pairwise genetic distances for single locus
0.00
0.02
0.04
0.06
0.06
0.07
0.08
0.09
Pairwise genetic distances across genome

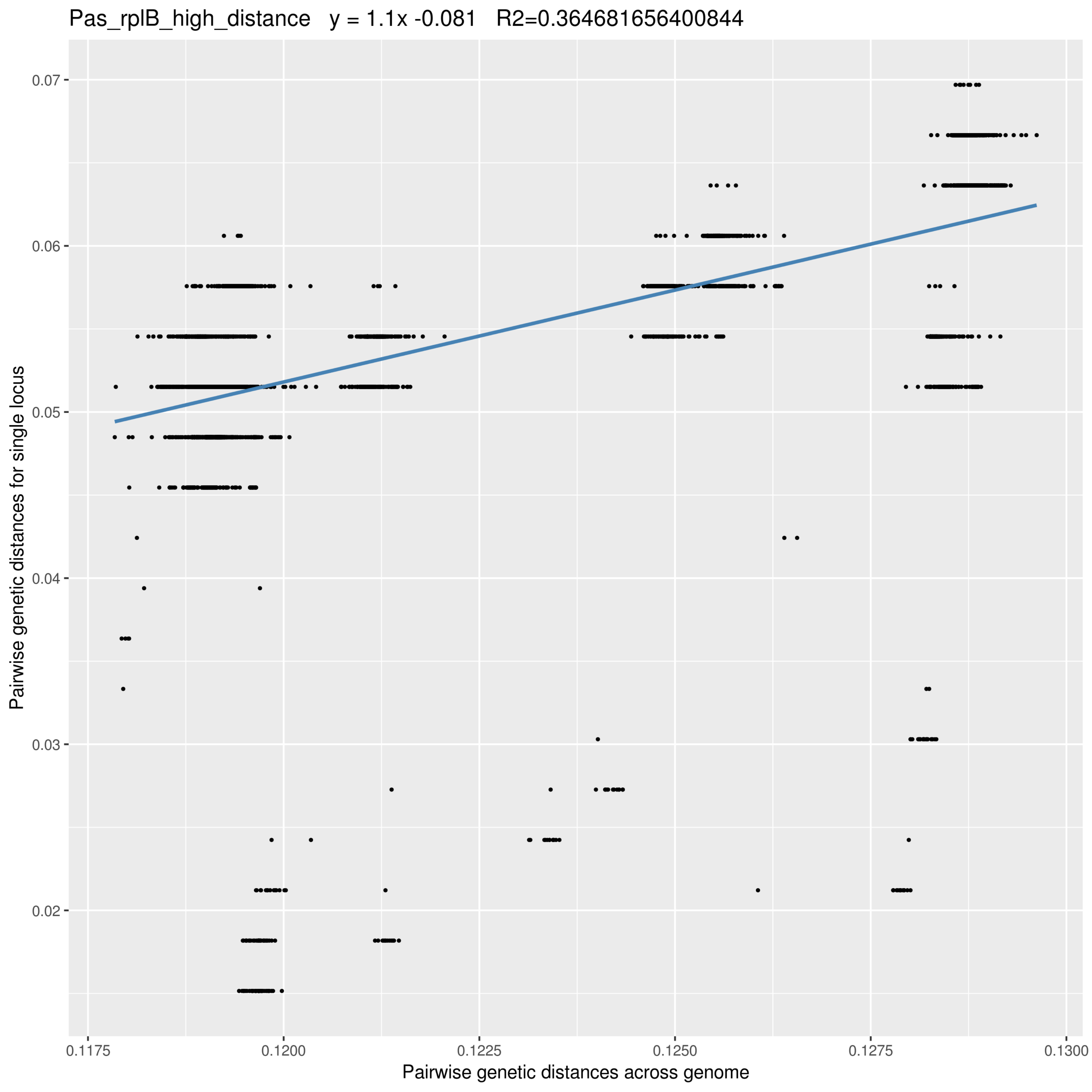

Pas_rplB_high_distance y = 1.1x -0.081 R2=0.364681656400844
Pairwise genetic distances for single locus
0.07
0.06
0.05
0.04
0.03
0.02
0.1175
0.1200
0.1225
0.1250
0.1275
0.1300
Pairwise genetic distances across genome

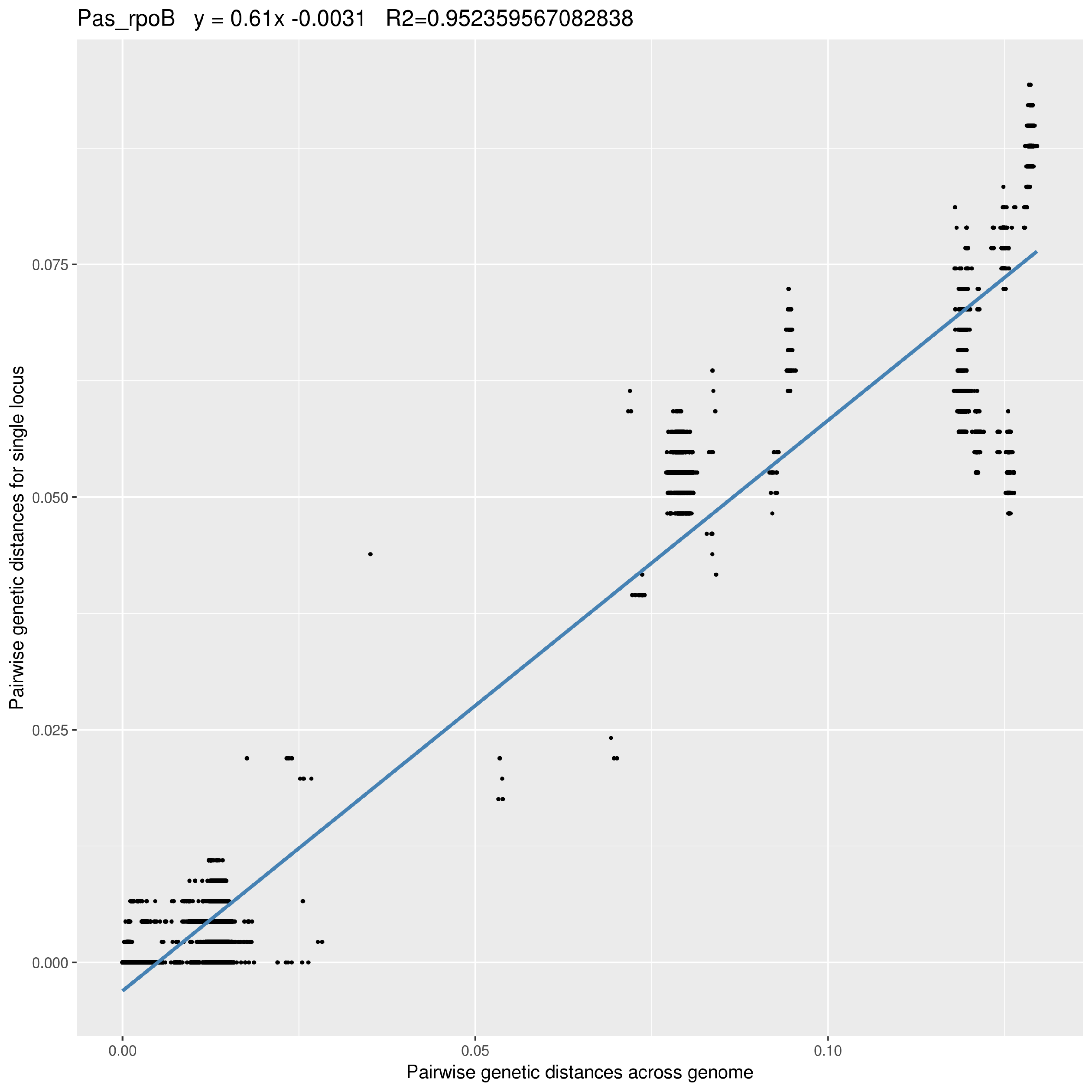

Pas_rpoB   y = 0.61x -0.0031   R2=0.952359567082838
Pairwise genetic distances for single locus
0.075
0.050
0.025
0.000
0.00
0.05
0.10
Pairwise genetic distances across genome

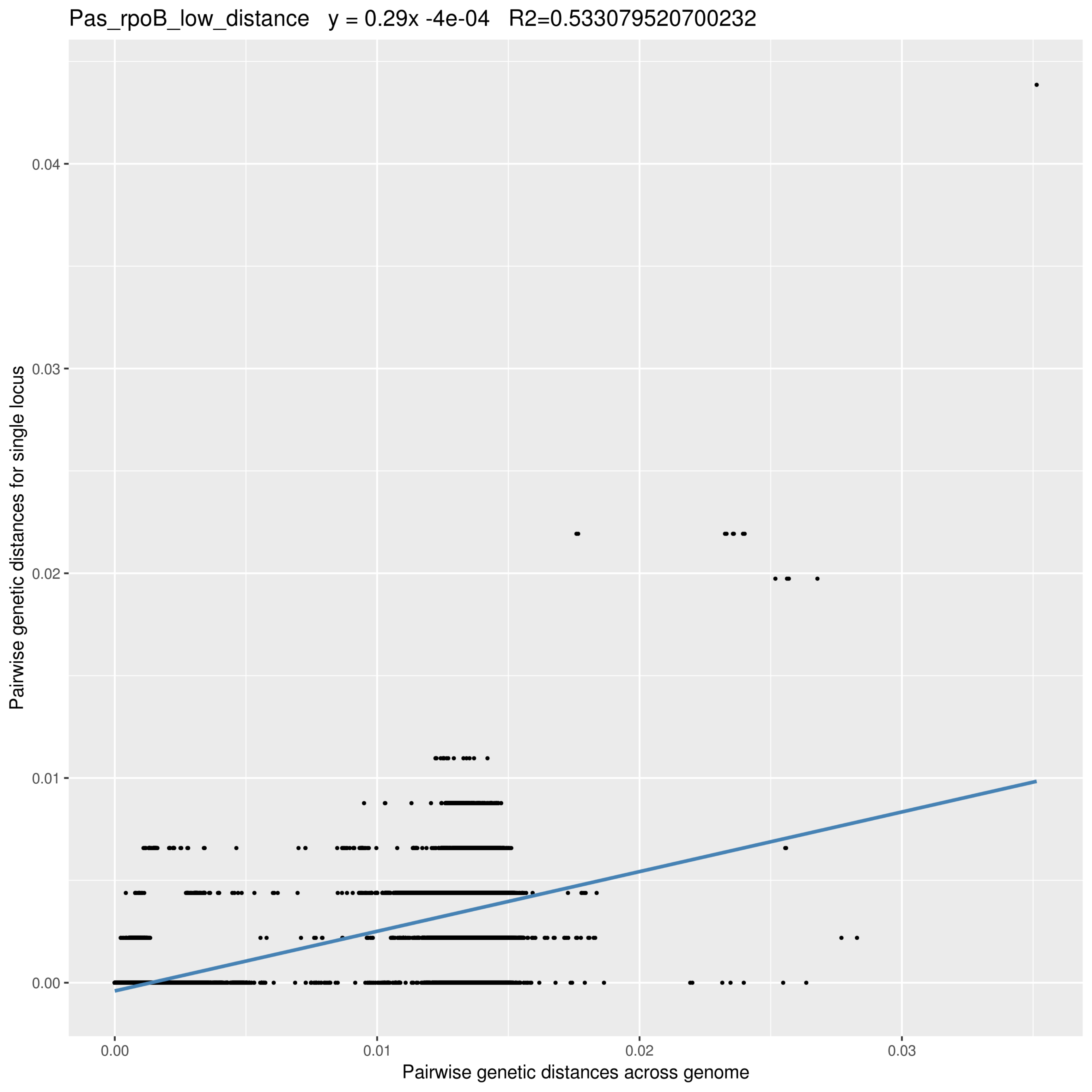

Pas_rpoB_low_distance   y = 0.29x -4e-04   R2=0.533079520700232
Pairwise genetic distances for single locus
0.00
0.01
0.02
0.03
0.04
Pairwise genetic distances across genome
0.00
0.01
0.02
0.03

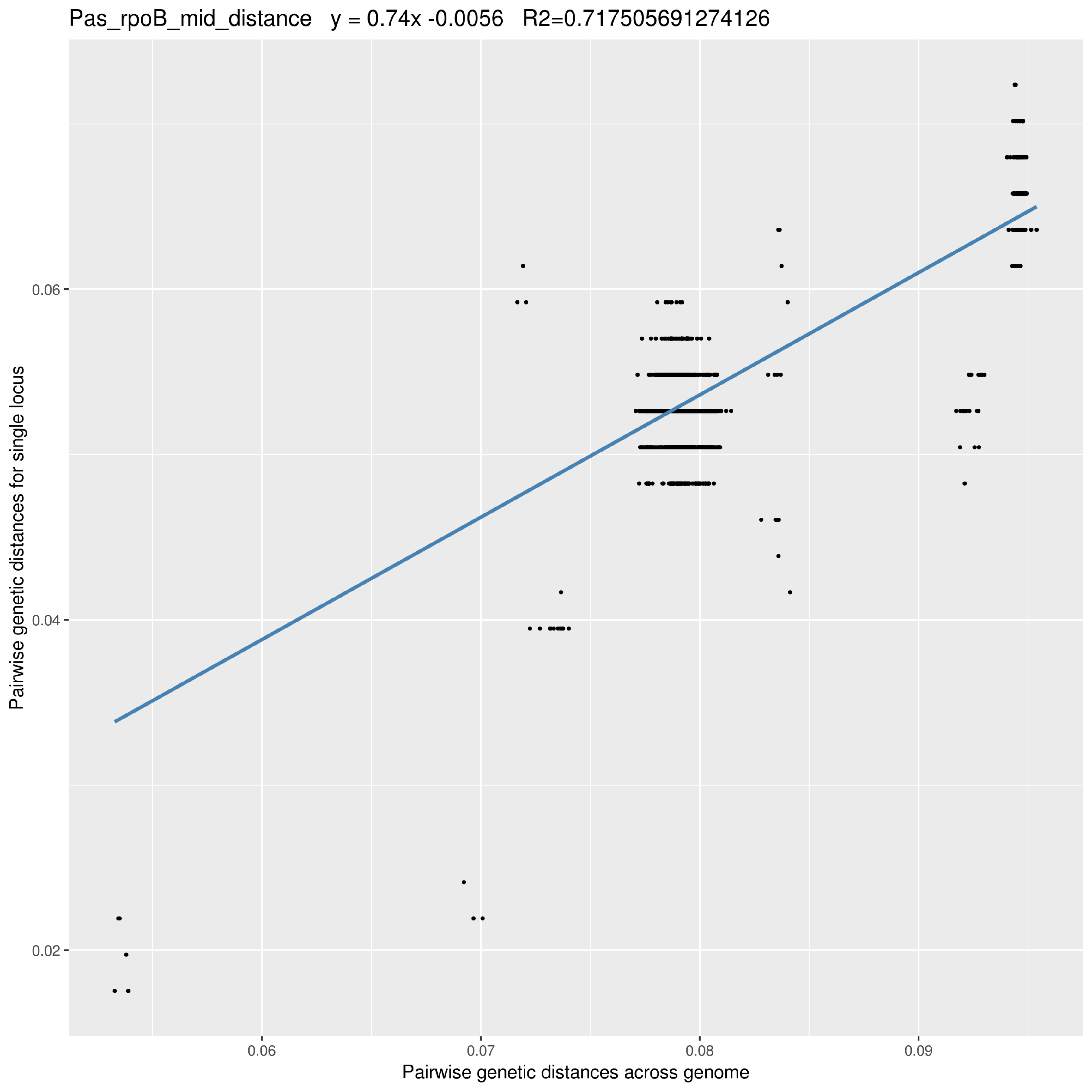

Pas_rpoB_mid_distance y = 0.74x -0.0056 R2=0.717505691274126
Pairwise genetic distances for single locus
0.02
0.04
0.06
0.06
0.07
0.08
0.09
Pairwise genetic distances across genome

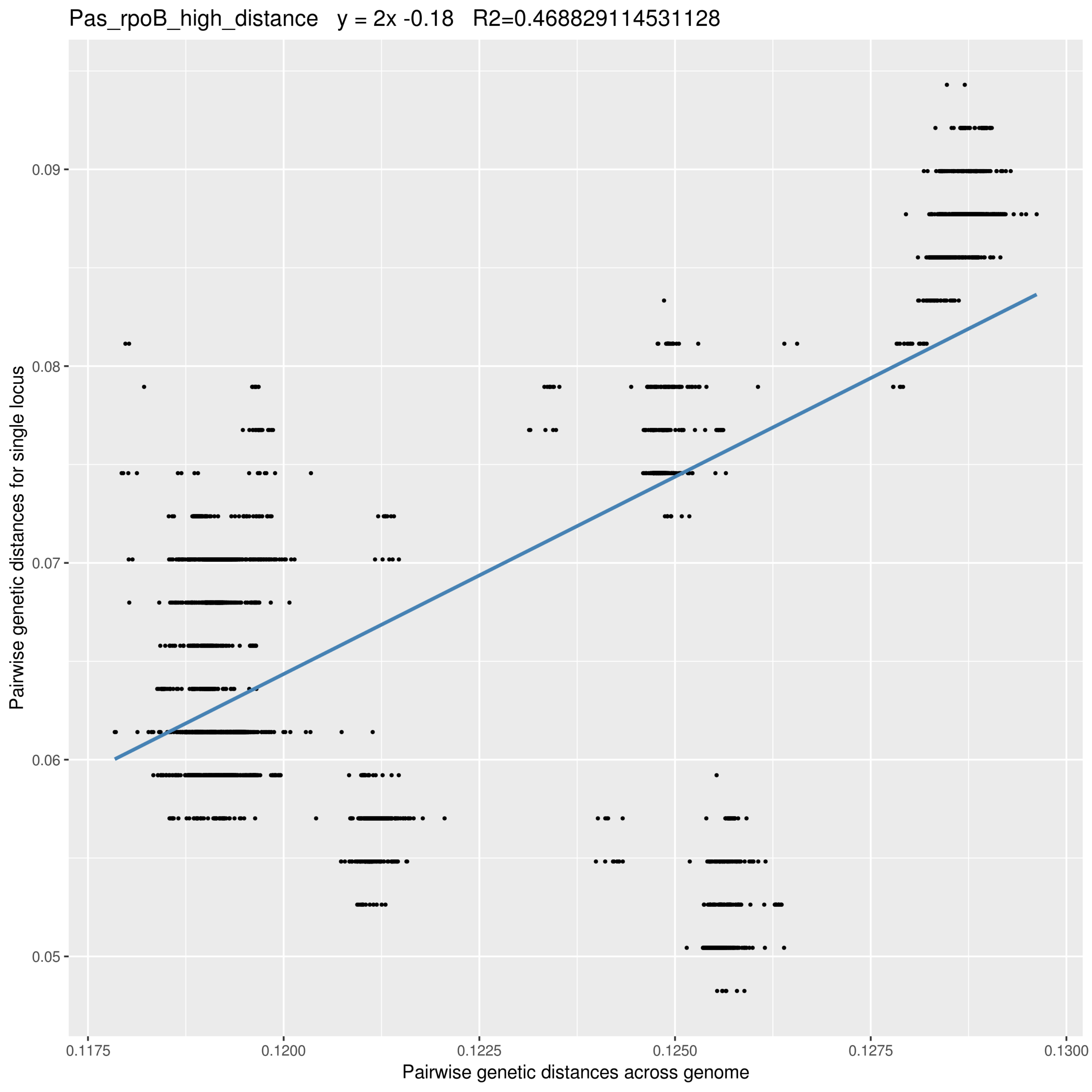

Pas_rpoB_high_distance y = 2x -0.18 R2=0.468829114531128
Pairwise genetic distances for single locus
0.09
0.08
0.07
0.06
0.05
0.1175
0.1200
0.1225
0.1250
0.1275
0.1300
Pairwise genetic distances across genome
